# The Training of Short Distance Sprint Performance in Football Code Athletes: A Systematic Review and Meta-Analysis

**DOI:** 10.1007/s40279-020-01372-y

**Published:** 2020-11-27

**Authors:** Ben Nicholson, Alex Dinsdale, Ben Jones, Kevin Till

**Affiliations:** 1 Carnegie Applied Rugby Research (CARR) Centre, Carnegie School of Sport, Leeds Beckett University, Room G08, Cavendish Hall, Headingley Campus, Leeds, LS6 3QS UK; 2 Yorkshire Carnegie Rugby Union Club, Leeds, UK; 3 Leeds Rhinos Rugby League Club, Leeds, UK; 4 England Performance Unit, The Rugby Football League, Leeds, UK; 5 School of Science and Technology, University of New England, Armidale, NSW Australia; 6 Division of Exercise Science and Sports Medicine, Department of Human Biology, Faculty of Health Sciences, The University of Cape Town and the Sports Science Institute of South Africa, Cape Town, South Africa

## Abstract

**Background:**

Short-sprint (≤ 20 m) performance is an important quality for success in the football codes. Therefore, developing an evidence base for understanding training methods to enhance short-sprint performance is key for practitioners. However, current systematic reviews are limited by (1) a lack of focus on football code athletes, (2) a lack of consideration of all training modalities and (3) a failure to account for the normal training practices undertaken by intervention groups within their analysis. Therefore, this review aimed to (1) conduct a systematic review of the scientific literature evaluating training interventions upon short-sprint performance within football code athletes, (2) undertake a meta-analysis to assess the magnitude of change of sport-sprint performance following training interventions and (3) identify how moderator variables affect the training response.

**Methods:**

A systematic search of electronic databases was conducted. A random-effects meta-analysis was performed to establish standardised mean difference with 95% confidence intervals. This identified the magnitude and direction of the individual training effects of intervention subgroups (primary, secondary, combined-specific, tertiary and combined training methods) on short-sprint performance while considering moderator variables (i.e., football code, sex, age, playing standard, phase of season).

**Results:**

121 studies met the inclusion criteria, totalling 3419 athletes. Significant improvements (small-large) were found between pre- and post-training in short-sprint performance for the combined, secondary, tertiary and combined-specific training methods. No significant effect was found for primary or sport only training. No individual mode was found to be the most effective. Between-subgroup analysis identified that football code, age, playing standard and phase of season all moderated the overall magnitude of training effects.

**Conclusions:**

This review provides the largest systematic review and meta-analysis of short-sprint performance development methods and the only one to assess football code athletes exclusively. Practitioners can apply combined, secondary and tertiary training methods to improve short-sprint performance within football code athletes. The application of sport only and primary methods does not appear to improve short-sprint performance. Regardless of the population characteristics, short-sprint performance can be enhanced by increasing either or both the magnitude and the orientation of force an athlete can generate in the sprinting action.

**Trial Registration:**

OSF registration https://osf.io/kshqn/.

**Electronic supplementary material:**

The online version of this article (10.1007/s40279-020-01372-y) contains supplementary material, which is available to authorized users.

## Key Points

**Table:** 

Short-sprint performance (0–5, 0–10 and 0–20 m) of football code athletes can be enhanced through secondary (i.e., resisted or assisted sprinting), tertiary (i.e., strength, power and plyometrics) and combined (i.e., primary [i.e., sprinting, running drills] or secondary and tertiary) training methods. Combined specific training methods (i.e., primary and secondary methods) improved short-sprint performance (0–5 and 0–10 m). However, the sport only and primary methods alone do not appear to enhance short-sprint performance. No individual mode was found to be the most effective.
Independent of the population characteristics, findings suggest that practitioners should develop either or both the magnitude and the orientation of forces an athlete can generate and express in the sprinting action to improve short-sprint performance.
Research has mainly been undertaken within male soccer athletes including some form of tertiary training methods (e.g., strength, power and plyometrics training).

## Introduction

The development of short-distance sprint (short-sprint) performance (i.e., 5–20 m) is a vital component of athletic performance within the football codes [1]. A football code athlete’s ability to produce high levels of linear speed (at various short distances) has been shown to be a factor differentiating within playing standards [2–4] and age categories [2, 4, 5], as well as being associated with success in key attacking and defensive performance indicators across football codes (i.e., rugby league [6, 7], Australian rules football [8], rugby union [9, 10], rugby sevens [11] and soccer [12]). This body of evidence emphasises the importance of short-sprint performance for football performance and player development. Due to the high frequency of short-sprints with incomplete rest, present in the football codes, repeat sprint ability (RSA) is also considered an important factor for an athlete’s sprinting capabilities [1, 13]. Previous research has reported small-large relationships (*r* = 0.44–0.86) between short-sprint performance (0–5 m, 0–10 m, 0–20 m) and RSA [14, 15]. As such maximum short-sprint performance may also positively influence RSA. However, the physical adaptations required to develop these qualities are distinctly separate and thus will not be considered within this review [13]. Understanding the most effective, evidence-based methods for developing sprint performance is a challenge for all practitioners involved in the football codes, due to the concurrent developmental need of multiple physical qualities (e.g., speed, strength, power and endurance) alongside technical and tactical skills [1, 2, 16]. Hence, concurrent or conflicting training systems exist. This differs from track sprinters and non-athletic populations and needs to be considered when prescribing training for football code athletes.

Although definitions vary, sprint performance is typically split into two components: acceleration and maximal sprinting velocity phases [17–26]. Despite being sequential, moderate to large correlations have been identified between acceleration and maximum sprinting velocity in football code athletes (*r* = 0.56–0.87). This suggests that even though these phases are related, separate physical qualities and mechanical parameters determine sprint performance [21, 27–30]. Mechanically, athletes exhibit relatively lower stride frequencies (i.e., longer foot contacts and shorter flight times), shorter stride lengths and an increased forward trunk lean when accelerating [18, 31–33]. Notably, football code athletes typically achieve maximal sprint velocity (*V*_max_) at shorter distances (15–40 m vs. 40–60 m respectively) with lower maximal velocities (~ 7–10 vs. > 12 m·s^−1^) compared to well-trained, male elite sprinters [18, 21, 30, 34–36]. Furthermore, football code athletes attain a greater percentage of maximal velocity at shorter distances (e.g., 90% at 13.7 m in American Football [30], 96% at 21 m in rugby [18]). As a result practitioners and researchers typically use linear short-sprint performance outcomes (i.e., time to completion or peak velocity achieved) as a proxy measure for acceleration performance (e.g., 0–5 m, 0–10 m, 0–20 m) across its sequential phases [16, 37, 38]. Therefore, as a population, football code athletes exhibit different physical and technical approaches to sprinting [34], when compared to well-trained sprinters. This highlights the need for specifically targeted sprint-based research within the football codes.

Research has previously reported that short-sprint performance is a trainable quality in football code athletes (i.e., soccer, 20 m performance) [39]. However, the training response in short-sprint performance is highly variable [40–42]. Previous research has shown that training responses are mode-specific with distance specific performance changes (e.g., 0–10 m and 0–20 m) associated with phase-specific adaptations [43]. Training modes have been classified based on task specificity into the following subgroups; primary (e.g., sprint technique, sprinting), secondary (e.g., resisted or assisted sprinting) or tertiary (e.g., non-specific methods including resistance training and plyometrics) [44]. Despite the importance of short-sprint performance, it is currently unclear both individually and across football codes what method is best to enhance performance. Hence, developing short-sprint performance is a collective problem across codes. Conducting a cross-football codes systematic review would allow a more comprehensive overview of the available literature than a single sport, while also comparing best methods of developing short-sprint performance. However**,** the magnitude and direction of the training effect can be affected by “moderator” variables presenting fluctuating changes based on population characteristics such as the sport [45], age [46] and sex [47] of the athlete, and training phase (e.g., pre-season [48]). Therefore, it is important to identify the moderator variables and evaluate the extent that they may affect the resultant training effect when training short-sprint speed [49].

Despite the plethora of research evaluating the effectiveness of short-sprint training interventions alongside several systematic reviews and meta-analyses, several limitations exist in the current literature. These are (1) no review focuses upon only football code athletes, instead including sprinters and non-athletes [39, 43, 46, 48, 50–57]; (2) no study examines all training modalities across football code athletes on short-sprint performance [39, 43, 46, 48, 50–57]; and (3) previous systematic reviews and meta-analyses [39, 43, 48] fail to account for the normal training practices undertaken by training intervention groups (e.g., training categorised as a resisted sled intervention also including 2 strength sessions per week) within their reviews and analysis. These limitations heavily influence the interpretation and knowledge associated with sprint training interventions for applying effective evidence-based practices within football code athletes. Therefore, the aims of this review were to (1) systematically review the scientific literature evaluating the training interventions upon short-sprint performance (0–5 m, 0–10 m, 0–20 m) within football code athletes, (2) undertake a meta-analysis to assess the magnitude of change of short-sprint performance following training interventions; and (3) identify how moderator variables (i.e., football code, sex, playing standard, age and phase of season) affect the training response.

## Methods

### Design and Search Strategy

A systematic review and meta-analysis were conducted in accordance with the Preferred Reporting Items for Systematic Reviews and Meta-Analyses (PRISMA) statement and followed the Prospero guidelines [58]. Due to the nature of the project, the review protocol was prospectively registered on the database for open science framework (OSF) https://osf.io/kshqn/. A systematic search of electronic databases (PubMed, The Cochrane Library, MEDLINE, SPORTDiscus and CINAHL, via EBSCOhost) was conducted to identify original research articles published from the earliest available records up to and including 14/10/2019. Boolean search phrases were used to include search terms relevant to football code athletes (population), the training intervention (dependent variable) and the short-sprint performance outcomes (independent variable). Relevant keywords for each search term were determined through pilot searching (screening of titles/abstracts/keywords/full texts of previously known articles). Keywords were combined within-terms using the ‘OR’ operator, and the final search phrase was constructed by combining the three search terms using the ‘AND’ operator (Table 1).

**Table 1:** Database literature search strategy

Search term	Keywords
1. Sports population	“soccer” **OR** “football” **OR** “rugby” **OR** “futsal” (**NOT**/- “sprinters” **OR** “swimming” **OR** “cycling” **OR** “Paralympic”)
2. Training intervention	“sprinting” **OR** “sprint” **OR** “training” **OR** “speed” **OR** “resisted” **OR** “assisted” **OR** “resistance” **OR** “power” **OR** “strength” **OR** “plyometric” **OR** “weightlifting” **OR** "strongman" **OR** "technique" **OR** "weight" **OR** "sled" **OR** "intervention" **OR** "sprint mechanics"
3. Outcome measures	“sprint performance” **OR** “acceleration” **OR “v**elocity**”**
Search phrase:	1 **AND** 2 **AND** 3

Additional records were taken from the bibliographies of eligible studies and previous reviews which were explored using Google Scholar. Attempts were made to contact the authors of the selected articles to request any missing relevant information. No age or sex restrictions were imposed during the search stage.

### Study Selection

Duplicate records were identified and removed before the remaining records were screened against the predefined inclusion–exclusion criteria (Table 2). Studies were screened independently by two researchers (BN, AD). The screening of the journal articles was completed over two phases. Studies were initially excluded based on the content of the titles and abstracts followed by a full-text review. In the event of disagreement in the reviewer’s decision, reviewers met to come to an agreed decision on the paper. Disparities in study selection were resolved by a 3rd member (KT).

**Table 2:** Inclusion/exclusion criteria (title/abstract screening and full screening)

Criteria	Inclusion	Exclusion
1	Studies with human subjects and has a pre-and-post-outcome measure/s identifying sprint performance ≤ 20 m	Studies with non-human subjects and/or no pre-and-post-outcome measure/s identifying sprint performance > 20 m
2	Training intervention study with the training programme clearly outlined, designed to produce chronic adaptions (not acute). Interventions would include: specific sprint training (resisted, assisted, un-resisted sprinting, sprint mechanics and technique training), non-specific sprint training (strength, power, plyometric training, and non-traditional methods) and combined sprint training (combined specific, combined non-specific and combined mixed methods	Inappropriate study design—not an intervention study or an acute/post-activation study
3	Original research article	Reviews, surveys, opinion pieces, books, periodicals and editorials
4	Population—Football codes athletes. Football athletes would be defined as those who are competing within a football code. Football codes for inclusion: soccer, American football, Canadian football, Australian rules football, rugby union, rugby league, rugby sevens, Gaelic football, futsal	Non-football code sports (e.g., solo, racquet/bat, or combat sports), match officials, or non-athletic populations
5	Healthy, able-bodied, non-injured athletes	Special populations (e.g., clinical, patients), athletes with a physical or mental disability, and athletes considered to be injured or returning from injury

### Data Extraction

One author (BN) extracted the data using a specifically designed standardised excel spreadsheet. General study information (i.e., author, year), subject characteristics (i.e., sample size, sex, age, body mass, height, sport, training status, performance level), training intervention characteristics (i.e., training methods, control group information, number of sessions per week, duration of training intervention, total amount of training sessions, training intensity, training volume, testing distances, testing equipment, training surface, other training, reported training-related injuries) and primary outcome measures (i.e., pre- and post-training intervention means and standard deviations) were extracted. All studies that included the time or velocity achieved from the initial start position (0 m) to ≤ 5 m, 0 m to between > 5 m to ≤ 10 m and 0 m to between > 10 m to ≤ 20 m were categorised into the 0–5 m, 0–10 m and 0–20 m subgroups respectively. These outcomes aimed to identify training mode specific short-sprint performance changes, whilst representing the typical short-sprint distances performed by football code athletes and those commonly measured by researchers/practitioners.

Descriptive information relating to the training activities performed in the studies was used to categorise each intervention into the training mode subgroups outlined in Table 3. If the pre- and post-outcome measure data was not available from the tables or the result section, the data was requested from the author(s). If the authors did not have access to their data, data on outcome measures were extracted from figures using WebPlotDigitizer version 4.1 software (2018) (Version 4.1, WebPlotDigitizer, USA). Means and SD/SEM were measured manually at the pixel level to the scale provided in the studies figures.

**Table 3:** Subgroup categorisation

*Specific sprint training*—training methods in which the athlete is simulating/performing the sprint movement pattern (see primary and secondary methods)		*Tertiary methods (non-specific sprint training)*—training methods not involving the athlete sprinting, that have a transfer into sprint performance as a result of the subsequent training adaptions (e.g., strength, power, plyometric training). These may be performed individually, (e.g., strength training) or in combination with other tertiary methods (e.g., strength, power and plyometric training)
*Combined specific methods*—training methods that included both primary and secondary methods (e.g., sprinting + resisted sled sprinting)		
*Primary methods*—training methods simulating the sprint movement pattern (sprint-technique drills, stride length and frequency exercises, and sprints of varying distances and intensities)	*Secondary methods*—training methods simulating the sprint action but applying overload by reducing or increasing the speed of the movement by applying additional resistance (e.g., sledges, resistance bands, weighted garments or incline sprints (gravity resisted)) or assistance (e.g., pulley systems, partner assisted or decline sprints (gravity assisted))	
Combined training—training methods that included both specific sprint training (primary and or secondary methods) and tertiary methods in combination (e.g., strength, power, resisted and unresisted sprint training)		
Sport only training—training methods not including any specific or non-specific sprint training. It is described as a format of offensive, defensive and match simulation technical and tactical drills which may include some form of endurance training and or competitive games		

Subgroup categories are based on previous definitions from Plisk et al. (2000) [43] and Rumpf et al. (2016) [44]

### Study Quality Assessment

The study quality assessment of the included studies was assessed using the McMaster [59] scale, which is relevant for sport science research. This scale expands upon the scale created by Brughelli [60] designed to evaluate research conducted in athletic-based training environments from a combination of items from the Cochrane, Delphi and PEDRO. The methodological scale assesses the study in the following 10 domains: inclusion criteria stated, subject assignment, intervention description, control groups, dependent variables definition, assessment methods, study duration, statistics, results section and conclusions. A score of 0 = clearly no; 1 = maybe; and 2 = clearly yes were assigned based on a total study quality assessed from 20.

### Data Analysis and Meta-analyses

Data extracted from the systematic search were included in the meta-analyses. Improvements in sprint performance are typically identified by a reduction in time taken to cover a given distance or an increase in peak velocity achieved for a given time point and or distance [37, 61]. Therefore, pre- and post-time changes were reversed before conducting the analysis so that both time and velocity changes represented the same direction. Thus, identifying a reduction in time or an increase in velocity for a given distance as a positive change.

A random-effects meta-analyses was performed by using Comprehensive Meta-Analysis Version 3.0 software (version 3, Biostat, Englewood, NJ, USA) to assess the magnitude of the outcome across the relevant primary studies and to explore the effect of moderator variables on the variation among study outcomes [62]. Outcome measures were converted into the standardised mean difference (SMD) with 95% confidence intervals (CI) and were used as the summary statistic. The SMD represents the size of the effect of the intervention relative to the variability observed in that intervention. Overall summary estimates were calculated for each of the training type subgroups: primary, secondary, combined specific, tertiary, combined methods and sport only training (Table 3). An inverse-variance random-effects model was used for the meta-analyses because it allocated a proportionate weight to trials based on the size of their individual standard errors and facilitates analysis while controlling for heterogeneity across studies [63]. The inputted data included sample sizes, outcome measures with their respective standard deviations and a correlation coefficient for within-subject measurements. These correlation coefficients (0–5 m *r* = 0.69, 0–10 m *r* = 0.72 and 0–20 m *r* = 0.76) were estimated from prior field testing and other published studies [64–66].

The SMD values were interpreted as < 0.20 as trivial, 0.20–0.39 as small, 0.40–0.80 as moderate and > 0.80 as large [67]. A positive SMD indicates that the training intervention was associated with an improvement in short-sprint performance while a negative SMD indicates that the training intervention was associated with a decrease in the respective performance outcome. Accompanying *p* values tested the null hypothesis that there was no statistically significant change in short-sprint performance regardless of the training method. Statistical significance was considered for *p* < 0.05. Heterogeneity between trials was assessed using the *I*^2^ statistic, moderate (> 50%) to high values (> 75%) were used to indicate for potential heterogeneity sources (Higgins and Thompson 2002) [68]. The *I*^2^ statistic was supported by reporting the Tau-squared statistic and the Chi-squared statistic. Sensitivity analyses were used for each subgroup by repeating the analyses with each study omitted in turn; this would examine whether any conclusions were dependent on a single study.

Subgroup analyses were performed to evaluate the potential moderator variables which were determined a *priori*: sex (male vs female), football code, playing standard (elite vs. sub-elite; [from Swann et al. (2016), the highest reported standard of performance [69]]), age category (senior [mean age ≥ 18 years] vs youth [mean age < 18 years]) and training phase (pre-season vs in-season vs off-season).

### Evaluation of Small Study Effects

Small study effects were evaluated through visual interpretation of funnel plots of SMD versus standard errors and by quantifying Egger's linear regression intercept [70] to evaluate potential bias. A statistically significant Egger statistic (*p* value < 0.05) indicated the presence of a small study effect.

## Results

### Overview

**Fig. 1:**
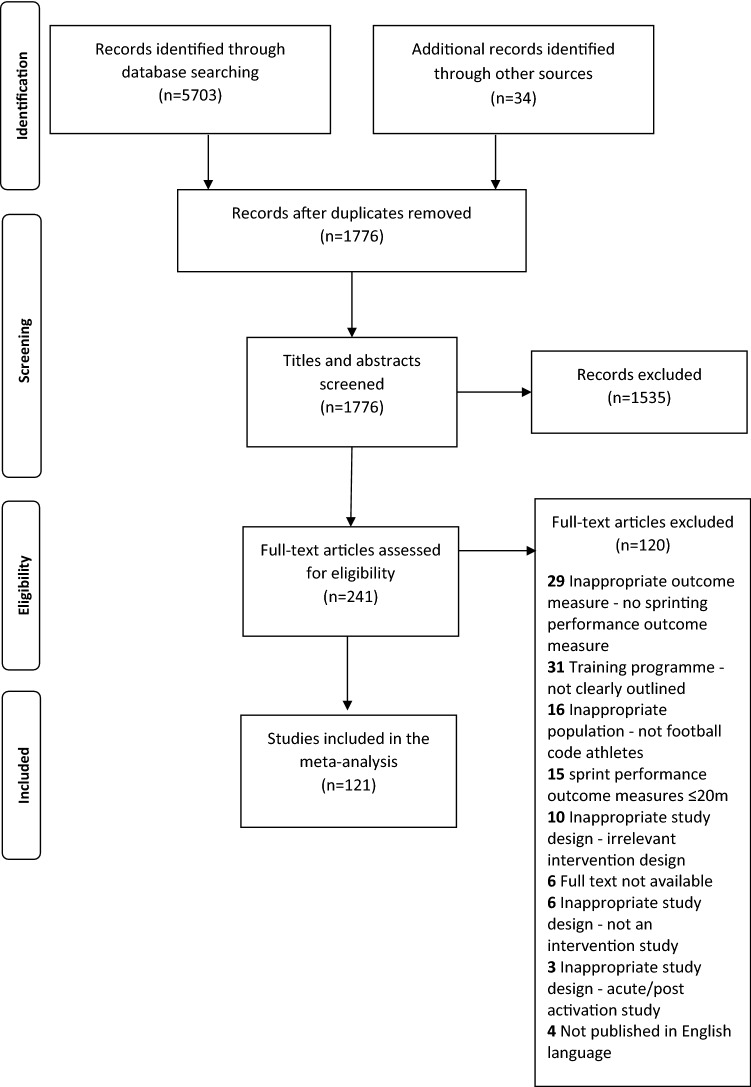
Flow diagram of the process of study selection

Following the removal of duplicates, a total of 1776 studies were found. The study selection inclusion criteria identified 121 studies [40–42, 64–66, 71–181] (Fig. 1). The 121 studies resulted in a total of 220 intervention groups and 64 sport only groups. Training groups were subgrouped into six training classifications (sport only *n* = 64, combined methods *n* = 76, combined specific methods *n* = 3, primary methods *n* = 11 [66, 79, 89, 106, 134, 135, 155, 177], secondary methods *n* = 6 [77, 79, 87, 89, 173] and tertiary methods *n* = 124) to differentiate between findings for distinct short-sprint performance outcomes (Table 3).

Electronic Supplementary Material Table S1 (non-specific/tertiary, *n* = 124), Table S2 (combined, *n* = 76) and Table S3 (specific, *n* = 20) present the individual training groups study descriptives, training intervention and short-sprint outcomes for the included studies. The 121 studies [40–42, 64–66, 71–181] represented a total sample of 3419 football code athletes with a mean sample size of 12.0 ± 5.3 participants per training group. One hundred and ten studies were conducted in males, ten in females [97, 113, 127, 128, 139, 140, 147, 151, 173, 181] and one in mixed populations [106]. The mean age of studies ranged from 10.1 to 26.8 years. The athlete populations ranged from sub-elite to elite [69]. Collectively, the training intervention durations ranged from 2 to 26 weeks (7.6 ± 3.6), with the intervention training frequency ranging between 1 and 4 sessions per week (2.1 ± 0.6) over 6–52 individual sessions.

Studies were conducted in soccer (*n* = 96), rugby league (*n* = 5) [71, 72, 84, 93, 104], rugby union (*n* = 4) [103, 129, 175, 176], futsal (*n* = 3) [113, 159, 171] rugby sevens (*n* = 2) [75, 182], Australian football (*n* = 1) [162] and mixed football codes (*n* = 10) [40, 42, 64, 105, 114–116, 141, 153, 155]. No studies using Gaelic football or American football players satisfied the inclusion criteria. Studies were conducted in pre-season (*n* = 38), in-season (*n* = 64), off-season (*n* = 3) and across pre-season and in-season (*n* = 2). Fourteen studies did not report the phase of the season. Short-sprint assessment distances ranged from 4.6 to 20 m; 0–5 m (*n* = 43), 0–10 m (*n* = 85) and 0–20 m (*n* = 85). Timing devices included electronic timing gate systems (*n* = 98), stopwatches (*n* = 6), velocimeters (*n* = 4), radar measurement devices (*n* = 3), high-speed video cameras (*n* = 3), 1080 sprint devices (*n* = 2), a laser measurement device (*n* = 1), a kinematic measurement system (*n* = 1) and a mobile application (mysprint; *n* = 1). One study failed to report the sprint performance measuring device.

Sport only training groups were generally described as some format of offensive or defensive match simulation and technical and tactical drills 2–6 days per week, 2–11 sessions per week lasting between 30 and 120 min per session as well as some form of endurance training and 1–3 competitive or friendly games/week. There were various methods of endurance training described such as simulated games performed in small-, medium- or large-sided games formats (3 vs 3 to 11 vs 11), low-intensity aerobic conditioning, high-intensity interval training and recreational or cardiovascular activities (basketball, biking, running, aerobics, etc.). Sport only training was conducted in both pre-season and in-season periods over a duration of 6–16 weeks.

Specific sprint training groups performed sprinting, resisted and assisted sprinting and technical sprint drills, completed as individual modalities and or/in combination (e.g., complex and contrast sets). The training was performed 1–3 days per week, with intervention periods lasting from 4 to 12 weeks (6–16 sessions). The primary sprint training methods included single set interventions ranging from 8 to 15 repetitions of short distance (20 m) sprints (160–300 m session totals) or multiple set methods, ranging from 2–5 sets of 2–8 repetitions/set of medium-long distance (25–55 m) sprints (240–680 m session totals). Two studies [66, 106] performed submaximal sprint efforts (90% of best 20 m time), involving 25–30 repetitions of short distance (20 m) sprints (400–600 m session totals). Resisted sprinting was performed as either a single set of 5–10 repetitions of short-sprints (18.3–20 m; 100–200 m session total) or multiple set methods, ranging from 2 to 7 sets of 4 repetitions/set of short-medium distance (5–30 m) resisted sled sprints (130–455 m session total). Resisted sprint loads ranged from light-very heavy loads [52]. Loads were prescribed based on percentage body mass (i.e., 10–80%BM) or reduction in *V*_max_ corresponding with the additional resistance applied (i.e., 10–50% reduction in *V*_max_). One study used partner applied resistance (intensity not specified) for resisted sprints. One study investigated assisted training methods, implementing 1 set of 10 repetitions/set of short-sprints over 18.3 m with a bungee cord assistive load of 14.7%BM (183 m session total; [173]).

Non-specific/tertiary sprint training groups consisted of strength, power and/or plyometrics training performed as individual modalities and or/in combination (e.g., complex and contrast sets). The training was performed 1–5 days per week, with intervention periods lasting from 3 to 16 weeks (6–36 sessions). Lower body strength training (e.g., squat, hip hinge and calf variations) ranged from moderate-supramaximal loads (55–110% 1RM) moderate-high volume training (e.g., 2–6 sets 2–6 repetitions and/ or 2–6 sets of 8–16 repetitions). Power training consisted of ballistic exercises (e.g., squat jump, power clean and snatch) at light to moderate loads (30–75% 1RM) and/or velocity-based training using loads corresponding to the mass at which optimal power is produced (1–1.1 × optimal power load). Exercise volume ranged from 2–5 sets of 2–8 repetitions/set. Plyometrics training involved low–high intensity plyometrics (e.g., ankle hops to 50 cm accentuated eccentric loading drop Jump @ + 20% BM) 1–15 sets of 4–15 repetitions/set (10–260 foot contacts session totals) on various surfaces (grass, dry sand surface, hard and soft surfaces). Several of the sessions were performed in combination with upper body training.

Combined methods training groups consisted of various formats of both specific sprint training (primary and or secondary methods) and tertiary methods in combination (e.g., strength, power, resisted and unresisted sprint training). These were completed as individual modalities and or/ in combination (e.g., complex and contrast sets). The training was performed 1–4 day per week, with intervention periods lasting from 3 to 26 weeks (6–52 sessions). Strength training ranged from moderate-high loads (70–90% 1RM) low–high volume (e.g., 1–8 sets 2–6 repetitions/set and/or 3–4 sets of 8–12 repetitions). Power training consisted of ballistic exercises at light to moderate loads (30–65% 1RM or 15–100%BM) and/or velocity-based training using loads corresponding to the mass at which optimal power is produced (1–1.1 × optimal power load or 80–105% of 1 m·s^−1^ load). Repetition ranges were from 2 to 6 sets of 2 to 8 repetitions per set. This also included medicine ball throws from 3 to 12 kg. Plyometrics sessions involved low–high intensity plyometrics (e.g., ankle hops to 75 cm hurdle jumps) 1–5 sets of 3–15 repetitions per set (9–310 foot contacts session totals). The only type of surface identified was a grass track. The specific sprint training methods included single set interventions ranging from 1 to 5 repetitions short-sprint (5–20 m) or multiple set methods, ranging from 2 to 4 sets of 2 to 8 repetitions/set of medium-long distance sprints (25–40 m) (80–340 m session totals) from various starting positions. Resisted sprint loads ranged from light-very heavy loads [52]. Loads were prescribed based on percentage body mass (i.e., 5–55%BM), absolute loads (i.e., 5–10 kg) or reduction in *V*_max_ corresponding with the additional resistance applied (i.e., 10% reduction in *V*_max_). A number of the sessions were performed in combination with upper body training.

### Study Quality

The scores for the assessment of study quality according to McMaster et al. (2013) [59] are shown in Table 4, ranging from 14 to 20 with a mean score of 18.1 ± 1.7 demonstrating high study quality. Items 2 (subjects assigned appropriately (random/equal baseline)), 4 (control group inclusion), 6 (assessments practical) and 9 (results detailed (mean, SD, percent change, effect size)) were the most decisive factors in separating the high-quality from the low-quality studies.

**Table 4:** Methodological quality scale scores

Study	Question number										Score
	1	2	3	4	5	6	7	8	9	10	
Alves et al. (2010) [73]	2	2	2	2	2	2	2	2	0	2	18
Arcos et al. (2014) [118]	2	2	2	0	2	2	2	2	2	2	18
Asadi et al. (2018) [74]	2	2	2	2	2	2	2	2	2	2	20
Barr et al. (2015) [75]	2	2	2	2	2	2	2	2	2	2	20
Beato et al. (2018) [76]	2	2	2	0	2	2	2	2	2	2	18
Bianchi et al. (2019) [183]	2	2	2	0	2	2	2	2	2	2	18
Borges et al. (2016) [77]	2	2	2	0	2	2	2	2	0	2	16
Bouguezzi et al. (2018) [78]	2	2	2	0	2	2	2	2	2	2	18
Bremec (2018) [79]	2	2	2	2	2	2	2	2	2	2	20
Brito et al. (2014) [80]	2	2	2	2	2	2	2	2	2	2	20
Cavaco et al. (2014) [81]	2	2	2	2	2	2	2	2	0	0	16
Chelly et al. (2010) [82]	2	2	2	2	2	2	2	2	2	2	20
Christou et al. (2006) [83]	2	2	2	2	2	2	2	2	2	2	20
Comfort et al. (2012) [84]	2	0	2	0	2	2	2	2	0	2	14
Coratella et al. (2019) [85]	2	2	2	2	2	2	2	2	2	2	20
Corrêa et al. (2016) [184]	2	0	2	0	2	2	2	2	0	2	14
Coutts et al. (2004) [72]	2	2	2	0	2	2	2	2	0	2	16
Coutts et al. (2007) [71]	2	0	2	0	2	2	2	2	0	2	14
De Hoyo et al. (2015) [86]	2	0	2	2	2	2	2	2	0	2	16
De Hoyo et al. (2016) [87]	2	2	2	0	2	2	2	2	2	2	18
De Villarreal et al. (2015) [88]	2	2	2	2	2	2	2	2	2	2	20
Derakhti et al. (2018) [89]	2	2	2	2	2	2	2	2	2	2	20
Douglas et al. (2018) [40]	2	2	2	2	2	2	2	2	2	2	20
Enoksen et al. (2013) [90]	2	2	2	2	2	2	2	2	2	2	20
Faude et al. (2013) [91]	2	2	2	2	2	2	2	2	2	2	20
Franco-Márquez et al. (2015) [92]	2	2	2	2	2	2	2	2	2	2	20
Gabbett et al. (2008) [93]	2	0	2	0	2	2	2	2	1	2	15
Garci´a-Pinillos et al. (2014) [94]	2	2	2	2	2	2	2	2	0	2	18
Gil et al. (2018) [95]	2	2	2	0	2	2	2	2	2	2	18
González-Badillo et al. (2015) [96]	2	0	2	2	0	2	2	2	0	2	14
González-García et al. (2019) [97]	2	2	2	2	2	2	2	2	1	2	19
Gorostiaga et al. (2004) [98]	2	2	2	2	2	2	2	2	0	2	18
Griffiths et al. (2019) [99]	2	2	2	0	2	2	2	2	2	2	18
Hammami et al. (2016) 1 [101]	2	2	2	2	2	2	2	2	0	2	18
Hammami et al. (2016) 2 [102]	2	2	2	0	2	2	2	2	2	2	18
Hammami et al. (2018) [100]	2	2	2	2	2	2	2	2	2	2	20
Harries et al. (2017) [103]	2	2	2	2	2	2	2	2	2	2	20
Harris et al. (2008) [104]	2	2	2	0	2	0	2	2	0	2	14
Harrison and Bourke (2009) [105]	2	2	2	2	2	2	2	2	0	2	18
Haugen et al. (2014) [106]	2	2	2	2	2	2	2	2	0	2	18
Haugen et al. (2015) [66]	2	2	2	2	2	2	2	2	0	2	18
Helgerud et al. (2011) [185]	2	0	2	0	2	2	2	2	0	2	14
Impellizzeri et al. (2007) [107]	2	2	2	0	2	2	2	2	2	2	18
Ishøi et al. (2017) [108]	2	2	2	0	2	2	2	2	2	2	18
Kobal et al. (2017) 1 [109]	2	2	2	0	2	2	2	2	0	2	16
Kobal et al. (2017) 2 [110]	2	2	2	0	2	2	2	2	2	2	18
Koundourakis et al. (2014) [111]	2	0	1	2	2	2	2	2	0	2	15
Krommes et al. (2017) [112]	2	2	2	2	2	2	2	2	2	2	20
Lago-Fuentes et al. (2018) [113]	2	2	2	0	2	2	2	2	1	2	17
Lockie et al. (2012) 1 [115]	2	2	2	0	2	2	2	2	2	2	18
Lockie et al. (2012) 2 [114]	2	2	2	0	2	2	2	2	2	2	18
Lockie et al. (2014) [116]	2	2	2	0	2	2	2	2	2	2	18
Lopez-Segovia et al. (2010) [117]	2	2	2	2	2	2	2	2	2	2	20
Loturco et al. (2013) [119]	2	2	2	0	2	2	2	2	0	2	16
Loturco et al. (2015) 1 [120]	2	2	2	0	2	2	2	2	2	2	18
Loturco et al. (2015) 2 [121]	2	2	2	0	2	2	2	2	2	2	18
Loturco et al. (2015) 3 [122]	2	2	2	0	2	2	2	2	2	2	18
Loturco et al. (2016) 1 [123]	2	2	2	0	2	2	2	2	2	2	18
Loturco et al. (2016) 2 [41]	2	2	2	0	2	2	2	2	0	2	16
Loturco et al. (2017) [124]	2	2	2	0	2	2	2	2	0	2	16
Loturco et al. (2019) [186]	2	2	2	0	2	2	2	2	2	2	18
Manolopoulos et al. (2004) [125]	2	2	2	2	2	2	2	2	0	2	18
Manouras et al. (2016) [126]	2	2	2	2	2	2	2	2	0	2	18
Marques et al. (2019) [159]	2	2	2	2	2	2	2	2	2	2	20
Mathisen and Danielsen (2014) [127]	2	2	1	2	2	2	2	2	0	2	17
Mathisen and Pettersen (2015) [128]	2	2	1	2	2	2	2	2	0	2	17
McMaster et al. (2014) [129]	2	2	2	0	2	2	2	2	2	2	18
McMorrow et al. (2019) [130]	2	2	2	0	2	2	2	2	2	2	18
Mendiguchia et al. (2015) [131]	2	2	2	2	2	2	2	2	2	2	20
Meylan and Malatesta (2009) [132]	2	2	2	2	2	2	2	2	0	2	18
Michailidis et al. (2019) [133]	2	2	2	2	2	2	2	2	0	2	18
Morin et al. (2017) [134]	2	2	2	2	2	2	2	2	2	2	20
Mujika et al. (2009) [135]	2	2	2	0	2	2	2	2	0	2	16
Nakamura et al. (2012) [136]	2	0	2	2	2	2	2	2	2	2	18
Negra et al. (2016) [137]	2	2	2	2	2	2	2	2	0	2	18
Negra et al. (2019) [138]	2	2	2	0	2	2	2	2	2	2	18
Nonnato et al. (2020) [181]	2	2	2	2	2	2	2	2	2	2	20
Ozbar (2015) [139]	2	2	2	2	2	2	2	2	2	2	20
Ozbar et al. (2014) [140]	2	2	2	2	2	2	2	2	0	2	18
Pienaar and Coetzee (2013) [141]	2	2	2	2	2	2	2	2	2	2	20
Ramirez-Campillo et al. (2013) [142]	2	2	2	2	2	2	2	2	1	2	19
Ramirez-Campillo et al. (2014) 1 [143]	2	2	2	2	2	2	2	2	2	2	20
Ramirez-Campillo et al. (2014) 2 [144]	2	2	1	2	2	2	2	2	1	2	18
Ramirez-Campillo et al. (2015) 1 [146]	2	2	2	2	2	2	2	2	2	2	20
Ramirez-Campillo et al. (2015) 2 [145]	2	2	2	2	2	2	2	2	2	2	20
Ramirez-Campillo et al. (2016) [147]	2	2	2	2	2	2	2	2	0	2	18
Ramirez-Campillo et al. (2018) 1 [150]	2	2	2	2	2	2	2	2	2	2	20
Ramirez-Campillo et al. (2018) 2 [149]	2	2	2	2	2	2	2	2	2	2	20
Ramirez-Campillo et al. (2018) 3 [148]	2	2	2	2	2	2	2	2	1	2	19
Ramirez-Campillo et al. (2018) 4 [151]	2	2	2	2	2	2	2	2	1	2	19
Ramirez-Campillo et al. (2019) [152]	2	2	2	2	2	2	2	2	2	2	20
Randell et al. (2011) [153]	2	2	2	2	2	2	2	2	2	2	20
Rey et al. (2017) [154]	2	2	2	2	2	2	2	2	2	2	20
Rimmer and Sleivert (2000) [155]	2	2	2	2	2	2	2	2	0	2	18
Rodriguez-Rosell et al. (2016) [156]	2	2	2	2	2	2	2	2	2	2	20
Rodriguez-Rosell et al. (2017) 1 [157]	2	2	2	2	2	2	2	2	2	2	20
Rodriguez-Rosell et al. (2017) 2 [158]	2	2	2	2	2	2	2	2	2	2	20
Ronnestad et al. (2008) [160]	2	2	2	2	2	2	2	2	0	2	18
Ross et al. (2015) [182]	2	2	2	0	2	2	2	2	2	2	18
Sánchez-Sánchez et al. (2015) [161]	2	2	2	2	2	2	2	2	0	2	18
Scott et al. (2017) [162]	2	2	2	2	2	2	2	2	0	2	18
Seitz (2015) [164]	2	2	2	0	2	2	2	2	2	2	18
Shalfawi et al. (2012) [163]	2	2	2	2	2	2	2	2	2	2	20
Singh et al. (2014) [165]	2	0	2	2	2	0	2	2	0	0	12
Söhnlein et al. (2014) [166]	2	0	2	2	2	2	2	2	2	2	18
Spinks et al. (2007) [42]	2	2	2	2	2	2	2	2	0	2	18
Styles et al. (2016) [167]	2	0	2	0	2	2	2	2	2	2	16
Suarez-Arrones et al. (2019) [168]	2	2	2	2	2	2	2	2	2	2	20
Thomas et al. (2009) [169]	2	2	2	0	2	2	2	2	0	2	16
Tønnessen et al. (2011) [170]	2	2	2	2	2	2	2	2	2	2	20
Torres-Torrelo et al. (2017) [171]	2	2	2	2	2	2	2	2	2	2	20
Tous-Fajardo et al. (2016) [172]	2	0	2	2	2	2	2	2	0	2	16
Upton (2011) [173]	2	2	2	0	2	2	2	2	0	2	16
Venturelli et al. (2008) [177]	2	2	2	0	2	2	2	2	0	2	16
Vera-Assaoka et al. (2019) [174]	2	2	2	2	2	2	2	2	2	2	20
Weakley et al. (2019) [175]	2	2	2	2	2	2	2	2	2	2	20
West et al. (2013) [176]	2	2	2	0	2	2	2	2	0	2	16
Winwood et al. (2015) [64]	2	2	2	0	2	2	2	2	0	2	16
Wong et al. (2010) [178]	2	0	2	2	2	2	2	2	0	2	16
Yanci et al. (2016) [179]	2	2	2	0	2	2	2	2	2	2	18
Zghal et al. (2019) [180]	2	2	2	2	2	2	2	2	2	2	20

0 = clear no; 1 = maybe; and 2 = clearly yes

### Meta-analysis

Individual study statistics can be seen in the Electronic Supplementary Material Tables S1–S3.

### Standardised Mean Difference for 0–5 m Performance

**Fig. 2:**
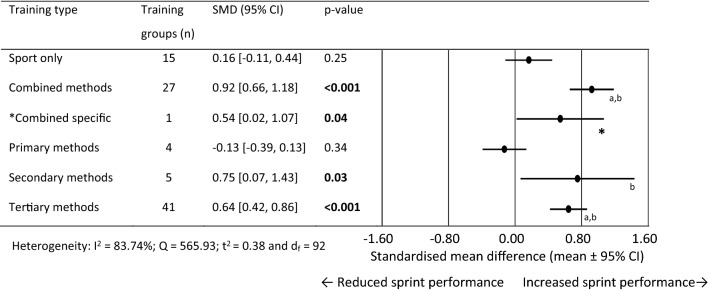
Forest plots showing the standardised mean differences (mean ± 95% CI) for the studies evaluating the between training group effects on 0–5 m sprint performance. Bold font = *p* < 0.05 and *less than 3 studies in this training group. ^a^Significantly different to sport only training *p* < 0.05, ^b^Significantly different to primary methods training methods *p* < 0.05. *SMD* standardised mean differences and *CI* confidence interval

For 0–5 m performance, 93 training group effects were analysed from 43 original studies [41, 42, 64, 73, 77–80, 82, 88, 89, 94, 95, 98, 100, 101, 105, 110, 112, 114–116, 120, 122–124, 130, 131, 134, 136–138, 141, 166–169, 173, 179, 180, 183–185]. Figure 2 shows the SMD for each training type. The sport only and primary methods training failed to show statistical significance for change in 0–5 m performance. Combined methods showed large improvements, while combined specific, secondary and tertiary methods showed moderate performance improvements. The combined and tertiary methods were the only training methods that produced a significantly larger training effect than sport only training. All methods produced a significantly larger training effect than the primary methods, apart from the sport only group. Between-subgroup analysis was not conducted on the combined specific subgroup as only one training group was available.

### Standardised Mean Difference for 0–10 m Performance

**Fig. 3:**
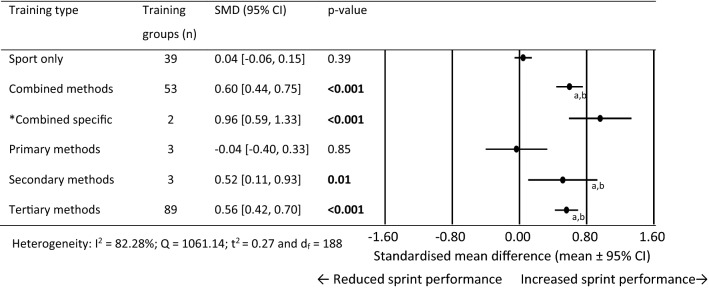
Forest plots showing the standardised mean differences (mean ± 95% CI) for the studies evaluating the between training group effects on 0–10 m sprint performance. Bold font = *p* < 0.05 and *less than 3 studies in this training group. ^a^Significantly different to sport only training *p* < 0.05, ^b^Significantly different to primary methods training methods *p* < 0.05. *SMD* standardised mean differences and *CI* confidence interval

For 0–10 m performance, 189 training group effects were analysed from 85 original studies [40–42, 65, 71, 72, 75, 76, 78, 79, 83–95, 97, 99, 101–105, 107–117, 119–130, 132, 136–139, 141, 153–162, 165–169, 171, 172, 175, 176, 178, 180–182, 185, 186]. Figure 3 shows the SMD for each training type. The sport only training and primary methods failed to show statistical significance for change in 0–10 m performance. Combined specific methods showed large, while combined, secondary and tertiary methods showed moderate performance improvement. The combined, secondary and tertiary methods produced a significantly larger training effect than sport only and primary training methods. Between-subgroup analysis was not conducted on the combined specific subgroup as only two training groups where available.

### Standardised Mean Difference for 0–20 m Performance

**Fig. 4:**
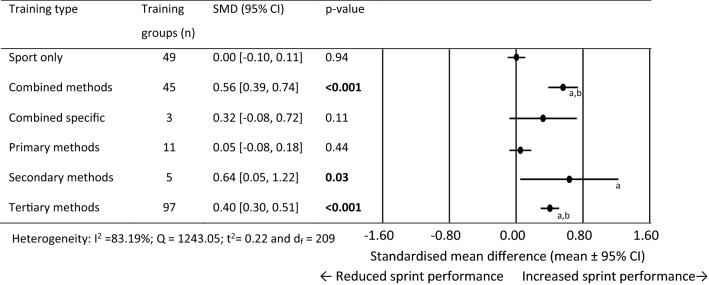
Forest plots showing the standardised mean differences (mean ± 95% CI) for the studies evaluating the between training group effects on 0–20 m sprint performance. ^a^Significantly different to sport only training *p* < 0.05, ^b^Significantly different to primary methods training methods *p* < 0.05. Bold font = *p* < 0.05; *SMD* standardised mean differences and *CI* confidence interval

For 0–20 m performance, 210 training group effects were analysed from 85 original studies [40–42, 64–66, 72–74, 78–81, 84, 86, 87, 89, 92–98, 101, 103, 106, 107, 109–111, 117, 118, 120–124, 127–131, 134–143, 145–153, 155–158, 161–164, 166–171, 173–175, 177, 179, 180, 184, 185, 187] Fig. 4 shows the SMD for each training type. The sport only training, combined specific and primary methods training failed to show statistical significance for change in 0–20 m performance. Combined, secondary and tertiary methods showed moderate performance improvement. Combined and tertiary methods produced significantly larger training effects than sport only and primary methods training groups. The secondary training methods produced a significantly larger training effect than sport only training.

### Heterogeneity

The degree of overall heterogeneity was high for all outcome measures between studies *I*^2^ (> 75%).

### Sensitivity

Sensitivity analysis revealed minor changes only, for the majority of performance variables, and these changes did not substantially impact the overall mean effect. Combined specific and secondary training methods were sensitive to the exclusion of one or more studies independently and in turn, moderated the statistical interpretation of the results. Removal of one of the three combined specific 0–20 m studies [127] moderated the statistical significance from non-significant (*p* > 0.05) to significant (*p* < 0.05). Removal of three of the four 0–5 m and 0–20 m studies [79, 89, 173] and two of the three 0–10 m studies [79, 89, 173] moderated the statistical significance from significant to non-significant.

### Evaluation of Small Study Effects

Inspection of the funnel plot and Egger’s regression intercept revealed a large and statistically significant Egger statistic indicating that there was evidence of small study effects for the 0–5 m (intercept 5.23, 95% CI 3.51–6.95; *p* < 0.001), 0–10 m (intercept 4.61, 95% CI 3.32–5.91; *p* < 0.001) and 0–20 m (intercept 4.66, 95% CI 3.58–5.74; *p* < 0.001). The SMD between pre- and post-intervention sprint performance was not considered symmetrical, suggesting the presence of significant publication bias [188].

### Moderator Variables

Table 5 presents the subgroup analysis assessing potential moderating factors for short-sprint performance (0–5 m, 0–10 m and 0–20 m). Regarding the population characteristics, between-subgroup analysis significant (*p* < 0.05) identified that football code, age and phase of training all moderated overall magnitude of training effects. There was no statistical difference between sex and playing standard groups.

**Table 5:** Summary of moderator variable analysis for football code, sex and playing standard age and phase of training meta-analysis by subgroup with the sport only training groups removed

Moderator variable	Between group differences	Subgroup standardised mean difference
Football code	**0–5 m** Soccer vs ^a^Rugby league **0–10 m** Soccer vs Rugby union *p* = 0.14 Soccer vs Futsal *p* = 0.88 Soccer vs Rugby league *p* = 0.10 Soccer vs Rugby sevens ***p*** **< 0.001** Rugby union vs Futsal *p* = 0.36 Rugby union vs Rugby league *p* = 0.36 Rugby union vs Rugby sevens ***p*** **< 0.001** Futsal vs Rugby league *p* = 0.15 Futsal vs Rugby sevens ***p*** **< 0.001** Rugby league vs Rugby sevens *p* = 0.06 ^a^Australian rules football **0–20 m** Soccer vs Rugby union *p* = 0.72 Soccer vs Rugby league *p* = 0.59 Rugby union vs Rugby league *p* = 0.69 ^a^Futsal ^a^Australian rules football	**Soccer** 0–5 m (*n* = 61; SMD = 0.66; 95% CI [0.47, 0.85]; ***p*** **< 0.001**) 0–10 m (*n* = 99; SMD = 0.65; 95% CI [0.52, 0.78]; ***p*** **< 0.001**) 0–20 m (*n* = 128; SMD = 0.46; 95% CI [0.37, 0.56]; ***p*** **< 0.001**)
		**Rugby union** 0–5 m (N/A) 0–10 m (*n* = 8; SMD = 0.48; 95% CI [0.31, 0.66]; ***p*** **< 0.001**) 0–20 m (*n* = 6; SMD = 0.43; 95% CI [0.24, 0.61]; ***p*** **< 0.001**)
		**Futsal** 0–5 m (N/A) 0–10 m (*n* = 5; SMD = 0.68; 95% CI [0.30, 1.06]; ***p*** **< 0.001**) ^a^0–20 m (*n* = 2; SMD = 0.34; 95% CI [0.05, 0.63] ***p*** **= 0.02**)
		**Rugby league** ^a^0–5 m (*n* = 1; SMD = 1.19; 95% CI [0.72, 1.65]; ***p*** **< 0.001**) 0–10 m (*n* = 10; SMD = 0.27; 95% CI [− 0.16, 0.69]; *p* = 0.22) 0–20 m (*n* = 9; SMD = 0.31; 95% CI [− 0.20, 0.83]; *p* = 0.24))
		**Rugby sevens** 0–5 m (N/A) 0–10 m (*n* = 4; SMD = − 0.23; 95% CI [− 0.52, 0.07]; *p* = 0.14) 0–20 m (N/A)
		**Australian rules football** 0–5 m (N/A) ^a^0–10 m (*n* = 2; SMD = − 0.59; 95% CI [− 0.96, − 0.21]; ***p*** **< 0.001**) ^a^0–20 m (*n* = 2; SMD = − 0.27; 95% CI [− 0.59, 0.06]; *p* = 0.11)
Sex	**0–5 m** Male vs female *p* = 0.07 **0–10 m** Male vs female *p* = 0.23 **0–20 m** Male vs female *p* = 0.06	**Male** 0–5 m (*n* = 75; SMD = 0.70; 95% CI [0.54, 0.86]) ***p*** **< 0.001** 0–10 m (*n* = 142; SMD = 0.55; 95% CI [0.45, 0.65]) ***p*** **< 0.001** 0–20 m (*n* = 148; SMD = 0.40; 95% CI [0.31, 0.49]) ***p*** **< 0.001**
		**Female** 0–5 m (*n* = 3; SMD = 0.88; 95% CI [− 0.40, 2.15]) *p* = 0.18 0–10 m (*n* = 8; SMD = 0.85; 95% CI [0.36, 1.34]; ***p*** **< 0.01**) 0–20 m (*n* = 12; SMD = 0.84; 95% CI [0.40, 1.28]; ***p*** **< 0.001**
Playing standard	**0–5 m** Elite vs sub-elite ***p*** **= 0.04** **0–10 m** (Elite vs sub-elite) *p* = 0.23 **0–20 m** Elite vs sub-elite *p* = 0.08	**Elite** 0–5 m (*n* = 50; SMD = 0.65; 95% CI [0.44, 0.85]; ***p*** **< 0.001**) 0–10 m (*n* = 104; SMD = 0.56; 95% CI [0.43, 0.68]; ***p*** **< 0.001** 0–20 m (*n* = 109; SMD = 0.40; 95% CI [0.30, 0.51]; ***p*** **< 0.001**)
		**Sub elite** 0–5 m (*n* = 17; SMD = 1.09; 95% CI [0.72, 1.47]; ***p*** **< 0.001**) 0–10 m (*n* = 32; SMD = 0.69; 95% CI [0.51, 0.86]; ***p*** **< 0.001**) 0–20 m (*n* = 31; SMD = 0.63; 95% CI [0.40, 0.86]; ***p*** **< 0.001**)
Age	**0–5 m** Senior vs youth *p* = 0.06 **0–10 m** Senior vs youth ***p*** **= 0.02** **0–20 m** Senior vs youth ***p*** **< 0.001**	**Senior** 0–5 m (*n* = 52; SMD = 0.82; 95% CI [0.62, 1.03]; ***p*** **< 0.001**) 0–10 m (*n* = 98; SMD = 0.66; 95% CI [0.53, 0.79]; ***p*** **< 0.001**) 0–20 m (*n* = 79; SMD = 0.60; 95% CI [0.45, 0.75]; ***p*** **< 0.001**)
		**Youth** 0–5 m (*n* = 24; SMD = 0.51; 95% CI [0.26, 0.77]; ***p*** **< 0.001**) 0–10 m (*n* = 49; SMD = 0.42; 95% CI [0.27, 0.58]; ***p*** **< 0.001**) 0–20 m (*n* = 80; SMD = 0.30; 95% CI [0.20, 0.39]; ***p*** **< 0.001**)
Phase	**0–5 m** In-season vs off-season ***p*** **= 0.01** In-season vs pre-season *p* = 0.85 Pre-season vs off-season ***p*** **= 0.01** **0–10 m** In-season vs off-season *p* = 0.26 In-season vs pre-season *p* = 0.60 Pre-season vs off-season *p* = 0.32 **0–20 m** In-season vs off-season ***p*** **= 0.03** In-season vs pre-season *p* = 0.88 Pre-season vs off-season ***p*** **= 0.03**	**In-season** 0–5 m (*n* = 44; SMD = 0.78; 95% CI [0.59, 0.96]; ***p*** **< 0.001**) 0–10 m (*n* = 77; SMD = 0.60; 95% CI [0.48, 0.73]; ***p*** **< 0.001**) 0–20 m (*n* = 75; SMD = 0.41; 95% CI [0.29, 0.53]; ***p*** **< 0.001**)
		**Off-season** 0–5 m (*n* = 3; SMD = − 0.16; 95% CI [− 0.83, 0.51]; *p* = 0.64) 0–10 m (*n* = 3; SMD = 0.01; 95% CI [− 1.02, 1.04] *p* = 0.99) 0–20 m (*n* = 3; SMD = − 0.18; 95% CI [− 0.7, 0.34]; *p* = 0.50)
		**Pre-season** 0–5 m (*n* = 25; SMD = 0.78; 95% CI [0.45, 1.11]; ***p*** **< 0.001**) 0–10 m (*n* = 54; SMD = 0.54; 95% CI [0.35, 0.73]; ***p*** **< 0.001**) 0–20 m (*n* = 54; SMD = 0.42; 95% CI [0.26, 0.59]; ***p*** **< 0.001**)

Subgroup analyses were performed on SMD between post and pre-intervention sprint performance outcome. Some studies were not included because the value used for subgroup analysis was not reported or did not match the appropriate categories

Bold font = *p* < 0.05

*N/A* data not available, *SMD* standardised mean differences and *CI* confidence interval

^a^Less than three studies in this training group

## Discussion

### Overview of the Main Findings

Multiple training methods are recommended for improving short-sprint performance due to its importance in the football codes [39, 43, 46, 48, 50–57]. This review is the first to (1) systematically review the scientific literature evaluating training interventions upon short-sprint performance (0–5 m, 0–10 m and 0–20 m) in football code athletes, (2) undertake a meta-analysis to assess the magnitude of change in short-sprint performance following training interventions and (3) identify how moderator variables (i.e., football code, sex, playing standard, age and phase of season) affect the training response. The review analysed 121 studies [40–42, 64–66, 71–181], totalling 3419 athletes short-sprint performance providing the largest systematic evidence base for enhancing short-sprint performance in football code athletes (i.e., the previous largest sample is 48 studies from a mixed sample) [39, 43, 46, 48, 50–57, 59].

In summary, the meta-analysis showed that short-sprint performance can be enhanced through combined, secondary and tertiary training methods. Combined specific training methods also showed moderate significant improvements in short-sprint performance at 0–5 m and 0–10 m. These findings support previous literature stating that football code athletes sprint performance can be enhanced concurrently alongside football code specific training [39]. Sport only training and primary training methods showed no effect on short-sprint performance, suggesting such training alone is insufficient to improve short-sprint performance. Between-subgroup analysis identified that football code, age, playing standard and phase of training all moderated overall magnitude of training effects. Athlete’s sex demonstrated no significant difference between-subgroups.

### Summary of Interventions to Develop Short-Sprint Performance

The included 121 studies were categorised into six training mode categories resulting in 286 training groups (i.e., sport only, *n* = 64; combined methods, *n* = 76; combined specific methods, *n* = 3; primary methods, *n* = 11, secondary methods, *n* = 6; and tertiary methods, *n* = 124). This highlights the volume of tertiary method training studies and the reported gap in the available literature to support specific sprint training methods (primary, secondary and combined specific training methods) in football code athletes [48, 52]. The scarcity of specific sprint training method studies maybe because in practice, football code coaches typically implement tertiary training methods to develop multiple physical qualities (i.e., resistance training and plyometrics [16, 189, 190]). This is also a strength of the current systematic review as previous reviews [43, 48] have not considered all training undertaken by the intervention groups within their analysis (e.g., primary or secondary training groups also completing tertiary training methods [42, 105, 114–116, 176]).

The degree of overall heterogeneity was high for all outcome measures between studies (*I*^2^ > 75% [191]. Heterogeneity in systematic reviews is to be expected due to grouping studies together that are diverse, both clinically and methodologically [191]. The high degree of heterogeneity reflects the diversity of the training effects presented. This is likely due to wide variation in the intervention characteristics, including training principles (e.g., frequency [73], duration [75, 96], intensity [66, 192], volume [111], other training completed), population characteristics (e.g., sex [106, 193], chronological age [157], maturation [45], code [42], playing standard [40, 97], playing position, baseline physical characteristics [182] and training experience [168]) and performance monitoring methodology (e.g., equipment [99, 105], start position [194], environmental factors [37], testing frequency and re-test time point [195, 196]). Therefore, although the supplementary tables (Electronic Supplementary Material Tables S1–S3) provide a summary of training methods for all 121 papers included in this meta-analysis, caution is warranted when interpreting the findings of this review and their implications for practice as the variation of the effect sizes demonstrates that training response is highly individualised.

The study quality assessment demonstrated high study quality (18.1 ± 1.7, ranging from 14 to 20). A methodological study scale was used to evaluate research conducted in athletic-based training environments [59], showing that to increase the quality of future studies researchers should randomise participants, include a control group, ensure appropriate testing equipment is used and provide a detailed results section. Although difficult in elite applied environments, to improve the quality of future studies, investigators should allocate subjects to training groups randomly or through group equalisation, include a control group, ensure appropriate testing equipment is used and provide a detailed results section for enhancing study quality. Most interventions were conducted within applied settings; however, several studies failed to describe the additional training or provided limited information. It is important to include any concurrent training stimuli to fully assess if there were any outside interactions with any adaptations seen following a training intervention [197].

Most of the training interventions have reported positive effects on sprinting capabilities leading to the assumption that sprinting performance is easily improved with a variety of methods. However, this needs to be considered from the context of the literature base. Included studies represented both youth and senior athletes from elite and sub-elite cohorts with the majority having limited previous systematic exposure to the intervention methods (e.g., [66, 74, 79, 87, 88, 92, 93, 96–98, 109, 198]). Based on the dose–response relationship and the diminishing returns principle, athletes with a relatively low training age are more likely to have greater training response [199–201]. Observations of elite athletes over time show that mean annual within-athlete performance differences (0.1–0.2%) are lower than typical variation, or smallest worthwhile change and the influence of external conditions (e.g., wind, temperature, altitude, timing methods/procedures) [37, 201]. Furthermore, an inspection of the funnel plot and Egger's regression intercept identified evidence of small-study effects for all performance outcomes. SMD between pre- and post-intervention short-sprint outcomes were not considered symmetrical, suggesting the presence of significant publication bias. While publication bias towards studies reporting positive outcomes may be involved, another plausible explanation is the lack of a control group in many studies, as the results might have been affected by learning effects or the football code training in the intervention period.

### Subgroup Analyses of Training Methods

Training intervention subgroups (i.e., sport only, primary, secondary, tertiary, combined and combined-specific) were categorised based on the principle of specificity. Primary methods were the most specific to simulating the sprint movement pattern [202], and secondary methods were less specific as they overloaded the sprinting action. The tertiary training methods included strength, power and plyometric training and were, therefore, considered the least ‘specific’ to short-sprint performance. Instead of simulating the movement, these methods are commonly performed to target neuromuscular adaptations associated with increased hip, knee and ankle joint movements and the resultant magnitude of ground reaction forces generated during sprinting [203]. The extent to which the method impacts and ‘transfers’ to short-sprint performance ultimately determines the quality of a training programme and the exercises in the context of improving athletic performance [204].

The magnitude and orientation of ground reaction forces are the largest determinants of maximal running speed in humans [205–211]. Short-sprint performance is underpinned by an athlete’s ability to generate the largest net horizontal force possible, averaged across each step [209, 212]. Therefore, the mechanical pretence of the included methodologies, work based on increasing impulse (net forces applied over time) and mechanical efficacy (forces applied in a horizontal direction as velocity increases) [212]. Effective training methods improve either or both the athletes’ capacity to generate high levels of force relative to their body mass, while progressively reducing the contact time of each step and the mechanical effectiveness to transmit the force to the ground at progressively greater running velocities [205, 207, 209, 211, 213–215]. These improvements have been described to manifest in specific transferable training adaptations typically categorised as neural or morphological (architectural or structural) factors [216, 217]. Examples of neural adaptations are increased neural drive and activation, increased motor unit recruitment, increased rate coding and improved intra-muscular coordination (e.g., diversification of sensorimotor solutions; reduced antagonistic activation, increased synergistic activation and stiffness in highly similar patterns of recruitment to the target task [26, 212, 216–218]). Morphological adaptations can be categorised as either architectural adaptations (e.g., increased cross-sectional area, changes in pennation angle, fascicle length and stiffness [tendon and passive elements] [26, 195, 212, 216–218]) or structural adaptations (e.g., fibre type transitions, the collagen content of extracellular matrix and the shift in expression of myosin heavy chain isoforms [26, 212, 216–218]). Practitioners exercise selection is typically based upon the principle of specificity, with a greater training transfer between the mechanical and physiological characteristics of the resistance training exercises and those of the performance movements enhancing the transfer of adaptations [21, 26, 44, 217]. Therefore, practitioners should also consider the shifting mechanical and neuromuscular (e.g., hip, knee, ankle flexors and extensors) requirements the change across the sub-phases (early-, mid- and late acceleration) of a short-sprint [26, 219–221].

#### Sport Only Training

Sport only training is the inclusion of football code training separate of any specific or non-specific sprint training; often undertaken to develop technical and tactical performance within football.

Sport only training (64 studies across football code athletes) was insufficient to significantly improve short-sprint performance at any distance [42, 66, 73, 74, 79–81, 83, 85, 86, 88–92, 94, 97, 100, 101, 103, 106, 108, 112, 117, 125–128, 131, 132, 137, 139, 140, 142–152, 155–161, 165, 166, 168, 170, 171, 174, 178, 180]. However, there was no negative effect on short-sprint performance. Although the movement demands of training are typically below game demands, the football codes are characterised by multidirectional and intermittent bouts of high-intensity running and sprinting interspersed between bouts of moderate and low-intensity activity (e.g., jogging, walking and repositioning [222–225]). Although during training and match play football code athletes repeatedly perform short-sprints (e.g., 5–20 m, 2–3 s) during and in between sport-specific actions [12, 222, 223, 226, 227] athletes are exposed to limited or no very high-speed or sprint threshold running [224, 225, 228] and incomplete rests between sprints. Recommendations for improving sprint performance state that sprints should be at 95–100% max effort with complete recovery [201]. Research has demonstrated that residual fatigue reduces an athlete’s ability to generate force–power–velocity and mechanical efficacy during games and in response, incomplete recovery between sprints [229, 230]. Given the exponential relationship between power and velocity, a reduction from the maximal intensity of the short-sprints would represent a substantial reduction in force and power load on the neuromuscular system providing an insufficient stimulus for adaptation [201]. At a biochemical level, the sport only training movement demands presents a conflicting stimulus, potentially eliciting an interference effect on the development of maximal force and power [231]. The interference effect will then likely affect the resultant magnitude of ground reaction forces the athlete can generate, preventing an athlete from developing short-sprint performance [231]. Therefore, evidence suggests that sport only training alone is insufficient to improve short-sprint performance outcomes. Practitioners should be aware of this within their planning and delivery of training within football code athletes.

#### Primary Methods

Primary methods (*n* = 8) simulate the sprint movement pattern (e.g., sprint-technique drills, stride length and frequency exercises and sprints of varying distances and intensities). The current findings suggest that primary training methods [66, 79, 89, 106, 134, 135, 155, 177] may not significantly improve short-sprint performance, and in some cases, may impair performance. The primary methods training groups presented no significant change in short-sprint performance (i.e., 0–5 m SMD = − 0.13 [95% CI − 0.39, 0.13], 0–10 m SMD = − 0.04 [95% CI − 0.40, 0.33], 0–20 m SMD = 0.05 [95% CI − 0.08, 0.18]). The results of the primary training subgroup showed no significant improvement in short-sprint performance, contrasting with previous findings and the principle of specificity [43, 48]. This contradiction in findings with previous reviews [43, 48] suggests misclassification of the training methods used previously as these studies failed to include the additional training (e.g., resistance training) completed by the athletes, most probably as part of their usual training (e.g., [42, 105, 114–116, 176]). Therefore, previous review findings may support a combined approach of both specific and nonspecific training, not primary training alone [43, 48].

Maximal sprinting exposes the neuromuscular system to large forces (> 2 × bodyweight) produced during short ground contact periods (~ 0.080–0.200 s) performed at high movement velocities (7–10 m·s^−1^) resulting in both a coordinative overload and high neuromuscular stimulation [205, 207–209, 211, 232, 233]. The exposure to sprinting maximally, therefore, is expected to facilitate chronic physical and technical adaptations to improve short-sprint performance [204, 205, 207–209, 211, 232]. However, previous research in football code athletes reported that primary training methods showed no significant increase in stride length and a significant reduction in stride frequency despite a non-significant increase in sprint performance [155]. This is likely due to the included athlete population’s high chronic exposure to short-sprints with incomplete recovery as part of the demands of training and matches resulting in insufficient stimulus for neurological or morphological adaptations [222–225, 234]. Training guidelines for improving short-sprint performance recommend between 10–50 m sprints at > 98% maximal intensity with full recovery (1–2 min·s^−1^ of activity) between each sprint, to prevent a drop-off in performance [201]. However, most of the studies in this systematic review provide short incomplete rest periods between repetitions (e.g., 1–3 min between repetitions) for ~ 20–30 m sprints (3–4 to 4–5 s). Incomplete rest between sprints results in metabolic stress and exhaustion of energy substrate which may reduce the maximal sprint intensity by several mechanisms (e.g., weakened actin-myosin cross-bridges, reduction in the efficacy of signal transmission in the neuromuscular junction and alterations in neuromuscular activation [235–237]). Therefore, reducing the force and power load on the neuromuscular system when sprinting, reducing or preventing positive adaptations [106]. Longer training adaptation kinetics should also be considered, as the performance changes may have been affected by residual fatigue delaying the expression of an improved of performance output until after the testing incidence [201, 234, 238]. Therefore, primary training methods with short rests between sprints intervals is insufficient to improve short-sprint performance outcomes. Future studies should provide complete rest periods between maximal intensity sprints and test performance changes more frequently.

Technique drills are a component of the primary training subgroup. Technique drills simulate the sprinting action by isolating specific movements into more manageable components [201, 239]. For positive reinforcement of the technique to occur, the sprinting biomechanics must closely resemble the action and develop the athlete’s limiting factor [202, 240]. However, technique drills (e.g., A and B drills) are performed at much slower velocities than sprinting, and, therefore, they do not replicate sprinting from a kinematic standpoint [241]. Therefore, researchers have suggested that they have limited value, particularly when performed poorly, and they artificially constrain a sprinter’s technique [242, 243]. Although coaches [16] and training studies [104, 131] have included sprint technique drills in their training prescription, currently no studies in football code athletes have identified the training effects of inclusion and exclusion of running technique drills. When studies have included running drills, there is a limited explanation of the training prescription applied. Therefore, it may be more appropriate for technical speed training to address improvements in the magnitude and rate of force production on the ground and the mechanical efficiency (e.g., tertiary or secondary methods) [243]. Future studies should devote more attention to the effectiveness of sprint mechanics drills.

#### Secondary Methods

Secondary training modalities apply overload to the sprinting action by reducing (e.g., resisted sprinting) or increasing the movement speed allowing athletes to reach supramaximal velocities (e.g., assisted sprinting). Across the five studies, findings showed significant moderate improvements for all short-sprint performance outcomes (0–5 m SMD = 0.75 [95% CI 0.07, 1.43], 0–10 m SMD = 0.52 [95% CI 0.11, 0.93] and 0–20 m SMD = 0.64 [95% CI 0.05, 122]). Training adaptations are reported to be velocity change (%*V*_max_ increase vs reduction) specific [212, 244], with different significant distance specific improvements reported for secondary methods (i.e., assisted vs. resisted) [173]. The overload of the secondary training methods results in neurological or morphological adaptations allowing greater generation of ground reaction forces and improved mechanical efficiency to enhance short sprint performance [48, 52]. Resisted sprints have been shown to increase stride length and forward trunk lean (improved position to generate horizontal impulse) in track athletes [48, 52]. Whereas assisted methods have demonstrated increases in stride length and decreased stride frequency in track athletes [48, 52]. There are currently no studies measuring chronic kinematic changes in response to secondary training methods (no additional tertiary methods training) to support these in football code athletes. Of the two overload strategies, resisted sprint training [77, 79, 87, 89, 192] has received the greatest attention in the research in football code athletes despite significant improvements in both training methods (resisted [79, 89] and assisted [173]). Currently, no study has reported a statistically superior training effect between assisted and resisted training modes. Hence, it is unclear which training mode is the most effective for developing short-sprint performance. Therefore, secondary training methods are an effective method for coaches and athletes to improve short sprint performance outcomes. Further research is required to determine the optimal load and dose for performance enhancement.

#### Combined Specific Methods

Combined specific methods are a combination of both primary and secondary methods (e.g., sprinting and resisted sprinting). No previous review study has included combined specific training as a training method within their analysis, potentially due to the limited studies (*n* = 3) that have evaluated the effectiveness of such training methods [127, 128, 134]. Current findings showed significant moderate improvements in 0–5 m performance (SMD = 0.54 [95% CI 0.02, 1.07]) and large improvements in 0–10 m performance (SMD = 0.96 [95% CI 0.59, 1.33]) with small but no significant improvements at 0–20 m (SMD 0.32 [95% CI − 0.08, 0.72]). Improvements in short-sprint performance may occur from the enhancement of physical qualities (e.g., *F*_0_, *V*_0_, *P*_max_) [134] and mechanical improvements (increased stride length and frequency). For example, research has identified that ordering resisted sled pushing/pulling before unresisted sprint training can potentiate subsequent sprint performance [245, 246]. Therefore, increasing the neuromuscular stimulus, hence providing the necessary overload for this population to produce favourable neurological and or morphological adaptations. Such findings suggest that combined specific training methods may be an effective method for coaches and athletes to improve short-sprint performance outcomes; however, further research is warranted to support its effectiveness.

#### Tertiary Methods

Tertiary training methods are the most common within the research evidence base (*n* = 74) representing a wide range of training methods (e.g., strength, power, plyometrics [43, 247]). Although these training forms do not replicate the sprint running movement, they provide targeted stimuli to the underpinning mechanical components of the neuromuscular system that determine short-sprint performance (e.g., force–velocity–power and force–velocity profile) [26, 201, 212, 219].

The load velocity relationship enables practitioners to prescribe the appropriate resistance (bodyweight or external loads) of tertiary training methods to limit either or both the maximum velocity and force at which the maximum effort will occur [248]. This enables practitioners to target load specific adaptations using force–velocity–power orientated exercises in isolation or in combination (e.g., high-force/low-velocity vs low-force/ high-velocity vs peak power load) [26, 201, 212, 219].

Despite previous criticisms in the literature questioning the effectiveness for developing sprint performance (e.g., the duration available to apply force, no rotation-extension strategy, lack impact-limb deceleration mechanism [26]), significant moderate improvements were found for all short-sprint performance outcomes (0–5 m SMD = 0.64 [95% CI 0.42, 0.86], 0–10 m SMD = 0.56 [95% CI 0.42, 0.70], 0–20 m SMD = 0.40 [95% CI 0.30, 0.51]). Research comparing the kinetic factors underlying accelerative performance found that sprinters achieve higher maximum velocities compared to soccer athletes by attenuating the eccentric forces to a greater extent in the late braking phase and producing a higher antero‐posterior component of force across almost the entire propulsive phase [34]. Therefore, training methods that increase an athlete’s ability to produce sufficient vertical force, to withstand and reverse eccentric braking forces and to generate high antero‐posterior propulsive force are required, such as strength, power or plyometrics training [34, 201]. The improved physical qualities developed during tertiary training methods have been shown to manifest in significant improvements in short-sprint performance with associated reductions in stride frequency and increases in stride length [114, 116]. These findings present that high correspondence exists between the larger propulsive forces produced within the first few steps of short-sprints and the neural and morphological adaptations induced by these training methods [211].

Although performance increases are attributed to neurological or morphological adaptations from tertiary training methods, the morphological adaptations to these methods are often associated with increases in muscle cross-sectional area, such as non‐uniform regional hypertrophy of both the hip and thigh enabling greater force production capabilities advantageous for accelerating hip extension and producing greater propulsive forces [249–251]. Considerations should be made when training for increased mass development as an athlete gets heavier; they may not produce higher maximal force characteristics (*F*_0_) when normalised for body mass [203]. The force requirements to accelerate their body mass also increases, as does the aerodynamic drag resulting from a wider frontal surface area [203, 252]. Hence, chasing increases in body mass may be counterproductive for sprinting, at least when not moving an external mass [203]. Therefore, the results of the meta-analyses support Rumpf’s [43] previous findings, that tertiary training methods performed individually or in combination (e.g., strength power and plyometrics training) are an effective method for enhancing sprint performance. Rumpf’s lower sample of studies (*n* = 18–21 vs. *n* = 54–56) would have resulted in a greater sensitivity to larger negative training responses, therefore, explaining the lower reported training effects (effect size = 0.3–0.35 for 0–10 and 0–20 m respectively).

#### Combined Methods

Combined methods training includes both specific sprint training (primary and or secondary methods) and tertiary methods. Researchers, elite coaches, track and field sprinting coaches and team sports strength and conditioning coaches have suggested utilising an integrated approach utilising a combination of specific and non-specific methods is likely to be the most effective way to develop speed [16, 43, 204, 217, 253–255]. Elite coaches suggest a combined training approach using non-specific strength training or developing “gym strength” (e.g., heavy back squats) in conjunction with resisted sport-specific actions (e.g., resisted sprinting with sleds) would increase the chances of positive transfer [256]. This combination of both methods enables practitioners to provide stimuli to develop both the physical qualities of the lower limb and the mechanical efficiency concurrently [114, 116, 131, 182]. Previous studies, combining specific and tertiary training methods, demonstrated significant improvements in physical qualities [131], stride length and stride frequency [114, 116]. Current findings demonstrated that combined training methods (*n* = 49) were an effective method of developing short-sprint performance producing significant moderate-large SMD at 0–5 m (SMD 0.92 [95% CI 0.66, 1.18]), 0–10 m (SMD 0.60 [95% CI 0.44, 0.75]) and 0–20 m (SMD 0.56 [95% CI 0.39, 0.74]). Despite presenting the greatest training effects, each method presented different training methods (e.g., Electronic Supplementary Material Table S3). Therefore, combined specific methods are an effective training method for football code athletes and provide multiple options for practitioners and athletes to develop short-sprint performance. However, further research is required to identify the optimal combination of exercises and training loads to improve short-sprint performance.

### Moderator Variables

It is important to identify the moderator variables (i.e., football code, sex, age, playing standard, stage of the season) that may impact upon short-sprint training outcome [62]. Studies were not included in the analysis if the value used for subgroup analysis was not reported, failed to provide sufficient detail or did not match the appropriate moderator categories.

#### Sex

The meta-analysis of the intervention training groups found that both male and female football code athletes’ short-sprint performance can be improved. When comparing male and female athletes, there was no significant difference between the training effects. This should be taken within the context of the scarcity of the available information with female athletes training compared to males. No study was included that has compared the difference between training outcome by sex implementing matched training interventions in football code athletes. Therefore, despite the demonstrated differences between physical characteristics [4, 203] and endocrine response [257] to training between males and females, there is currently no sufficient evidence to suggest practitioners should approach developing short-sprint performance differently based on an athlete’s sex.

#### Playing Standard

Both elite and sub-elite cohort subgroups short-sprint performance was improved across all short-sprint outcomes. The between-group comparison identified the sub-elite populations had a significantly greater training effect than the elite populations at 0–5 m. There was no significant difference between the training effects for 0–10 m and 0–20 m. Previous research has identified that amateur players report larger benefits to specific training methods programs than elite athletes [258–260]. This is interesting considering research has demonstrated that lower performance standard populations, have large correlations between vertical and horizontal force–velocity–power variables [200]. Vertical profiles have been suggested to reflect the lower limbs neuromuscular maximal capabilities [200, 219]. This suggests that the ability to develop horizontal force during sprinting is associated with the ability of the lower limbs to develop force [200, 219]. Jiménez-Reyes [200] suggested that by training force and power production capabilities (e.g., strength and power training), it could effectively improve sprinting performance. However, in high playing standard populations (elite), horizontal force production during sprinting acceleration has a weaker association and is less determined by the maximal capabilities of the neuromuscular system to produce force. Therefore, further improvements may be represented by the ability to effectively apply horizontal force into the ground at progressively increasing velocities (mechanical effectiveness). Hence a greater focus on developing mechanical efficiency may be required (e.g., very heavy resisted sled sprints [134]). However, further research is required to discriminate between playing standards if there are mode-specific differences. Therefore, despite the demonstrated differences between physical characteristics between elite and sub-elite athletes [203] when considered independent of training status there is insufficient evidence to suggest practitioners should approach developing short sprint performance differently based on athlete’s playing standard within the football codes.

#### Age

Both senior and youth cohort subgroups short-sprint performance was enhanced following training interventions. However, between-group comparisons identified senior athletes enhanced short-sprint performance more than youth athletes at 0–10 m and 0–20 m. This finding is surprising with previous research showing that younger athletes typically have a greater training response compared to older counterparts [45, 157, 174]. Factors such as maturation may have moderated the training effects of the primary sprint training methods in male youth athletes with a greater training effect in pre- vs. mid-peak height velocity and early and late stage of maturation [45, 174]. This is supported by the finding that between age differences were found for sprint performance changes in younger athletes. These training effects suggest that within youth athlete cohorts, coaches should take into consideration, including chronological age, maturation, as well as the associated increase baseline performance levels and greater training experience [157]. However, further research is required to understand short-sprint performance outcomes by age, which could include maturity grouping.

#### Sport

Short-sprint performance was improved in soccer, futsal [113, 159, 171] and rugby union [103, 129, 175, 176] but not for rugby league [71, 72, 84, 93, 104] or rugby sevens [75, 182] in the 0–10 m distance outcome. Several football codes training subgroups had only 1–2 training groups due to limited representation in the literature for a given distance outcome (0–5 m Rugby league [*n* = 1] [84], 0–10 m and 0–20 m Australian rules football [*n* = 2] [162] and 0–20 m Futsal [*n* = 2] [171]). Therefore, these subgroup analyses were not considered. Despite the differences in physical characteristics [200, 203] and movement demands [222, 223] in the football codes, the only significant between-subgroup difference was in the 0–10 m performance outcome. The between-group comparison showed rugby sevens had a significantly lower change in performance than soccer, rugby union and futsal presenting a non-significant reduction in sprint performance. Although the data shows there are differences between football code subgroups, there is insufficient literature to demonstrate this consistently across all short sprint distance outcomes, and it is currently unclear whether these are specific to training methods or distance outcomes. No study was included that has compared the difference between training effects between football codes implementing matched training interventions in football code athletes on short-sprint performance. Therefore, despite the differences in physical characteristics presented between football codes, there is insufficient evidence to support coaches adapting short-sprint training methods based on football code.

#### Season

Both pre-season and in-season subgroups, short-sprint performance was improved, despite the typical reductions in the time available for practitioners to develop physical or movement qualities during the in-season period [47]. The off-season subgroup [64, 85, 136] presented no significant improvement. It is generally reported that fitness improvements are observed in the preseason, with a subsequent stabilisation of such fitness variables in-season [261]. Consequently, higher benefits are expected in trials performed during the preseason period compared with in-season [179, 259]. Therefore, if prescribed appropriately, there appears to be no significant difference of season between the training effects in football code athletes. The between-group comparison identified that both pre-season and in-season produced significantly larger improvements than off-season training in all performance outcomes. This has likely been skewed as a result of the large significant reduction in sprint performance observed in the study by Nakamura [136] due to the inclusion of only three training groups per outcome. No study was included that has compared the difference between training effects between the phase of the season implementing matched training interventions in football code athletes on short-sprint performance. Therefore, despite the differences in training demands between training phases, there is insufficient evidence to support coaches adapting short-sprint training methods based on the phase of the season.

### Limitations

This study represents the largest systematic review and meta-analysis of short-sprint performance development methods and the only to exclusively assess within and across football code athletes, but it is not without limitations. Firstly, this review classified training into training method groups (i.e., sport-only, primary, secondary, tertiary, combined and combined specific). While this improves on previous classifications [43, 44], it also fails to consider the complexity of short-sprint performance development considering training prescription, combined with the population and assessment methodologies. Therefore, the level of detail to fully understand short-sprint development is lacking, but this is difficult in the context of understanding sprint development and the multiple factors that interact. However, while we tried to consider numerous moderator variables (i.e., football code, sex, playing standard, age and phase of the season), this highlighted a further limitation of the existing research base in that research is mainly undertaken using parallel groups trials within male soccer athletes involving mainly tertiary training methods. Therefore, research including randomised control trials across the football codes, female cohorts, which utilise specific training methods are limited, which may impact the meta-analysis and moderator variable analysis and subsequent interpretation.

Sensitivity analysis of the data set revealed minor changes only, for the majority of performance variables, and these changes did not substantially impact the overall mean effect. However, the removal of the Mathisen and Danielsen [127] from the limited number of combined specific training subgroup moderated the statistical interpretation of the results. Although removing one or more studies independently did not affect the statistical significance of the tertiary methods the study by Asadi [74] should be scrutinised presenting an extreme performance improvement of 1.03 s (36.79%) in mean 20 m sprint performance. No other performance outcome improved by over 0.40 s (11.8%) [139]. While the limitations above exist, the information gathered from the current review with meta-analysis may support practitioners to use evidence-informed decisions when organising and evaluating training and generating future research.

### Future Research Directions

Future research investigating short-sprint performance development should be performed using high study quality designs (e.g., randomised control trials) examining the training effects in football codes outside of soccer (e.g., rugby codes, American, Australian rules, Gaelic football and futsal), world-class and successful elite athletes, trained populations with systematic training exposures and within female athlete cohorts. It should also be considered pairing subjects based on physical characteristics to establish a better understanding of whether training changes and adaptations are dependent upon resistance training experience and/or physical characteristics of the athlete. Future studies should consider modelling athlete’s velocity–time curve to reduce the limitations associated with pre and post sprint times or velocities [61]. This method would allow a better comparison between studies (improving future meta-analyses) while allowing practitioners to identify the mechanical profile of the athlete [262]. Combined modelling of velocity–time curves with the assessment of kinematic and kinetic changes performed at more frequent intervals would enable practitioners to isolate and confirm a time course of adaptations and the underlying causes to changes in performance [4, 200, 219]. A more comprehensive overview of developing football code athletes sprinting performance may be achieved by future research exploring the effectiveness of non-linear sprint interventions including change of directions variations (e.g., swerve running, 180° turns) as well as reviewing the development of RSA, non-linear and sprint outcomes > 20 m, due to their respective importance in the football codes.

Research has identified researchers, elite coaches, track and field sprinting coaches and team sport strength and conditioning coaches have suggested utilising an integrated approach utilising a combination of specific and nonspecific training performed individually in separate sessions or combinations (e.g., complex or contrast sets) to develop multiple physical qualities and skills simultaneously [16, 43, 204, 217, 253–255]. Therefore, further research would be better suited to manipulating the combinations, sequencing and loading parameters of combined specific and non-specific methods to enhance sprint performance longitudinally. This should be combined with methods of the profiling that allow optimisation and individualisation of training exposures [219, 247, 262–264]. This may reduce the variability in performance change [247]. While exercise specificity is certainly an important principle when developing a training program, it is only one of several principles that will influence the effectiveness of the program. Therefore, future research should continue to explore within and between-subgroups the effects of overload, variation, reversibility and the effect on sprint performance change [26]. Furthermore, this needs to be supported with determining the minimal and optimal training doses to retain and develop short-sprint performance in football code athletes. This will directly influence practitioner’s organisation of training and the prescribed loading variables.

## Conclusions

Short-sprint performance is an important attribute for football code athletes. Hence, establishing the most effective methods to improve performance is an important consideration for practitioners working across the football codes. This review provides the largest systematic review and meta-analysis comparing multiple training methods for developing short-sprint performance within football code athletes. An athlete's short-sprint performance is underpinned by their physical qualities (force–velocity–power) and mechanical effectiveness, thus providing practitioners with multiple methods of developing this quality. The included cohort of football code athletes (limited systematic exposure to the specific and nonspecific training stimulus) enhanced short-sprint performance (0–5, 0–10 m, 0–20 m) through secondary (i.e., resisted or assisted sprinting), tertiary (i.e., strength, power and plyometrics) and combined (i.e., primary or secondary and tertiary training methods) training modes. Combined specific training methods (i.e., primary and secondary methods) also improved short-sprint performance (0–5 and 0–10 m). However, based on this training mode alone, there is yet to be a presented most effective method. Both sport only training and primary training methods (i.e., sprinting, running drills) are insufficient to develop short-sprint performance. Moderator effects, although not mode-specific, suggest that there is not a consistent effect of age, sex, playing standard and phase of the season on short-sprint performance change. Regardless of the population characteristics, short-sprint performance can be enhanced by increasing either or both the magnitude and the orientation of force an athlete can generate and express in the sprinting action. These findings present practitioners with several options to suit their programme to enhance short-sprint performance, but future research needs to consider the interactions between training methodologies and applied practice to understand the impact of training on short-print performance fully. In conclusion, further high-quality research is warranted to confirm and possibly extend the results of this systematic meta-analytical review. Future studies should devote more attention to optimising and individualising training to maximise the training response; as well as the limited research into female cohorts, football code athletes outside of soccer, specific training methodologies.

## Electronic supplementary material

Below is the link to the electronic supplementary material.

Supplementary file1 (DOCX 96 kb)

Supplementary file2 (DOCX 72 kb)

Supplementary file3 (DOCX 28 kb)

## Declarations

### Availability of data and materials

The datasets generated during and/or analysed during the current study are available from the corresponding author on reasonable request.
