# Supplementary material for: The Training of Short Distance Sprint Performance in Football Code Athletes: A Systematic Review and Meta-Analysis
Source: Sports Med. 2020 Nov 27;51(6):1179–207. doi: 10.1007/s40279-020-01372-y (PMC8124057; doi:10.1007/s40279-020-01372-y)
Supplement: Supplementary file 1 — Supplementary file1 (DOCX 96 kb) [file 40279_2020_1372_MOESM1_ESM.docx]

**Electronic Supplementary Material Table S1**

Article title - The Training of Short Distance Sprint Performance in Football Code Athletes: A Systematic Review and Meta-Analysis

Journal name – Sports Medicine

Author names - Ben Nicholson, Alex Dinsdale, Ben Jones, and Kevin Till.

Affiliations - Leeds Beckett University, Carnegie Applied Rugby Research (CARR) centre, Carnegie School of Sport, Leeds, United Kingdom. Yorkshire Carnegie Rugby Union club, Leeds, United Kingdom. Leeds Rhinos Rugby League club, Leeds, United Kingdom. England Performance Unit, The Rugby Football League, Leeds, United Kingdom. School of Science and Technology, University of New England, Armidale, NSW, Australia. Division of Exercise Science and Sports Medicine, Department of Human Biology, Faculty of Health Sciences, the University of Cape Town and the Sports Science Institute of South Africa, Cape Town, South Africa.

corresponding author e-mail address – b.t.nicholson@leedsbeckett.ac.uk

**Table S1**

**Characteristics of the non-specific/tertiary sprint training groups included in the review**

| **Study (year)** | **Subjects** | **Training type and organisation** | **Training methods** | **Other training and testing equipment** | **Mean difference, 95% CI, percentage change, Std. Mean Difference IV, Random, 95% CI, weight, Qualitative inference** |
| --- | --- | --- | --- | --- | --- |
| Asadi et al. (2018) A (1) | M, n=10, Elite Soccer Players; Age 11.5±0.8 years | Plyometrics training 2d/wk, 6wks, 12 sessions, Pre-season | Moderate-high intensity plyometrics (2 sets of 10 foot contacts/set) of drop jumps from 20, 40, and 60 cm (60 foot contacts) on a grass surface | Soccer practice 3d/wk for 60–70 min (technical drills, tactical drills, simulated competitive games)  Timing gates (JBL Systems, Oslo, Norway). | 0-20m performance = MD (s): 0.18; 95% CI [-0.17, 0.53]; % Change 4.19%;  SMD: 0.22; 95% CI [-0.21, 0.66] Weight 1.06%; Inference - Small |
| Asadi et al. (2018) C (1) | M, n=10, Elite Soccer Players; Age 14±0.7 years | Plyometrics training 2d/wk, 6wks, 12 sessions, Pre-season | See Asadi et al. (2018) A | See Asadi et al. (2018) A | 0-20m performance ↑ MD (s): 0.29; 95% CI [0.09, 0.49]; % Change 8.22%;  SMD: 0.62; 95% CI [0.15, 1.09] Weight 1.03%; Inference - Moderate |
| Asadi et al. (2018) E (1) | M, n=10, Elite Soccer Players; Age 16.6±0.6 years | Plyometrics training 2d/wk, 6wks, 12 sessions, Pre-season | See Asadi et al. (2018) A | See Asadi et al. (2018) A | 0-20m performance ↑ MD (s): 1.03; 95% CI [0.82, 1.24]; % Change 36.79%;  SMD: 2.11; 95% CI [1.34, 2.88] Weight 0.78%; Inference - Large |
| Borges et al. (2016) B (2) | M, n=11, Elite Soccer Players; Age ± years | Plyometrics training 1-2d/wk, 7wks, 12 sessions, In-season | 2-7 sets of (10 foot contacts/set) high-intensity plyometrics (bi-lateral and unilateral box jumps and drop jumps) | 90-150 minutes soccer training session every morning, 5 times/wk, and one official match every Saturday  Photocell system | 0-5m performance ↓ MD (s): -0.05; 95% CI [-0.08, -0.02]; % Change -4.67%;  SMD: -0.76; 95% CI [-1.28, -0.23] Weight 2.51%; Inference - Moderate |
| Bouguezzi et al. (2018) A (3) | M, n=15, Elite Soccer Players; Age 11.32±0.27 years | Plyometrics training 1d/wk, 8wks, 16 sessions, In-season | 3-6 sets of 8-14 reps/set of moderate-intensity plyometric training of unilateral and bilateral acyclical exercises (CMJ's, ankle Hops, zig-zags) | 3-4 soccer training sessions/wk  Electronic timing system (Microgate SARL, Bolzano, Italy) | 0-5m performance = MD (s): 0.03; 95% CI [-0.01, 0.07]; % Change 2.52%;  SMD: 0.31; 95% CI [-0.09, 0.72] Weight 2.66%; Inference - Small  0-10m performance = MD (s): 0.03; 95% CI [-0.03, 0.09]; % Change 1.44%;  SMD: 0.19; 95% CI [-0.19, 0.58] Weight 1.24%; Inference - Trivial  0-20m performance = MD (s): 0.06; 95% CI [-0.05, 0.17]; % Change 1.61%;  SMD: 0.2; 95% CI [-0.15, 0.55] Weight 1.12%; Inference - Small |
| Bouguezzi et al. (2018) B (3) | M, n=15, Elite Soccer Players; Age 12.27±0.33 years | Plyometrics training 2d/wk, 8wks, 16 sessions, In-season | 6-12 sets of 8-12 reps/set of moderate intensity plyometric training of unilateral and bilateral acyclical exercises (CMJ's, ankle Hops, zig-zags) | See Bouguezzi et al. (2018) A | 0-5m performance ↑ MD (s): 0.06; 95% CI [0.03, 0.09]; % Change 5.17%;  SMD: 0.86; 95% CI [0.39, 1.32] Weight 2.59%; Inference - Large  0-10m performance ↑ MD (s): 0.05; 95% CI [0.02, 0.08]; % Change 2.43%;  SMD: 0.58; 95% CI [0.17, 0.99] Weight 1.22%; Inference - Moderate  0-20m performance = MD (s): -0.01; 95% CI [-0.06, 0.04]; % Change -0.27%;  SMD: -0.07; 95% CI [-0.42, 0.28] Weight 1.12%; Inference - Trivial |
| Brito et al. (2014) A (4) | M, n=12, Sub-elite Soccer Players; Age 20.3±0.9 years | Strength training 2d/wk, 9wks, 18 sessions, In-season | High intensity (80-90%) LB strength training low volume (1 set of 6 reps/set) squat at 90°, calf extensions and leg extensions. Strength exercises increased by 5% from 1-RM every 3 wks. | Routine soccer training, based on technical and tactical drills, and small-sided games.  Photoelectric cells (Speed Trap II, Brower Timing Systems, USA) | 0-5m performance ↑ MD (s): 0.05; 95% CI [0.04, 0.06]; % Change 3.99%;  SMD: 1.67; 95% CI [0.98, 2.36] Weight 2.28%; Inference - Large  0-20m performance ↑ MD (s): 0.16; 95% CI [0.14, 0.18]; % Change 5.25%;  SMD: 3.4; 95% CI [2.38, 4.43] Weight 0.6%; Inference - Large |
| Chelly et al. (2010) A (5) | M, n=12, Elite Soccer Players; Age 19.1±0.7 years | Strength and plyometrics training 2d/wk, 8wks, 16 sessions, In-season | Moderate-high volume (10 sets of 4-10 reps/set) high-intensity plyometric training (wk1-4 40-60cm hurdle jumps and wk 5-8 40cm bilateral drop jumps) on a grass track and a light UB and LB resistance training programme (not identified) | Weekly school physical education sessions; these lasted for 40 minutes and consisted mainly of ball games.  Video cameras (Sony Handycam, DCRPC105E, 2003 Sony corporation, Tokyo, Japan) | 0-5m performance ↑ MD (m·s^-1^): 0.4; 95% CI [0.19, 0.61]; % Change 10%;  SMD: 0.86; 95% CI [0.34, 1.38] Weight 2.52%; Inference - Large |
| Christou et al. (2006) A (6) | M, n=9, Elite Soccer Players; Age 13.8±0.4 years | Strength training 2d/wk, 16wks, 32 sessions, In-season | Moderate-high load (55-80% 1RM) FB strength training 2-3 sets of 8-15 reps/set of leg press, bench press, leg extension, | Strength training 2d/wk, 16wks, 32 sessions, In-season | 0-10m performance ↑ MD (s): 0.07; 95% CI [0.04, 0.1]; % Change 3.35%;  SMD: 1.25; 95% CI [0.6, 1.91] Weight 1.04%; Inference - Large |
| Comfort et al. (2012) A (7) | M, n=19, Elite Rugby league Players; Age ± years | Strength, power and plyometrics training 2d/wk, 8wks, 16 sessions, Pre-season | 4wk strength mesocycle and 4wk power mesocycle along with 2 additional plyometric and agility sessions/wk (not specified). Strength microcycle: high load (85-90%1RM) LB strength exercises. These are separated into a heavy day (4 sets of 4-6 reps/set) and light a day (2 sets of 2-4 reps/set) back squat, midthigh clean pull, RDL and nordic curls. Power microcycle: high load 85% 1RM LB strength/power exercises. These are separated into a heavy day (2-4 sets of 3-6 reps/set) and a light day (2-4 sets of 2-3 reps/set) hang power clean, squat jumps, back squat and nordic curls. | NA.  Infrared timing gates (Brower, Speed Trap 2, Wireless Timing System, UT, USA) | 0-5m performance ↑ MD (s): 0.09; 95% CI [0.06, 0.12]; % Change 9.41%;  SMD: 1.19; 95% CI [0.72, 1.65] Weight 2.59%; Inference - Large  0-10m performance ↑ MD (s): 0.13; 95% CI [0.1, 0.16]; % Change 8.09%;  SMD: 1.39; 95% CI [0.92, 1.86] Weight 1.18%; Inference - Large  0-20m performance ↑ MD (s): 0.2; 95% CI [0.16, 0.24]; % Change 6.97%;  SMD: 1.78; 95% CI [1.28, 2.28] Weight 1%; Inference - Large |
| Coratella et al. (2019) A (8) | M, n=16, Sub-elite Soccer Players; Age 21±3 years | Power training 2d/wk, 8wks, 16 sessions, Off-season | 2-5 sets of 10 reps/set of BW jump squat power training | NA  Infrared device (Polifemo, Microgate, Bolzano, Italy) | 0-10m performance = MD (s): 0; 95% CI [-0.04, 0.04]; % Change 0%;  SMD: 0; 95% CI [-0.37, 0.37] Weight 1.25%; Inference - Trivial |
| Coratella et al. (2019) B (8) | M, n=16, Sub-elite Soccer Players; Age 21±3 years | Power training 2d/wk, 8wks, 16 sessions, Off-season | 2-4 sets of 10-11 reps/set of +20-25%BW jump squat power training | See Coratella et al. (2019) A | 0-10m performance ↑ MD (s): 0.2; 95% CI [0.13, 0.27]; % Change 11.11%;  SMD: 1; 95% CI [0.55, 1.45] Weight 1.19%; Inference - Large |
| Coutts et al. (2004) A (9) | M, n=21, Elite Rugby league Players; Age 16.8±1 years | Strength training 3d/wk, 6wks, 36 sessions, Phase not reported | Supervised FB strength training moderate-high load (55-73.5 %1RM) high-low volume (10-16 reps/set) back squat, wide grip pulldown behind, bench press, front military press, shoulder dips (weighted), abdominal crunches, back extensions. | NA  Electronic timing gates (Swift, Goonellabah, Australia). | 0-10m performance = MD (s): 0.02; 95% CI [-0.01, 0.05]; % Change 0.93%;  SMD: 0.25; 95% CI [-0.08, 0.58] Weight 1.27%; Inference - Small  0-20m performance = MD (s): 0.01; 95% CI [-0.01, 0.03]; % Change 0.29%;  SMD: 0.12; 95% CI [-0.17, 0.42] Weight 1.16%; Inference - Trivial |
| Coutts et al. (2004) B (9) | M, n=21, Elite Rugby league Players; Age 16.8±1 years | Strength and power training 3d/wk, 12wks, 36 sessions, Phase not reported | Supervised FB resistance training. Phase 1 6wks: moderate-high load (55-73.5%1RM) high volume (10-16 reps/set) back squat, wide grip pulldown behind, bench press, front military press, shoulder dips (weighted), abdominal crunches, back extensions. Phase 2 6wks: moderate-high load (73.5%- 88.5%1RM) moderate-high volume (4-9 reps/set) back squat, box jumps, clean pull, bench press, push press, chin-ups (weighted), abdominal crunches, hamiglute raise. | See Coutts et al. (2004) A | 0-10m performance = MD (s): 0.02; 95% CI [-0.01, 0.05]; % Change 0.93%;  SMD: 0.25; 95% CI [-0.08, 0.58] Weight 1.27%; Inference - Small  0-20m performance ↑ MD (s): 0.03; 95% CI [0.01, 0.05]; % Change 0.87%;  SMD: 0.37; 95% CI [0.07, 0.68] Weight 1.16%; Inference - Small |
| Coutts et al. (2004) C (9) | M, n=21, Elite Rugby league Players; Age 16.6±1.2 years | Strength training 3d/wk, 6wks, 36 sessions, Phase not reported | Unsupervised FB resistance training moderate-high load (55-73.5 %1RM) high-low volume (10-16 reps/set) back squat, wide grip pulldown behind, bench press, front military press, shoulder dips (weighted), abdominal crunches, back extensions. | See Coutts et al. (2004) A | 0-10m performance = MD (s): 0.03; 95% CI [0, 0.06]; % Change 0.87%;  SMD: 0.28; 95% CI [-0.04, 0.61] Weight 1.27%; Inference - Small  0-20m performance = MD (s): 0.03; 95% CI [0, 0.06]; % Change 0.87%;  SMD: 0.28; 95% CI [-0.02, 0.59] Weight 1.16%; Inference - Small |
| Coutts et al. (2004) D (9) | M, n=21, Elite Rugby league Players; Age 16.6±1.2 years | Strength and power training 3d/wk, 12wks, 36 sessions, Phase not reported | Unsupervised FB resistance training. Phase 1 6wks: moderate-high load (55-73.5%1RM) high volume (10-16 reps/set) Back squat, wide grip pulldown behind, bench press, front military press, shoulder dips (weighted), abdominal crunches, back extensions. Phase 2 6wks: moderate-high load (73.5%- 88.5%1RM) moderate-high volume (4-9 reps/set) back squat, box jumps, clean pull, bench press, push press, chin-ups (weighted), abdominal crunches, hamiglute raise. | See Coutts et al. (2004) A | 0-10m performance ↑ MD (s): 0.04; 95% CI [0.01, 0.07]; % Change 1.17%;  SMD: 0.39; 95% CI [0.06, 0.72] Weight 1.27%; Inference - Small  0-20m performance ↑ MD (s): 0.04; 95% CI [0.01, 0.07]; % Change 1.17%;  SMD: 0.39; 95% CI [0.08, 0.69] Weight 1.16%; Inference - Small |
| De Hoyo et al. (2015) A (10) | M, n=18, Elite Soccer Players; Age 18±1 years | Strength training 1-2d/wk, 10wks, 17 sessions, In-season | Eccentric-overload training program 3–6 sets of 6 reps/set of half squat and prone leg-curl exercises using a Yo-Yo isoinertial flywheel device | Soccer training with a similar weekly training volume and methodology (4 or 5 sessions/wk of 60–90 min and 1 match/ wk)  Electronic timing gates (Race Time 2 Light Radio System; Microgate, Bolzano, Italy). | 0-10m performance = MD (s): 0.02; 95% CI [-0.02, 0.06]; % Change 1.17%;  SMD: 0.18; 95% CI [-0.17, 0.53] Weight 1.26%; Inference - Trivial  0-20m performance = MD (s): 0.04; 95% CI [0, 0.08]; % Change 1.34%;  SMD: 0.3; 95% CI [-0.03, 0.63] Weight 1.14%; Inference - Small |
| De Hoyo et al. (2016) A (11) | M, n=11, Elite Soccer Players; Age 18±1 years | Power training 2d/wk, 8wks, 16 sessions, In-season | Light-moderate loads squat (40-60%1RM) moderate volume (2-3 sets of 4-8 reps/set) using a full range back squat | All players participated on an average of; 10 hrs of combined soccer (4– 5 sessions) and conditioning (1 session) training, and 1 competitive match per week.  Dual-beam electronic timing gate OptoJump System (Polifemo Radio Light, Microgate, Bolzano, Italy) | 0-10m performance = MD (s): -0.01; 95% CI [-0.04, 0.02]; % Change -0.6%;  SMD: -0.13; 95% CI [-0.58, 0.31] Weight 1.2%; Inference - Trivial  0-20m performance = MD (s): 0.01; 95% CI [-0.03, 0.05]; % Change 0.34%;  SMD: 0.1; 95% CI [-0.31, 0.51] Weight 1.08%; Inference - Trivial |
| Douglas et al. (2018) A (12) | M, n=7, Elite Rugby Players; Age 19.4±0.8 years | Strength and power training 2d/wk, 4wks, 8 sessions, In-season | 2 strength sessions/wk + 1 power session. Strength sessions: AEL smith machine back squat, assistance LB and UB assistance exercise for 3–4 sets of 6–8 reps/set. Power training session: AEL broad jump (30% BM), partner AEL KB swing and an assistance FB power for 3–4 sets of 4–6 reps/set. | A combination of conditioning- and skill-based field sessions, and a club rugby game each week  Radar gun device (Stalker ATS II; Applied Concepts, Dallas, TX, USA) | 0-10m performance = MD (s): 0.01; 95% CI [-0.02, 0.04]; % Change 0.56%;  SMD: 0.18; 95% CI [-0.38, 0.74] Weight 1.11%; Inference - Trivial  0-20m performance = MD (s): 0.03; 95% CI [-0.01, 0.07]; % Change 0.98%;  SMD: 0.36; 95% CI [-0.17, 0.88] Weight 0.98%; Inference - Small |
| Douglas et al. (2018) B (12) | M, n=7, Elite Rugby Players; Age 19.4±0.8 years | Strength and power training 2d/wk, 10wks, 18 sessions, In-season | 4 wks slow + 4 weeks fast accentuated eccentric training consisting of 2 strength sessions/wk + 1 power session. Slow Strength sessions: AEL smith machine back squat, assistance LB and UB assistance exercise for 2–4 sets of 6–8 reps/set. Fast strength sessions: AEL smith machine back squat, assistance LB and UB assistance exercise for 2–4 sets of 4–5 reps/set. Slow Power training session: AEL broad jump (30% BM), partner AEL KB swing and an assistance FB power for 3–4 sets of 4–6 reps/set. Fast power training session: 50cm AEL drop Jump (20% BM), partner AEL banded KB Swing, assistance FB power exercise. | See Douglas et al. (2018) A | 0-10m performance ↓ MD (s): -0.03; 95% CI [-0.06, 0]; % Change -1.64%;  SMD: -0.64; 95% CI [-1.25, -0.03] Weight 1.08%; Inference - Moderate  0-20m performance = MD (s): -0.03; 95% CI [-0.07, 0.01]; % Change -0.96%;  SMD: -0.43; 95% CI [-0.96, 0.11] Weight 0.97%; Inference - Moderate |
| Douglas et al. (2018) C (12) | M, n=7, Elite Rugby Players; Age 19.4±0.8 years | Strength and power training 2d/wk, 4wks, 8 sessions, In-season | Slow strength training consisting of 2 strength sessions/wk + 1 power session. Strength sessions: back squat, assistance LB and UB assistance exercise for 3–4 sets of 6–8 reps/set. Power training session: broad jump (30% BM), KB swing and an assistance FB power exercise for 3–4 sets of 4–6 reps/set. | See Douglas et al. (2018) A | 0-10m performance = MD (s): 0.02; 95% CI [-0.04, 0.08]; % Change 1.06%;  SMD: 0.17; 95% CI [-0.39, 0.73] Weight 1.11%; Inference - Trivial  0-20m performance = MD (s): 0.02; 95% CI [-0.07, 0.11]; % Change 0.63%;  SMD: 0.12; 95% CI [-0.4, 0.63] Weight 0.99%; Inference - Trivial |
| Douglas et al. (2018) D (12) | M, n=7, Elite Rugby Players; Age 19.4±0.8 years | Strength and power training 2d/wk, 10wks, 18 sessions, In-season | 4 wks slow + 4 weeks fast strength training consisting of 2 strength/wk + 1 power session. Slow strength sessions: back squat, assistance LB and UB assistance exercise for 2–4 sets of 6–8 reps/set. Fast strength sessions: back squat, assistance LB and UB assistance exercise for 2–4 sets of 4–5 reps/set. Slow power training session: broad jump, KB swing and an assistance FB power exercise for 3–4 sets of 4–6 reps/set. Fast power training session: 50cm drop Jump, banded KB, assistance FB power exercise. | See Douglas et al. (2018) A | 0-10m performance = MD (s): 0.01; 95% CI [-0.05, 0.07]; % Change 0.53%;  SMD: 0.09; 95% CI [-0.46, 0.65] Weight 1.12%; Inference - Trivial  0-20m performance = MD (s): 0; 95% CI [-0.08, 0.08]; % Change 0%;  SMD: 0; 95% CI [-0.51, 0.51] Weight 0.99%; Inference - Trivial |
| Gabbett et al. (2008) A (13) | M, n=14, Elite Rugby league Players; Age 14.1±0.2 years | Strength training 3d/wk, 10wks, 30 sessions, Pre-season | Moderate load strength training (2-3 sets of 12-15 reps/set) for 6wks followed by (3-4 sets of 8 to 12 reps/set) for 4wks of LB (leg press) and UB (bench press, shoulder press, wide grip pull-down, and chin-ups) using machine weights | Repeated sprint ability training as well as pre-season technical, tactical training  Dual-beam electronic timing gates (Swift Performance Equipment). | 0-10m performance ↑ MD (s): 0.01; 95% CI [0, 0.02]; % Change 0.54%;  SMD: 0.5; 95% CI [0.08, 0.92] Weight 1.22%; Inference - Moderate  0-20m performance ↑ MD (s): 0.03; 95% CI [0.02, 0.04]; % Change 0.95%;  SMD: 0.8; 95% CI [0.38, 1.22] Weight 1.07%; Inference - Large |
| Gabbett et al. (2008) B (13) | M, n=21, Elite Rugby league Players; Age 16.9±0.3 years | Strength training 3d/wk, 10wks, 30 sessions, Pre-season | See Gabbett et al. (2008) A | See Gabbett et al. (2008) A | 0-10m performance ↓ MD (s): -0.04; 95% CI [-0.05, -0.03]; % Change -2.14%;  SMD: -2; 95% CI [-2.55, -1.45] Weight 1.12%; Inference - Large  0-20m performance ↓ MD (s): -0.1; 95% CI [-0.11, -0.09]; % Change -3.09%;  SMD: -2.66; 95% CI [-3.3, -2.03] Weight 0.89%; Inference - Large |
| Garci´a-Pinillos et al. (2014) A (14) | M, n=17, Elite Soccer Players; Age 15.47±1.28 years | Strength and plyometrics training 2d/wk, 12wks, 24 sessions, In-season | Moderate volume 4-6 sets of isometric wall squat (half squat 40–80 seconds) and low-moderate intensity plyometric training using jumping from a seated position and single-leg jumping using arm swing (6 reps/set). | Soccer training 3 times/wk and played competitive matches at least once/wk  Casio Exilim EXZR-10 high speed camera (Dover, NJ, USA) | 0-5m performance ↑ MD (s): 0.25; 95% CI [0.17, 0.33]; % Change 17.61%;  SMD: 1.17; 95% CI [0.69, 1.66] Weight 2.56%; Inference – Large  0-10m performance ↑ MD (s): 0.33; 95% CI [0.23, 0.43]; % Change 15.42%;  SMD: 1.17; 95% CI [0.71, 1.63] Weight 1.18%; Inference - Large  0-20m performance ↑ MD (s): 0.32; 95% CI [0.23, 0.41]; % Change 9.12%;  SMD: 1.17; 95% CI [0.74, 1.6] Weight 1.06%; Inference - Large |
| González-García et al. (2019) A (15) | F, n=8 Sub-elite Soccer Players; Age 16.8±1.6 years | Strength training 2d/wk, 7wks, 14 sessions, In-season | Moderate-high load (60-90%1RM) LB strength training 4 sets of 4-12 reps/set of back squat. | Soccer training 3 times/wk  Single-beam timing lights (DSD Sport system, León, Spain) | 0-10m performance = MD (s): -0.02; 95% CI [-0.05, 0.01]; % Change -1.51%;  SMD: -0.3; 95% CI [-0.83, 0.23] Weight 1.14%; Inference - Small  0-20m performance = MD (s): -0.01; 95% CI [-0.08, 0.06]; % Change -0.21%;  SMD: -0.07; 95% CI [-0.55, 0.41] Weight 1.02%; Inference - Trivial |
| González-García et al. (2019) B (15) | F, n=6 Sub-elite Soccer Players; Age 16.8±1.6 years | Strength training 2d/wk, 7wks, 14 sessions, In-season | Moderate-high load (60-90%1RM) LB strength training 4 sets of 4-12 reps/set of hip thrust. | See González-García et al. (2019) A | 0-10m performance = MD (s): 0.07; 95% CI [-0.02, 0.16]; % Change 3.74%;  SMD: 0.46; 95% CI [-0.17, 1.09] Weight 1.06%; Inference - Moderate  0-20m performance = MD (s): 0.09; 95% CI [-0.03, 0.21]; % Change 2.67%;  SMD: 0.41; 95% CI [-0.17, 0.98] Weight 0.94%; Inference - Moderate |
| Griffiths et al. (2019) A (16) | M, n=15, Sub-elite Soccer Players; Age 21±1 years | Strength training 2d/wk, 6wks, 12 sessions, In-season | High load (80%1RM) FB strength training for 3 sets of reps to muscular failure (Smith machine squats, knee extension, knee flexion, hip extension, hip flexion, heel raises, chest press, and pull-down) using a fixed 2-second eccentric and 2-second concentric (2:2) rep duration. | Soccer-specific training  Brower TC Timing System (Utah, USA) | 0-10m performance ↑ MD (s): 0.05; 95% CI [0.02, 0.08]; % Change 2.24%;  SMD: 0.58; 95% CI [0.17, 0.99] Weight 1.22%; Inference - Moderate |
| Griffiths et al. (2019) B | M, n=15, Sub-elite Soccer Players; Age 23±1 years | Strength training 2d/wk, 6wks, 12 sessions, In-season | High load (80%1RM) FB strength training for 3 sets of reps to muscular failure (Smith machine squats, knee extension, knee flexion, hip extension, hip flexion, heel raises, chest press, and pull-down) using a maximal intended velocity for both concentric and eccentric phases. | See Griffiths et al. (2019) A | 0-10m performance = MD (s): 0.05; 95% CI [0, 0.1]; % Change 2.2%;  SMD: 0.38; 95% CI [-0.01, 0.77] Weight 1.23%; Inference - Small |
| Hammami et al. (2016) 1A (17) | M, n=15, Soccer Players; Age 15.7±0.2 years | Plyometrics training 2d/wk, 8wks, 16 sessions, In-season | High-intensity moderate volume (4-10 sets of 7-10 foot contacts/set) plyometric training (hurdle jumps 50-60cm and drop jumps 60-70cm) | Soccer training 4-5 times/wk, and one official soccer match every Sunday  Photocells system (Microgate, Bolzano, Italy) | 0-5m performance ↑ MD (s): 0.08; 95% CI [0.05, 0.11]; % Change 7.92%;  SMD: 0.97; 95% CI [0.48, 1.45] Weight 2.57%; Inference - Large  0-10m performance ↑ MD (s): 0.11; 95% CI [0.07, 0.15]; % Change 6.29%;  SMD: 1.07; 95% CI [0.6, 1.55] Weight 1.18%; Inference - Large  0-20m performance ↑ MD (s): 0.15; 95% CI [0.1, 0.2]; % Change 4.87%;  SMD: 1; 95% CI [0.57, 1.43] Weight 1.06%; Inference - Large |
| Hammami et al. (2018) B (18) | M, n=14, Elite Soccer Players; Age 15.7±0.2 years | Plyometrics training 2d/wk, 8wks, 16 sessions, In-season | High-intensity moderate volume (4-10 sets of 7-10 foot contacts/set) plyometric training (hurdle jumps 50-60cm and drop jumps 60-70cm) on a tartan track | Soccer training 4–5 times/wk and 1 official game/wk  Paired photocell timers (Microgate, Bolzano, Italy) | 0-5m performance ↑ MD (s): 0.08; 95% CI [0.05, 0.11]; % Change 8%;  SMD: 0.95; 95% CI [0.45, 1.45] Weight 2.55%; Inference - Large |
| Harries et al. (2017) A (19) | M, n=8, Elite Rugby union Players; Age 16.8±1 years | Strength and plyometrics training 2d/wk, 12wks, 24 sessions, Phase not reported | Moderate-high load (60-90% 1RM) moderate volume (4-6 sets of 3-10 reps/set) linear perioidised FB strength training programme (back squat, UB pull, abdominal exercises, bench press, hip dominant exercise, loaded carries (20-40m) gluteham raises + 2-5 sets of 2-4 reps of jumps (jumps not specified) | 2 sessions/wk rugby team practice  Electronic timing system (Fitness Technology, Skye, SA, Australia) | 0-10m performance ↑ MD (s): 0.03; 95% CI [0.01, 0.05]; % Change 1.6%;  SMD: 0.81; 95% CI [0.21, 1.41] Weight 1.09%; Inference - Large  0-20m performance = MD (s): 0.02; 95% CI [-0.02, 0.05]; % Change 0.5%;  SMD: 0.2; 95% CI [-0.29, 0.68] Weight 1.02%; Inference - Small |
| Harries et al. (2017) B (19) | M, n=8, Elite Rugby union Players; Age 17±1.1 years | Strength and plyometrics training 2d/wk, 12wks, 24 sessions, Phase not reported | Moderate-high load (60-90% 1RM) moderate volume (4-6 sets of 3-10 reps/set) daily undulating perioidised FB strength training programme (back squat, UB pull, abdominal exercises, bench press, hip domiant exercise, loaded carries (20-40m) gluteham raises + 2-5 sets of 2-4 reps of jumps (jumps not specified) | See Harries et al. (2017) A | 0-10m performance = MD (s): 0.04; 95% CI [-0.02, 0.1]; % Change 2.6%;  SMD: 0.33; 95% CI [-0.2, 0.86] Weight 1.13%; Inference - Small  0-20m performance = MD (s): 0.05; 95% CI [-0.03, 0.14]; % Change 1.69%;  SMD: 0.29; 95% CI [-0.2, 0.78] Weight 1.01%; Inference - Small |
| Helgerud et al. (2011) A (20) | M, n=21, Elite Soccer Players; Age 25.25±3.2 years | Strength training 2d/wk, 8wks, 16 sessions, Pre-season | High load, low volume LB strength training (half squats) 4 sets of 4 reps/set | Soccer training 6 sessions/wk 1.5hr practice sessions plus a match  Photocells (Brower Timing Systems, Draper, UT, USA) | 0-10m performance ↑ MD (s): 0.06; 95% CI [0.04, 0.08]; % Change 3.61%;  SMD: 0.81; 95% CI [0.44, 1.18] Weight 1.25%; Inference - Large  0-20m performance ↑ MD (s): 0.05; 95% CI [0.02, 0.08]; % Change 1.9%;  SMD: 0.47; 95% CI [0.16, 0.78] Weight 1.15%; Inference - Moderate |
| Impellizzeri et al. (2007) A (21) | M, n=18, Sub-elite Soccer Players; Age 25±4 years | Plyometrics training 3d/wk, 4wks, 12 sessions, Pre-season | Low-high intensity high volume (3-25 sets of 5-15 foot contacts/set) plyometrics training (vertical jumps, bounding, broad jumps, drop jumps) on a grass pitch surface | Players trained 3 sessions/wk and 1 official match/wk, no plyometric exercises or sprint training were completed. Players also completed their usual training consisting of generic and specific aerobic interval training and technical-tactical training.  Two telemetric photocells (Microgate, Bolzano, Italy) | 0-10m performance ↑ MD (s): 0.07; 95% CI [0.04, 0.1]; % Change 3.85%;  SMD: 0.92; 95% CI [0.51, 1.33] Weight 1.22%; Inference - Large  0-20m performance ↑ MD (s): 0.09; 95% CI [0.06, 0.12]; % Change 2.87%;  SMD: 1.05; 95% CI [0.65, 1.44] Weight 1.09%; Inference - Large |
| Impellizzeri et al. (2007) B (21) | M, n=19, Sub-elite Soccer Players; Age 25±4 years | Plyometrics training 3d/wk, 4wks, 12 sessions, Pre-season | See Impellizzeri et al. (2007) A on a 0.2 m deep dry sand surface | See Impellizzeri et al. (2007) A | 0-10m performance ↑ MD (s): 0.08; 95% CI [0.05, 0.11]; % Change 4.44%;  SMD: 0.78; 95% CI [0.39, 1.16] Weight 1.24%; Inference - Moderate  0-20m performance ↑ MD (s): 0.08; 95% CI [0.04, 0.12]; % Change 2.57%;  SMD: 0.57; 95% CI [0.23, 0.9] Weight 1.14%; Inference - Moderate |
| Ishøi et al. (2017) A (22) | M, n=10, Sub-elite Soccer Players; Age 19.1±1.8 years | Strength training 1-3d/wk, 10wks, 27 sessions, In-season | 2-3 sets of 5-12 reps of nordic curl strength training | 3 soccer training sessions/wk (approx. 5–7 hrs of training) and one match/wk.  Dual-beam photoelectric cells (Witty Wireless Training Timer, Microgate, Bolzano, Italy) | 0-10m performance = MD (s): 0.04; 95% CI [0, 0.09]; % Change 2.65%;  SMD: 0.47; 95% CI [-0.02, 0.96] Weight 1.17%; Inference - Moderate |
| Kobal et al. (2017) 1A (23) | M, n=9, Elite Soccer Players; Age 18.9±0.6 years | Strength and plyometrics training 2d/wk, 8wks, 16 sessions, In-season | Complex training (all sets of strength training followed by all sets of plyometric Training). Moderate-high load back half squat 6-10 reps/set and bilateral drop jumps (30-45cm) 10-12 foot contacts/per set | Soccer training 5 sessions/wk, and one official soccer match every Saturday  Photocells (Smartspeed System; Fusion Sport) | 0-10m performance ↑ MD (s): 0.17; 95% CI [0.12, 0.22]; % Change 3.12%;  SMD: 1.83; 95% CI [1.03, 2.63] Weight 0.93%; Inference - Large  0-20m performance ↑ MD (s): 0.21; 95% CI [0.17, 0.25]; % Change 3.18%;  SMD: 2.33; 95% CI [1.46, 3.21] Weight 0.7%; Inference - Large |
| Kobal et al. (2017) 1B (23) | M, n=9, Elite Soccer Players; Age 18.9±0.6 years | Strength and plyometrics training 2d/wk, 8wks, 16 sessions, In-season | Traditional (all sets of plyometric training performed before all sets of strength training). Moderate-high load back half squat 6-10 reps/set and drop jumps (30-45cm) 10-12 foot contacts/per set | See Kobal et al. (2017) A | 0-10m performance ↑ MD (s): 0.37; 95% CI [0.26, 0.48]; % Change 7.04%;  SMD: 1.61; 95% CI [0.87, 2.35] Weight 0.98%; Inference - Large  0-20m performance ↑ MD (s): 0.38; 95% CI [0.29, 0.47]; % Change 6.03%;  SMD: 1.83; 95% CI [1.09, 2.57] Weight 0.8%; Inference - Large |
| Kobal et al. (2017) 1C (23) | M, n=9, Elite Soccer Players; Age 18.9±0.6 years | Strength and plyometrics training 2d/wk, 8wks, 16 sessions, In-season | Contrast (sets of strength training alternated with sets of plyometric training in a set-by-set fashion). Moderate-high load back half squat 6-10 reps/set and drop jumps (30-45cm) 10-12 foot contacts/per set | See Kobal et al. (2017) A | 0-10m performance = MD (s): 0; 95% CI [-0.09, 0.09]; % Change -0.03%;  SMD: 0; 95% CI [-0.49, 0.49] Weight 1.17%; Inference - Trivial  0-20m performance ↑ MD (s): 0.1; 95% CI [0.01, 0.19]; % Change 1.54%;  SMD: 0.51; 95% CI [0.03, 0.99] Weight 1.02%; Inference - Moderate |
| Kobal et al. (2017) 2A (24) | M, n=9, Elite Soccer Players; Age 15.9±1.2 years | Plyometrics training 2d/wk, 6wks, 12 sessions, Pre-season | 2-6 sets of 6 reps/set of vertical and horizontal loaded jumps with an additional 8% BW load | Soccer training 5 times /wk  Wireless single-beam light gates (Smart Speed System, Fusion Equipment, AUS) | 0-5m performance ↓ MD (m·s^-1^): -0.26; 95% CI [-0.4, -0.12]; % Change -5.26%;  SMD: -0.95; 95% CI [-1.57, -0.33] Weight 2.38%; Inference - Large  0-10m performance ↓ MD (m·s^-1^): -0.2; 95% CI [-0.39, -0.01]; % Change -3.48%;  SMD: -0.51; 95% CI [-1.03, 0.01] Weight 1.14%; Inference - Moderate  0-20m performance ↓ MD (m·s^-1^): -0.17; 95% CI [-0.27, -0.07]; % Change -2.54%;  SMD: -0.74; 95% CI [-1.25, -0.23] Weight 0.99%; Inference - Moderate |
| Kobal et al. (2017) 2B (24) | M, n=11, Elite Soccer Players; Age 15.9±1.2 years | Plyometrics training 2d/wk, 6wks, 12 sessions, Pre-season | 2-6 sets of 6 reps/set of vertical and horizontal jumps performed @BW | See Kobal et al. (2017) 2A | 0-5m performance = MD (m·s^-1^): -0.1; 95% CI [-0.2, 0]; % Change -2.09%;  SMD: -0.47; 95% CI [-0.96, 0.02] Weight 2.56%; Inference – Moderate  0-10m performance ↓ MD (m·s^-1^): -0.11; 95% CI [-0.2, -0.02]; % Change -1.94%;  SMD: -0.54; 95% CI [-1.01, -0.06] Weight 1.18%; Inference - Moderate  0-20m performance ↓ MD (m·s^-1^): -0.11; 95% CI [-0.21, -0.01]; % Change -1.67%;  SMD: -0.47; 95% CI [-0.9, -0.04] Weight 1.06%; Inference - Moderate |
| Krommes et al. (2017) A (25) | M, n=9, Elite Soccer Players; Age 23±3.9 years | Strength training 1-3d/wk, 10wks, 27 sessions, Pre-season | Nordic curls 2-3 sets of 5-12 reps/set | N/A  Electronic timing gates (Newtest, Oulu, Finland) | 0-5m performance ↑ MD (s): 0.09; 95% CI [0.04, 0.14]; % Change 11.11%;  SMD: 0.9; 95% CI [0.29, 1.51] Weight 2.39%; Inference - Large  0-10m performance ↑ MD (s): 0.1; 95% CI [0.03, 0.17]; % Change 6.33%;  SMD: 0.7; 95% CI [0.16, 1.25] Weight 1.12%; Inference - Moderate |
| Lago-Fuentes et al. (2018) A (26) | F, n=7 Elite Futsal Players; Age 23.6±4.8 years | Strength training 3d/wk, 6wks, 18 sessions, In-season | 3 sets of 30-50s/set of core strength training (shoulder bridge, side bridge, prone plank and crunch) | Futsal training sessions 5-6/wk + 1 official match/wk  Photoelectric cells (DSD Laser System, León, Spain) | 0-10m performance ↑ MD (s): 0.1; 95% CI [0.06, 0.14]; % Change 5.32%;  SMD: 1.54; 95% CI [0.72, 2.36] Weight 0.92%; Inference - Large |
| Lago-Fuentes et al. (2018) B (26) | F, n=7 Elite Futsal Players; Age 23.8±5.8 years | Strength training 3d/wk, 6wks, 18 sessions, In-season | 3 sets of 30-50s/set of core strength training (shoulder bridge, side bridge, prone plank and crunch) on an unstable surface (Togu® Dyn-Air) | See Lago-Fuentes et al. (2018) A | 0-10m performance ↑ MD (s): 0.09; 95% CI [0.04, 0.14]; % Change 4.86%;  SMD: 1.05; 95% CI [0.36, 1.74] Weight 1.01%; Inference - Large |
| Lockie et al. (2012) 1C (27) | M, n=9, Sub-elite Team sport (i.e., rugby union, rugby league, Australian rules football and soccer Players; Age 23.7±4.7 years | Resistance and plyometrics training 2d/wk, 6wks, 12 sessions, In-season | Moderate-high intensity plyometrics training (2-5 sets of 5-10 foot contacts/set). Exercises consisted of box jumps, double leg hurdle jumps, drop jumps, alternate leg bounds and SL forward hops and 2 gym-based training sessions/wk (not specified) | Normal physical activity generally consisted of 2 field-based and 1 game/wk.  Velocimeter (Onspot, Wollongong, Australia) | 0-5m performance ↑ MD (m·s^1^): 0.21; 95% CI [0.09, 0.33]; % Change 5.56%;  SMD: 0.91; 95% CI [0.3, 1.53] Weight 2.39%; Inference - Large  0-10m performance ↑ MD (m·s^1^): 0.2; 95% CI [0.08, 0.32]; % Change 4.16%;  SMD: 0.85; 95% CI [0.28, 1.42] Weight 1.11%; Inference - Large |
| Lockie et al. (2012) 2B (28) | M, n=6, Sub-elite Soccer, Rugby and Aussie Rules Players; Age 23.1±4.2 years | Strength training 2d/wk, 6wks, 12 sessions, In-season | Moderate-high load (75-90% 1RM) moderate volume (3 sets of 4-12 reps/set) LB strength training (squat, step-ups, hip flexions, calf raises) and 2 gym-based training sessions/wk (not specified) | Normal physical activity generally consisted of 2 field-based and 1 game/wk.  Velocimeter (Onspot, Wollongong, Australia) | 0-5m performance ↑ MD (m·s^1^): 0.35; 95% CI [0.26, 0.44]; % Change 9.51%;  SMD: 2.35; 95% CI [1.13, 3.57] Weight 1.54%; Inference - Large  0-10m performance ↑ MD (m·s^1^): 0.33; 95% CI [0.25, 0.41]; % Change 6.99%;  SMD: 2.43; 95% CI [1.24, 3.63] Weight 0.67%; Inference - Large |
| Lockie et al. (2012) 2C (28) | M, n=9, Sub-elite Soccer, Rugby and Aussie Rules Players; Age 23.1±4.2 years | Resistance and plyometrics training 2d/wk, 6wks, 12 sessions, In-season | Moderate-high intensity plyometrics training (2-5 sets of 5-10 foot contacts/set). Exercises consisted of box jumps, double leg hurdle jumps, drop jumps, alternate leg bounds and SL forward hops | See Lockie et al. (2012) 2B | 0-5m performance ↑ MD (m·s^1^): 0.21; 95% CI [0.09, 0.33]; % Change 5.56%;  SMD: 0.91; 95% CI [0.3, 1.53] Weight 2.39%; Inference - Large  0-10m performance ↑ MD (m·s^1^): 0.2; 95% CI [0.08, 0.32]; % Change 4.16%;  SMD: 0.85; 95% CI [0.28, 1.42] Weight 1.11%; Inference - Large |
| Lockie et al. (2014) B (29) | M, n=8, Sub-elite Soccer, Rugby and Aussie Rules Players; Age 21.81±2.59 years | Resistance and plyometrics training 2d/wk, 6wks, 12 sessions, In-season | Moderate-high intensity plyometrics training (2-5 sets of 5-10 foot contacts/set). Exercises consisted of box jumps, double leg hurdle jumps, drop jumps, alternate leg bounds and SL forward hops and 2 gym-based training sessions/wk (not specified) | Normal physical activity generally consisted of 2 field-based and 1 game/wk.  Velocimeter (Onspot, Wollongong, Australia) | 0-5m performance ↑ MD (s): 0.06; 95% CI [0.02, 0.1]; % Change 4.72%;  SMD: 0.86; 95% CI [0.22, 1.5] Weight 2.35%; Inference - Large  0-10m performance ↑ MD (s): 0.09; 95% CI [0.04, 0.14]; % Change 4.5%;  SMD: 0.94; 95% CI [0.32, 1.56] Weight 1.07%; Inference - Large |
| Los Arcos et al. (2014) A (30) | M, n=7, Elite Soccer Players; Age 20.3±1.9 years | Power and plyometrics training 1-2d/wk, 8wks, 11 sessions, Pre-season and in-season | Light load (30-76% Peak power) low volume (2 sets of 5 reps/set) power training (half squat and SL half squat, calf raise) + low-high intensity and low volume (2-3 sets of 4-5 reps/set) vertical orientated plyometric training (loaded CMJs @+5%Bw, vertical jumps, drop jumps and skipping) | Generic endurance training, as well as soccer-specific training. Players played 6 friendly and 3 competitive matches during the 5 preseason and 3 in-season weeks, respectively.  Photocell beams (Newtest, Oulu, Finland) | 0-5m performance = MD (s): 0.01; 95% CI [-0.02, 0.04]; % Change 1.04%;  SMD: 0.22; 95% CI [-0.37, 0.81] Weight 2.42%; Inference - Small  0-20m performance = MD (s): 0.01; 95% CI [-0.04, 0.06]; % Change 0.43%;  SMD: 0.11; 95% CI [-0.4, 0.63] Weight 0.99%; Inference - Trivial |
| Loturco et al. (2013) A (31) | M, n=16, Elite Soccer Players; Age 19.18±0.72 years | Strength and power training 2d/wk, 6wks, 12 sessions, Pre-season | 3wk LB strength mesocycle followed by a 3 wk LB power mesocycle with a reducing jump squat load. wk1-3, 4 sets of 6-8 reps/set @50-80% 1RM of back squat. Wk4-6, 4 sets of 4-6 reps/set @ 30-60% 1RM of jump squat. The load reduced from 60% 1RM to 30% 1RM across the 3 wks. | Training for general development of soccer-specific technical-tactical skills (4 times/wk).  Photocells (Smart Speed; Fusion Equipment) | 0-10m performance ↑ MD (m·s^1^): 0.09; 95% CI [0.04, 0.14]; % Change 5.3%;  SMD: 0.69; 95% CI [0.28, 1.1] Weight 1.22%; Inference - Moderate |
| Loturco et al. (2013) B (31) | M, n=16, Elite Soccer Players; Age 19.11±0.7 years | Strength and power training 2d/wk, 6wks, 12 sessions, Pre-season | See Loturco et al. (2013) A, instead the load increases from 30% 1RM to 60% 1RM across the 3 wks | See Loturco et al. (2013) A | 0-10m performance = MD (m·s^1^): 0.03; 95% CI [-0.01, 0.07]; % Change 1.58%;  SMD: 0.27; 95% CI [-0.1, 0.64] Weight 1.24%; Inference - Small |
| Loturco et al. (2015) 1A (32) | M, n=12, Elite Soccer Players; Age 23.4±3.6 years | Power training 2-3d/wk, 4wks, 10 sessions, Pre-season | Velocity based training performing 6 sets of 4-8 reps/set of jump squats performed @ a load corresponding to the mass at which optimal power is produced (1-1.1* optimal power load) | 3-5 technical/tactical training sessions + 1 friendly game/wk  Photocells (Smart Speed, Fusion Equipment, Brisbane, Queensland, Australia) | 0-5m performance = MD (m·s^1^): -0.07; 95% CI [-0.16, 0.02]; % Change -1.46%;  SMD: -0.36; 95% CI [-0.82, 0.1] Weight 2.6%; Inference - Small  0-10m performance = MD (m·s^1^): -0.03; 95% CI [-0.1, 0.04]; % Change -0.53%;  SMD: -0.17; 95% CI [-0.6, 0.26] Weight 1.21%; Inference - Trivial  0-20m performance = MD (m·s^1^): -0.03; 95% CI [-0.1, 0.04]; % Change -0.45%;  SMD: -0.16; 95% CI [-0.55, 0.24] Weight 1.09%; Inference - Trivial |
| Loturco et al. (2015) 1B (32) | M, n=11, Elite Soccer Players; Age 24.1±5.2 years | Power training 2-3d/wk, 4wks, 10 sessions, Pre-season | Velocity based training performing 6 sets of 4-8 reps/set of half squats performed at a load corresponding to the mass at which optimal power is produced to 1-1.1* optimal power load | See Loturco et al. (2015) 1A | 0-5m performance ↓ MD (m·s^1^): -0.11; 95% CI [-0.21, -0.01]; % Change -2.36%;  SMD: -0.54; 95% CI [-1.03, -0.04] Weight 2.55%; Inference - Moderate  0-10m performance = MD (m·s^1^): -0.06; 95% CI [-0.15, 0.03]; % Change -1.08%;  SMD: -0.3; 95% CI [-0.75, 0.15] Weight 1.19%; Inference - Small  0-20m performance = MD (m·s^1^): -0.05; 95% CI [-0.14, 0.04]; % Change -0.76%;  SMD: -0.22; 95% CI [-0.63, 0.2] Weight 1.07%; Inference - Small |
| Loturco et al. (2015) 2A (33) | M, n=12, Elite Soccer Players; Age 18.2±0.6 years | Plyometrics training 2-5d/wk, 3wks, 11 sessions, Pre-season | 4-6 sets of 8-10 reps/set of moderate-intensity plyometrics (vertical CMJ's) | 4-5 technical/tactical training sessions + 1 friendly game over the 3-week training period  Photocells (Smart Speed, Fusion Equipment, Brisbane, Queensland, Australia) | 0-10m performance = MD (m·s^1^): 0.03; 95% CI [-0.07, 0.13]; % Change 0.52%;  SMD: 0.13; 95% CI [-0.29, 0.56] Weight 1.21%; Inference - Trivial  0-20m performance ↑ MD (m·s^1^): 0.21; 95% CI [0.11, 0.31]; % Change 3.14%;  SMD: 0.82; 95% CI [0.37, 1.27] Weight 1.04%; Inference - Large |
| Loturco et al. (2015) 2B (33) | M, n=12, Elite Soccer Players; Age 18.5±0.8 years | Plyometrics training 2-5d/wk, 3wks, 11 sessions, Pre-season | 4-6 sets of 8-10 reps/set of moderate intensity plyometrics (horizontal CMJ's) | See Loturco et al. (2015) 2A | 0-10m performance ↑ MD (m·s^1^): 0.13; 95% CI [0.04, 0.22]; % Change 2.29%;  SMD: 0.63; 95% CI [0.17, 1.1] Weight 1.18%; Inference - Moderate  0-20m performance = MD (m·s^1^): 0.05; 95% CI [-0.04, 0.14]; % Change 0.75%;  SMD: 0.23; 95% CI [-0.17, 0.63] Weight 1.09%; Inference - Small |
| Loturco et al. (2015) 3A (34) | M, n=12, Elite Soccer Players; Age 18.7±0.5 years | Power training 3d/wk, 6wks, 18 sessions, Pre-season | Velocity based training performing 6 sets of 6 reps/set of band-assisted jumps squat in using a smith machine barbell with no additional load. Using an elastic band system attached to the smith machine bar the bar velocity was increased by 20%. | Technical-tactical training 2 sessions/wk of ball-specific drills (amounting to 120 mins), 2 sessions/wk of small-sided games (amounting to 90 mins), and 1 simulated match/wk (amounting to 80 mins).  Photocells (Smart Speed; Fusion Equipment) | 0-5m performance ↑ MD (m·s^1^): 0.42; 95% CI [0.33, 0.51]; % Change 9.64%;  SMD: 2.15; 95% CI [1.34, 2.96] Weight 2.1%; Inference - Large  0-10m performance ↑ MD (m·s^1^): 0.34; 95% CI [0.25, 0.43]; % Change 6.37%;  SMD: 1.66; 95% CI [1, 2.31] Weight 1.04%; Inference - Large  0-20m performance ↑ MD (m·s^1^): 0.41; 95% CI [0.33, 0.49]; % Change 6.51%;  SMD: 2; 95% CI [1.32, 2.67] Weight 0.85%; Inference - Large |
| Loturco et al. (2015) 3B (34) | M, n=12, Elite Soccer Players; Age 18.4±0.6 years | Power training 3d/wk, 6wks, 18 sessions, Pre-season | Velocity based training performing 6 sets of 6 reps/set of loaded jumps squat in using a smith machine barbell. Using an additional load added to the smith machine bar the bar velocity was reduced by 20% compared to the unloaded reference velocity. | See Loturco et al. (2015) 3A | 0-5m performance ↑ MD (m·s^1^): 0.15; 95% CI [0.02, 0.28]; % Change 3.35%;  SMD: 0.53; 95% CI [0.05, 1] Weight 2.58%; Inference - Moderate  0-10m performance = MD (m·s^1^): 0.08; 95% CI [-0.02, 0.18]; % Change 1.55%;  SMD: 0.33; 95% CI [-0.1, 0.77] Weight 1.2%; Inference - Small  0-20m performance ↑ MD (m·s^1^): 0.13; 95% CI [0.04, 0.22]; % Change 1.99%;  SMD: 0.59; 95% CI [0.17, 1.02] Weight 1.07%; Inference - Moderate |
| Loturco et al. (2016) 1A (35) | M, n=9, Elite Soccer Players; Age 18.4±1.2 years | Power training 2d/wk, 6wks, 12 sessions, Pre-season | Velocity based training performing 6 sets of 4-8 reps/set of jump squats performed @ a load corresponding to the mass at which optimal power is produced (1-1.1* optimal power load) | Small sided game training 6 sessions/wk with a friendly on the Saturday.  Photocells (Smart Speed; Fusion Equipment) | 0-5m performance ↑ MD (m·s^1^): 0.37; 95% CI [0.27, 0.47]; % Change 7.47%;  SMD: 1.83; 95% CI [0.99, 2.67] Weight 2.05%; Inference - Large  0-10m performance ↑ MD (m·s^1^): 0.32; 95% CI [0.22, 0.42]; % Change 5.49%;  SMD: 1.56; 95% CI [0.84, 2.29] Weight 0.99%; Inference - Large  0-20m performance ↑ MD (m·s^1^): 0.24; 95% CI [0.16, 0.32]; % Change 3.55%;  SMD: 1.32; 95% CI [0.7, 1.94] Weight 0.9%; Inference - Large |
| Loturco et al. (2016) 1B (35) | M, n=8, Elite Soccer Players; Age 18.4±1.2 years | Power training 2d/wk, 6wks, 12 sessions, Pre-season | Velocity based training performing 6 sets of 4-8 reps/set of overhead push press performed @ a load corresponding to the mass at which optimal power is produced (1-1.1* optimal power load) | See Loturco et al. (2016) 1B | 0-5m performance = MD (m·s^1^): 0.07; 95% CI [-0.09, 0.23]; % Change 1.36%;  SMD: 0.24; 95% CI [-0.31, 0.8] Weight 2.47%; Inference - Small  0-10m performance = MD (m·s^1^): 0.02; 95% CI [-0.14, 0.18]; % Change 0.34%;  SMD: 0.06; 95% CI [-0.46, 0.58] Weight 1.14%; Inference - Trivial  0-20m performance = MD (m·s^1^): 0; 95% CI [-0.16, 0.16]; % Change 0%;  SMD: 0; 95% CI [-0.48, 0.48] Weight 1.02%; Inference - Trivial |
| Loturco et al. (2016) 2A (36) | M, n=11, Elite Soccer Players; Age 23.9±4.4 years | Strength and power training 3d/wk, 6wks, 18 sessions, Pre-season | 6wk linear periodised programme. Wk1-4 moderate-high intensity (60-90%1RM) moderate to low volume (6 sets of 4-10 reps/set) half-squats. Wk 5-6 light (30%) intensity and moderate volume (6 sets of 6 reps/set) jump squats. | Small sided game training 6 sessions/wk with a friendly on the Saturday.  Photocells (Smart Speed, Fusion Equipment, AUS) | 0-5m performance ↑ MD (m·s^1^): 0.35; 95% CI [0.21, 0.49]; % Change 7.7%;  SMD: 1.18; 95% CI [0.57, 1.78] Weight 2.4%; Inference - Large  0-10m performance ↑ MD (m·s^1^): 0.2; 95% CI [0.09, 0.31]; % Change 3.52%;  SMD: 0.82; 95% CI [0.3, 1.33] Weight 1.15%; Inference - Large  0-20m performance ↑ MD (m·s^1^): 0.17; 95% CI [0.03, 0.31]; % Change 2.61%;  SMD: 0.49; 95% CI [0.05, 0.92] Weight 1.06%; Inference - Moderate |
| Loturco et al. (2016) 2B (36) | M, n=12, Elite Soccer Players; Age 23.1±3.2 years | Power training 3d/wk, 6wks, 18 sessions, Pre-season | Velocity based training performing 6 sets of 6 reps/set of jump squats performed @ a load corresponding to the mass at which optimal power is produced (1-1.1* optimal power load). | See Loturco et al. (2016) 2A | 0-5m performance ↑ MD (m·s^1^): 0.35; 95% CI [0.19, 0.51]; % Change 7.67%;  SMD: 0.96; 95% CI [0.42, 1.5] Weight 2.49%; Inference - Large  0-10m performance ↑ MD (m·s^1^): 0.42; 95% CI [0.33, 0.51]; % Change 7.52%;  SMD: 1.92; 95% CI [1.21, 2.64] Weight 1%; Inference - Large  0-20m performance ↑ MD (m·s^1^): 0.41; 95% CI [0.29, 0.53]; % Change 6.27%;  SMD: 1.37; 95% CI [0.82, 1.91] Weight 0.96%; Inference - Large |
| Loturco et al. (2017) B (37) | M, n=11, Elite Soccer Players; Age 22.2±2.4 years | Power and plyometrics training 2-3d/wk, 5wks, 12 sessions, Pre-season | Velocity based training performing 6 sets of 4-8 reps/set of jump squats performed @ a load corresponding to the mass at which optimal power is produced + 6-8 sets of 6 reps/set of moderate-intensity plyometrics (wk 1-2 horizontal, wk 3-4 mixed, wk 5-6 vertical CMJ's) | Pre-season soccer training 6/week + 2 pre-season friendly games  Photocells (Smart Speed, Fusion Equipment, AUS) | 0-5m performance ↑ MD (s): 0.08; 95% CI [0.07, 0.09]; % Change 9.23%;  SMD: 4; 95% CI [2.6, 5.4] Weight 1.35%; Inference - Large  0-10m performance ↑ MD (s): 0.09; 95% CI [0.08, 0.1]; % Change 5.43%;  SMD: 3; 95% CI [1.96, 4.04] Weight 0.77%; Inference - Large  0-20m performance ↑ MD (s): 0.05; 95% CI [0.03, 0.07]; % Change 1.67%;  SMD: 1; 95% CI [0.5, 1.5] Weight 1%; Inference - Large |
| Loturco et al. (2019) A (38) | M, n=12, Elite Soccer Players; Age 18.3±0.7 years | Power training 3d/wk, 4wks, 12 sessions, Pre-season | Velocity based training at light loads 6 sets of 6 reps/set of jump squats performed @ a load corresponding to 20% lower than the load at which optimal power is produced (0.8-0.9* optimal power load). | Soccer training 6d/wk across 7 training sessions involving small-sided games and technical, tactical training exercises.  Photocells (Smart Speed, Fusion Sport, Brisbane, AUS) | 0-5m performance ↑ MD (m·s^1^): 0.12; 95% CI [0.03, 0.21]; % Change 2.34%;  SMD: 0.57; 95% CI [0.09, 1.05] Weight 2.57%; Inference - Moderate  0-10m performance = MD (m·s^1^): 0.07; 95% CI [-0.03, 0.17]; % Change 1.18%;  SMD: 0.29; 95% CI [-0.14, 0.72] Weight 1.2%; Inference - Small  0-20m performance = MD (m·s^1^): 0; 95% CI [-0.1, 0.1]; % Change 0%;  SMD: 0; 95% CI [-0.39, 0.39] Weight 1.09%; Inference - Trivial |
| Loturco et al. (2019) B (38) | M, n=11, Elite Soccer Players; Age 18.3±0.7 years | Power training 3d/wk, 4wks, 12 sessions, Pre-season | Velocity based training at light loads 6 sets of 6 reps/set of jump squats performed @ a load corresponding to 20% greater than the load at which optimal power is produced (1.2-1.3* optimal power load). | See Loturco et al. (2019) A | 0-5m performance = MD (m·s^1^): 0.1; 95% CI [-0.05, 0.25]; % Change 1.99%;  SMD: 0.32; 95% CI [-0.16, 0.8] Weight 2.58%; Inference - Small  0-10m performance = MD (m·s^1^): 0.06; 95% CI [-0.05, 0.17]; % Change 1.02%;  SMD: 0.24; 95% CI [-0.21, 0.68] Weight 1.19%; Inference - Small  0-20m performance = MD (m·s^1^): 0.04; 95% CI [-0.06, 0.14]; % Change 0.59%;  SMD: 0.16; 95% CI [-0.26, 0.57] Weight 1.08%; Inference - Trivial |
| Manolopoulos et al. (2004) A (39) | M, n=8, Sub-elite Soccer Players; Age 21.1±1.3 years | Strength training 3d/wk, 8wks, 24 sessions, Pre-season | LB strength training using machines (leg extension, leg curl, adductor machine, calf machine, leg press). Progressing from high volume (20 reps/set) to low volume high-intensity training (1-5 reps/set) | Both groups followed their standard eight-week soccer training program  Photocell fences (Autonics Beam Sensor BL5M-MFR | 0-10m performance = MD (s): 0; 95% CI [-0.03, 0.03]; % Change 0%;  SMD: 0; 95% CI [-0.52, 0.52] Weight 1.14%; Inference - Trivial |
| Manouras et al. (2016) A (40) | M, n=10, Sub-elite Soccer Players; Age 19.1±5.75 years | Plyometrics training 1d/wk, 8wks, 8 sessions, In-season | Moderate-high intensity horizontal plyometric training (ankle jumps, long jumps, obstacle jumps and drop jumps) for 3-5 sets of 4-10 foot contacts/set. | Soccer training 3x/wk and one official game/wk  Electronic timing lights (Newtest PowerTimer, Newtest Oy, Oulu, Finland) | 0-10m performance ↑ MD (s): 0.06; 95% CI [0.03, 0.09]; % Change 3.41%;  SMD: 0.94; 95% CI [0.38, 1.5] Weight 1.12%; Inference - Large |
| Manouras et al. (2016) B (40) | M, n=10, Sub-elite Soccer Players; Age 20.75±6.14 years | Plyometrics training 1d/wk, 8wks, 8 sessions, In-season | Moderate-high intensity horizontal plyometric training (ankle jumps, CMJs, obstacle jumps and drop jumps) for 3-5 sets of 4-10 foot contacts/set. | See Manouras et al. (2016) A | 0-10m performance ↑ MD (s): 0.05; 95% CI [0.02, 0.08]; % Change 2.75%;  SMD: 0.67; 95% CI [0.15, 1.18] Weight 1.15%; Inference - Moderate |
| McMaster et al. (2014) A (41) | M, n=6, Elite Rugby union Players; Age 20.9±1.6 years | Strength and power training 4d/wk, 5wks, 20 sessions, Pre-season | 4d complex training wk Monday: CMJs, countermovement bench throws, power cleans, 1 arm jammer press, 1 arm DB snatch. Tuesday: bench press, concentric-only bench throws, weighted chin ups, high pulls, 1 arm DB floor press, 1 arm DB rows. Thursday: back squat, ¼ squat jumps, Bulgarian split squats and (off-squat rack) and speed lunge (elevated landing) Friday: incline DB press, alternate arm DB bench press, box squat, hip thrusts and calf raises). Strength exercises (4-8 sets of 2-6 reps/set @ 85-99% 1RM) and Heavy ballistic exercises (4-5 sets of 2-4 reps @ 60-75% 1RM) | N/A  Dual beam infrared timing lights (Swift Performance, Lismore, Australia) | 0-10m performance = MD (s): 0.02; 95% CI [-0.01, 0.05]; % Change 1.16%;  SMD: 0.35; 95% CI [-0.26, 0.97] Weight 1.07%; Inference - Small  0-20m performance = MD (s): 0.03; 95% CI [-0.01, 0.07]; % Change 1%;  SMD: 0.39; 95% CI [-0.18, 0.97] Weight 0.94%; Inference - Small |
| McMaster et al. (2014) B (41) | M, n=8, Elite Rugby union Players; Age 20.9±1.6 years | Strength and power training 4d/wk, 5wks, 20 sessions, Pre-season | Four-day complex training week Monday: CMJs, countermovement bench throws, power cleans, 1 arm jammer press, 1 arm DB snatch. Tuesday: bench press, concentric-only bench throws, weighted chin ups, high pulls, 1 arm DB floor press, 1 arm DB rows. Thursday: back squat, ¼ squat jumps, Bulgarian split squats and (off-squat rack) and speed lunge (elevated landing) Friday: incline DB press, Alternate arm DB bench press, box squat, hip thrusts and calf raises). Strength exercises (4-8 sets of 2-6 reps/set @ 85-99% 1RM) and light ballistic exercises (6-8 sets of 5-8 reps/set @ 15-30%1RM) | See McMaster et al. (2014) A | 0-10m performance ↑ MD (s): 0.03; 95% CI [0.01, 0.05]; % Change 1.73%;  SMD: 0.64; 95% CI [0.07, 1.21] Weight 1.11%; Inference - Moderate  0-20m performance = MD (s): 0.03; 95% CI [-0.01, 0.07]; % Change 1%;  SMD: 0.35; 95% CI [-0.15, 0.84] Weight 1.01%; Inference - Small |
| Meylan and Malatesta (2009) (42) | M, n=14, Soccer Players; Age 13.3±0.6 years | Plyometrics training 2d/wk, 8wks, 16 sessions, In-season | Low-high intensity (2-4 sets of 6-12 foot contacts/set) plyometric training (jumping, hurdling, bouncing, skipping, and footwork). 1d/wk had a vertical focus and 1d/wk a horizontal focus. | Soccer training 2d/wk  Infrared photoelectric cells (Globus Italia, Codogne, Italy) | 0-10m performance = MD (s): 0.05; 95% CI [0, 0.1]; % Change 2.49%;  SMD: 0.44; 95% CI [-0.02, 0.9] Weight 2.48%; Inference - Moderate |
| Nakamura et al. (2012) (43) | M, n=11, Elite Soccer Players; Age 22.7±2.4 years | Plyometrics training 2d/wk, 3wks, 6 sessions, Off-season | Mod-high intensity (6 sets of 10-11 reps/set) plyometrics training (60 cm bar jumps and bounding) | Off-season - no extra training  Photocell gates (Wireless Sprint System, Brower Timing System, Draper, UT, USA). | 0-5m performance ↓ MD (s): -0.07; 95% CI [-0.11, -0.03]; % Change -6.67%;  SMD: -0.92; 95% CI [-1.48, -0.37] Weight 2.47%; Inference - Large  0-10m performance ↓ MD (s): -0.08; 95% CI [-0.12, -0.04]; % Change -4.42%;  SMD: -1; 95% CI [-1.54, -0.46] Weight 1.13%; Inference - Large  0-20m performance ↓ MD (s): -0.1; 95% CI [-0.15, -0.05]; % Change -3.19%;  SMD: -0.8; 95% CI [-1.27, -0.33] Weight 1.03%; Inference - Moderate |
| Negra et al. (2016) A (44) | M, n=13, Elite Soccer Players; Age 12.8±0.25 years | Power training 2d/wk, 12wks, 24 sessions, In-season | Low-moderate load (40-60%) high volume high-velocity power training 4 sets of 8–12 rep/set of half squats AND abdominal curl and back extension exercises 6 sets of 15 reps/set. | Regular soccer training with 3 sessions/wk and 1 game/wk.  Single-beam photoelectric gates | 0-5m performance ↑ MD (s): 0.08; 95% CI [0.04, 0.12]; % Change 7.41%;  SMD: 0.97; 95% CI [0.45, 1.48] Weight 2.52%; Inference - Large  0-10m performance ↑ MD (s): 0.15; 95% CI [0.1, 0.2]; % Change 7.89%;  SMD: 1.34; 95% CI [0.78, 1.91] Weight 1.11%; Inference - Large  0-20m performance ↑ MD (s): 0.17; 95% CI  [0.09, 0.25]; % Change 5.01%;  SMD: 0.79; 95% CI [0.36, 1.22] Weight 1.06%; Inference - Moderate |
| Negra et al. (2019) A (45) | M, n=16, Soccer Players; Age 13±0.5 years | Plyometrics training 2d/wk, 8wks, 16 sessions, In-season | 4-6 sets of plyometric training 6-10 foot contacts/set of Bilateral forward ankle hops (hurdle height: 20 cm) and cyclical CMJs | Soccer training 5 sessions/wk  Infrared photocell system (Microgate, Bolzano, Italy) | 0-5m performance ↑ MD (s): 0.1; 95% CI [0.06, 0.14]; % Change 9.09%;  SMD: 1; 95% CI [0.53, 1.47] Weight 2.58%; Inference - Large  0-10m performance ↑ MD (s): 0.1; 95% CI [0.03, 0.17]; % Change 5%;  SMD: 0.51; 95% CI [0.12, 0.9] Weight 1.23%; Inference - Moderate  0-20m performance = MD (s): 0.1; 95% CI [0, 0.2]; % Change 2.78%;  SMD: 0.33; 95% CI [-0.02, 0.68] Weight 1.13%; Inference - Small |
| Negra et al. (2019) B (45) | M, n=13, Soccer Players; Age 13±0.7 years | Plyometrics training 2d/wk, 8wks, 16 sessions, In-season | 4-6 sets of plyometric training 6-10 foot contacts/set of bilateral forward ankle hops (hurdle height: 20 cm) and cyclical CMJs with a weighted vest (8%BM) | Soccer training 5 sessions/wk  Infrared photocell system (Microgate, Bolzano, Italy) | 0-5m performance ↑ MD (s): 0.1; 95% CI [0.06, 0.14]; % Change 8.33%;  SMD: 1; 95% CI [0.48, 1.52] Weight 2.51%; Inference - Large  0-10m performance ↑ MD (s): 0.2; 95% CI [0.16, 0.24]; % Change 10%;  SMD: 2; 95% CI [1.3, 2.7] Weight 1%; Inference - Large  0-20m performance ↑ MD (s): 0.2; 95% CI [0.12, 0.28]; % Change 5.56%;  SMD: 1; 95% CI [0.54, 1.46] Weight 1.04%; Inference - Large |
| Pienaar and Coetzee (2013) A (46) | M, n=15, Elite Rugby league Players; Age 17±1 years | Strength and plyometrics training 3d/wk, 4wks, 12 sessions, Pre-season | UB and LB plyometrics training + "core" strength exercises. Resistance training sessions consisted of 12–16 medium-high intensity resistance exercises (70–85% of the 1RM) | Field sessions 5 d/wk and resistance training sessions 3 times/wk that each lasted for ~2 hrs/session.  Intermediate beam electronic timing gates (Brower Timing Systems, Draper, UT, USA | 0-5m performance ↑ MD (s): 0.08; 95% CI [0.03, 0.13]; % Change 7.02%;  SMD: 0.54; 95% CI [0.16, 0.92] Weight 2.69%; Inference - Moderate  0-10m performance ↑ MD (s): 0.06; 95% CI [0, 0.12]; % Change 3.13%;  SMD: 0.36; 95% CI [0.01, 0.71] Weight 1.26%; Inference - Small  0-20m performance ↑ MD (s): 0.09; 95% CI [0.02, 0.16]; % Change 2.77%;  SMD: 0.38; 95% CI [0.05, 0.7] Weight 1.14%; Inference - Small |
| Pienaar and Coetzee (2013) B (46) | M, n=12, Elite Rugby league Players; Age 17±1 years | Strength training 3d/wk, 4wks, 12 sessions, Pre-season | Resistance training sessions consisted of 12–16 medium-high intensity resistance exercises (70–85% of the 1RM) | See Pienaar and Coetzee (2013) A | 0-5m performance = MD (s): 0; 95% CI [-0.07, 0.07]; % Change 0%;  SMD: 0; 95% CI [-0.39, 0.39] Weight 2.69%; Inference - Trivial  0-10m performance = MD (s): 0; 95% CI [-0.07, 0.07]; % Change 0%;  SMD: 0; 95% CI [-0.37, 0.37] Weight 1.25%; Inference - Trivial  0-20m performance = MD (s): -0.04; 95% CI [-0.12, 0.04]; % Change -1.23%; SMD: -0.18; 95% CI [-0.52, 0.16] Weight 1.13%; Inference - Trivial |
| Ramirez-Campillo et al. (2013) A (47) | M, n=19, Rugby Players; Age 18.94±0.38 years | Plyometrics training 2d/wk, 7wks, 14 sessions, Phase not reported | Mod-high intensity low volume (6 sets of 10 reps/set) plyometrics (20-60 cm drop jumps) performed on a soft surface | Photoelectric cell (Globus Tester). | 0-20m performance ↑ MD (s): 0.08; 95% CI [0.02, 0.14]; % Change 2.06%;  SMD: 0.64; 95% CI [0.14, 1.13] Weight 1.01%; Inference - Moderate |
| Ramirez-Campillo et al. (2013) B (47) | M, n=16, Rugby Players; Age 18.94±0.42 years | Plyometrics training 2d/wk, 7wks, 14 sessions, Phase not reported | Mod-high low volume (6 sets of 10 reps/set) (20-60 cm drop jumps) plyometrics performed on a hard surface | See Ramirez-Campillo et al. (2013) A | 0-20m performance = MD (s): 0.02; 95% CI [-0.05, 0.09]; % Change 0.41%;  SMD: 0.14; 95% CI [-0.34, 0.62] Weight 1.02%; Inference - Trivial |
| Ramirez-Campillo et al. (2013) C (47) | M, n=9, Sub-elite Soccer Players; Age 16.89±0.85 years | Plyometrics training 2d/wk, 7wks, 14 sessions, Phase not reported | Mod-high low volume (6 sets of 10 reps/set) (20-60 cm drop jumps) plyometrics performed on a soft surface | See Ramirez-Campillo et al. (2013) A | 0-20m performance = MD (s): 0.04; 95% CI [-0.04, 0.12]; % Change 1.05%;  SMD: 0.25; 95% CI [-0.27, 0.77] Weight 0.98%; Inference - Small |
| Ramirez-Campillo et al. (2014) 1A (48) | M, n=8, Sub-elite Soccer Players; Age 16.89±0.85 years | Plyometrics training 2d/wk, 7wks, 14 sessions, In-season | Mod-high low volume (6 sets of 10 reps/set) (20-60 cm drop jumps) with 30s inter-set rest on a grass pitch surface | Soccer training 2 sessions/ wk of 90 min, consisting of 30 min of technical-tactical exercises, 30 min of small-sided games, and 30 min of simulated competitive games.  Single beam infrared reds photoelectric cells (Globus Italia, Codogne, Italy) | 0-20m performance = MD (s): 0; 95% CI [-0.18, 0.18]; % Change 0%;  SMD: 0; 95% CI [-0.38, 0.38] Weight 1.1%; Inference - Trivial |
| Ramirez-Campillo et al. (2014) 1B (48) | M, n=7, Sub-elite Soccer Players; Age 16.89±0.85 years | Plyometrics training 2d/wk, 7wks, 14 sessions, In-season | Mod-high low volume (6 sets of 10 reps/set) (20-60 cm drop jumps) with 60s inter-set rest on a grass pitch surface | See Ramirez-Campillo et al. (2014) 1A | 0-20m performance = MD (s): 0; 95% CI [-0.1, 0.1]; % Change 0%;  SMD: 0; 95% CI [-0.36, 0.36] Weight 1.11%; Inference - Trivial |
| Ramirez-Campillo et al. (2014) 1C (48) | M, n=13, Sub-elite Soccer Players; Age 10.4±2 years | Plyometrics training 2d/wk, 7wks, 14 sessions, In-season | Mod-high low volume (6 sets of 10 reps/set) (20-60 cm drop jumps) with 120s inter-set rest on a grass pitch surface | See Ramirez-Campillo et al. (2014) 1A | 0-20m performance = MD (s): -0.1; 95% CI [-0.26, 0.06]; % Change -2.27%;  SMD: -0.25; 95% CI [-0.65, 0.15] Weight 1.09%; Inference - Small |
| Ramirez-Campillo et al. (2014) 2A (49) | M, n=14, Sub-elite Soccer Players; Age 10.4±2.3 years | Plyometrics training 2d/wk, 7wks, 14 sessions, Phase not reported | Mod-high intensity low volume (6 sets of 10 reps/set) (20-60 cm drop jumps) on a grass pitch surface | Technical/ tactical drills, small-sided games, simulated competitive games and 1 d/wk injury prevention session [not specified].  Single beam infrared reds photoelectric cells (Globus Italia, Codogne, Italy). | 0-20m performance = MD (s): 0.02; 95% CI [-0.1, 0.14]; % Change 0.47%;  SMD: 0.04; 95% CI [-0.18, 0.26] Weight 1.21%; Inference - Trivial |
| Ramirez-Campillo et al. (2015) 1A (50) | M, n=12, Sub-elite Soccer Players; Age 10.3±2.3 years | Plyometrics training 2d/wk, 6wks, 12 sessions, In-season | Mod-high intensity low volume (3 sets of 5-10 reps/set) vertical plyometrics (bilateral and unilateral cyclic and acyclic jumps) on a grass pitch surface | Soccer training 3 sessions/wk  Single beam infrared photoelectric cells (Globus Italy) | 0-20m performance = MD (s): 0.11; 95% CI [-0.01, 0.23]; % Change 3.28%;  SMD: 0.38; 95% CI [-0.07, 0.82] Weight 1.05%; Inference - Small |
| Ramirez-Campillo et al. (2015) 1B (50) | M, n=38, Soccer Players; Age 17.1±0.5 years | Plyometrics training 2d/wk, 6wks, 12 sessions, In-season | Mod-high intensity low volume (3 sets of 5-10 reps/set) horizontal plyometrics (bilateral and unilateral cyclic and acyclic jumps) on a grass pitch surface | See Ramirez-Campillo et al. (2015) 1A | 0-20m performance ↑ MD (s): 0.18; 95% CI [0.05, 0.31]; % Change 5.56%;  SMD: 0.58; 95% CI [0.12, 1.05] Weight 1.03%; Inference - Moderate |
| Ramirez-Campillo et al. (2015) 1C (50) | M, n=10, Soccer Players; Age 11.6±1.4 years | Plyometrics training 2d/wk, 6wks, 12 sessions, In-season | Mod-high intensity low volume (2 sets of 5-10 reps/set) vertical + horizontal plyometrics (bilateral and unilateral cyclic and acyclic jumps) | See Ramirez-Campillo et al. (2015) 1A | 0-20m performance ↑ MD (s): 0.21; 95% CI [0.13, 0.29]; % Change 6.36%;  SMD: 1.07; 95% CI [0.54, 1.61] Weight 0.97%; Inference - Large |
| Ramirez-Campillo et al. (2015) 2A (51) | M, n=10, Soccer Players; Age 11.4±1.9 years | Plyometrics training 2d/wk, 6wks, 12 sessions, Phase not reported | Mod-high intensity low volume (3 sets of 5-10 reps/set) horizontal plyometrics (bilateral and unilateral cyclic and acyclic jumps) on a grass pitch surface | Soccer training 3 sessions/wk and 1 game/wk  Single beam infrared photoelectric cells (Globus Italy) | 0-20m performance ↑ MD (s): 0.13; 95% CI [0.02, 0.24]; % Change 4.1%;  SMD: 0.46; 95% CI [0.05, 0.87] Weight 1.08%; Inference - Moderate |
| Ramirez-Campillo et al. (2015) 2B (51) | M, n=10, Soccer Players; Age 11.2±2.3 years | Plyometrics training 2d/wk, 6wks, 12 sessions, Phase not reported | Mod-high intensity low volume (3 sets of 5-10 reps/set) horizontal plyometrics (bilateral and unilateral cyclic and acyclic jumps) on a grass pitch surface | See Ramirez-Campillo et al. (2015) 2A | 0-20m performance ↑ MD (s): 0.19; 95% CI [0.04, 0.34]; % Change 5.74%;  SMD: 0.44; 95% CI [0.09, 0.8] Weight 1.12%; Inference - Moderate |
| Ramirez-Campillo et al. (2015) 2C (51) | M, n=12, Sub-elite Soccer Players; Age 11±2 years | Plyometrics training 2d/wk, 6wks, 12 sessions, Phase not reported | Mod-high intensity low volume (3 sets of 5-10 reps/set) horizontal plyometrics (bilateral and unilateral cyclic and acyclic jumps) on a grass pitch surface | See Ramirez-Campillo et al. (2015) 2A | 0-20m performance ↑ MD (s): 0.24; 95% CI [0.09, 0.39]; % Change 7.36%;  SMD: 0.61; 95% CI [0.19, 1.04] Weight 1.06%; Inference - Moderate |
| Ramirez-Campillo et al. (2016) A (52) | M, n=16, Sub-elite Soccer Players; Age 11.6±1.7 years | Plyometrics training 2d/wk, 6wks, 12 sessions, In-season | Cyclic and acyclic unilateral and bilateral horizontal and vertical plyometrics on a grass pitch surface | Single-beam infrared photoelectric cells (Globus Italia, Codogne, Italy) | 0-20m performance ↑ MD (s): 0.13; 95% CI [0.01, 0.25]; % Change 3.48%;  SMD: 0.46; 95% CI [0, 0.91] Weight 1.04%; Inference - Moderate |
| Ramirez-Campillo et al. (2018) 1A (53) | M, n=12, Sub-elite Soccer Players; Age 11.6±2.7 years | Plyometrics training 2d/wk, 7wks, 14 sessions, In-season | Moderate intensity low volume (12 sets of 10 reps/set) plyometrics (30 cm drop jumps) from a fixed height on a grass pitch surface | 4 training sessions/wk (technical-tactical, small-sided and simulated games, and injury prevention [not specified]) plus a competitive match/wk  Single-beam timing gates (Brower Timing System, Salt Lake City, UT, USA) | 0-20m performance = MD (s): -0.07; 95% CI [-0.21, 0.07]; % Change -1.58%;  SMD: -0.14; 95% CI [-0.41, 0.13] Weight 1.18%; Inference - Trivial |
| Ramirez-Campillo et al. (2018) 1B (53) | F, n=10 Sub-elite Soccer Players; Age 22.9±1.7 years | Plyometrics training 2d/wk, 7wks, 14 sessions, In-season | Mod-high intensity low volume (12 sets of 10 reps/set) (drop jumps corresponding to optimal reactive strength index) on a grass pitch surface | See Ramirez-Campillo et al. (2018) 1B | 0-20m performance = MD (s): 0.16; 95% CI [-0.01, 0.33]; % Change 3.82%;  SMD: 0.27; 95% CI [-0.02, 0.55] Weight 1.17%; Inference - Small |
| Ramirez-Campillo et al. (2018) 2A (54) | M, n=25, Elite Soccer Players; Age 14.1±2.2 years | Plyometrics training 2d/wk, 7wks, 14 sessions, In-season | Moderate-high intensity training before technical tactical training (wall jump, squat jump, tuck jump, 180 jump, broad jump and hold, bounding in place, bounding for distance, SL hop and hold, jump, jump, jump, vertical jump, lunge jump, crossover hop hop stick, SL clock hop drop jumps) low volume (1 set of 5-16 reps/set) on a grass pitch surface. | N/A  Timing gates (Brower Timing System, Salt Lake City, UT) | 0-20m performance ↑ MD (s): 0.29; 95% CI [0.16, 0.42]; % Change 8.06%;  SMD: 0.88; 95% CI [0.42, 1.35] Weight 1.03%; Inference - Large |
| Ramirez-Campillo et al. (2018) 2B (54) | M, n=24, Elite Soccer Players; Age 14.2±2.2 years | Plyometrics training 2d/wk, 7wks, 14 sessions, In-season | Moderate-high intensity training after technical tactical training (wall jump, squat jump, tuck jump, 180 jump, broad jump and hold, bounding in place, bounding for distance, SL hop and hold, jump, jump, jump, vertical jump, lunge jump, crossover hop hop stick, SL clock hop drop jumps) low volume (1 set of 5-16 reps/set) on a grass pitch surface. | See Ramirez-Campillo et al. (2018) 2A | 0-20m performance = MD (s): 0.02; 95% CI [-0.07, 0.11]; % Change 0.52%;  SMD: 0.08; 95% CI [-0.28, 0.45] Weight 1.11%; Inference - Trivial |
| Ramirez-Campillo et al. (2018) 3A (55) | M, n=12, Elite Soccer Players; Age 13.9±1.9 years | Plyometrics training 2d/wk, 7wks, 14 sessions, Phase not reported | Mod-high-intensity low volume (6 sets of 10 reps/set) plyometrics (20-60 cm drop jumps) on a grass pitch surface. | Technical/ tactical drills, small-sided games, and simulated competitive games and 1 d/wk injury prevention session Single beam infrared reds  photoelectric cells (Globus Italia, Codogne, Italy). | 0-20m performance = MD (s): 0.02; 95% CI [-0.1, 0.14]; % Change 0.47%;  SMD: 0.04; 95% CI [-0.18, 0.26] Weight 1.21%; Inference - Trivial |
| Ramirez-Campillo et al. (2018) 4A (56) | M, n=14, Elite Soccer Players; Age 13.1±1.7 years | Plyometrics training 1d/wk, 8wks, 16 sessions, In-season | Mod-high intensity (1 set of 7-14 reps/set) vertical + horizontal plyometrics (drop jumps, standing long jumps, unilateral countermovement jumps, 180 degrees jumps and repeated countermovement jumps) | Regular soccer training (i.e., mainly technical-tactical drills, small-sided and simulated games, and injury prevention drills). 3 times/wk 120-min soccer-training sessions and a competitive match/wk  Single-beam timing gates (Brower® Timing System, Salt Lake City, Utha, USA). | 0-20m performance ↑ MD (s): 0.27; 95% CI [0.22, 0.32]; % Change 8.97%;  SMD: 2.7; 95% CI [1.67, 3.73] Weight 0.59%; Inference - Large |
| Ramirez-Campillo et al. (2018) 4B (56) | M, n=38, Soccer Players; Age 16.9±0.7 years | Plyometrics training 2d/wk, 8wks, 16 sessions, In-season | Mod-high intensity (1 set of 7-14 reps/set) vertical + horizontal plyometrics (drop jumps, standing long jumps, unilateral countermovement jumps, 180 degrees jumps and repeated countermovement jumps) | See Ramirez-Campillo et al. (2018) 4A | 0-20m performance ↑ MD (s): 0.33; 95% CI [0.28, 0.38]; % Change 10.65%;  SMD: 3.3; 95% CI [2.08, 4.52] Weight 0.49%; Inference - Large |
| Ramirez-Campillo et al. (2019) A (57) | F, n=8 Sub-elite Soccer Players; Age 13±3 years | Plyometrics training 2d/wk, 7wks, 14 sessions, In-season | 3 sets of 10 foot contacts of plyometric training using drop jumps at 20 and 40cm drop heights | Soccer training 2d/wk + 1 official game/wk  Single-beam infrared reds photoelectric cells (Globus, Codogné, Italy) | 0-20m performance = MD (s): 0.04; 95% CI [-0.13, 0.21]; % Change 0.95%;  SMD: 0.07; 95% CI [-0.24, 0.39] Weight 1.15%; Inference - Trivial |
| Randell et al. (2011) A (58) | F, n=8 Sub-elite Soccer Players; Age 13.2±1.8 years | Strength and power training 3d/wk, 6wks, 18 sessions, Pre-season | Low-high load FB strength/ strength-speed exercises for 3-5 sets of 6-10 reps/set @RM load. Squat jumps where performed 1st on Wednesdays and Friday @40kg load (3 sets of 3 reps/set) with instantaneous performance feedback (peak velocity) provided after each repetition of squat jump. Strength exercises consisted of horizontal and vertical pushing exercises, squatting and deadlift patterns as well as injury prevention exercises. | Other conditioning sessions involved an energetic and skills focus  Wireless timing lights | 0-10m performance = MD (s): 0.01; 95% CI [-0.02, 0.04]; % Change 0.58%;  SMD: 0.21; 95% CI [-0.35, 0.77] Weight 1.11%; Inference – Small  0-20m performance = MD (s): 0.03; 95% CI [0, 0.06]; % Change 1%;  SMD: 0.5; 95% CI [-0.04, 1.04] Weight 0.96%; Inference - Moderate |
| Randell et al. (2011) B (58) | M, n=19, Sub-elite Soccer Players; Age 13.2±1.8 years | Strength and power training 3d/wk, 6wks, 18 sessions, Pre-season | Low-high load FB strength/ strength-speed exercises for 3-5 sets of 6-10 reps/set @RM load. Squat jumps where performed 1st on Wednesdays and Friday @40kg load (3 sets of 3 reps/set) with no instantaneous performance feedback provided. Strength exercises consisted of horizontal and vertical pushing exercises, squatting and deadlift patterns as well as injury prevention exercises. | See Randell et al. (2011) A | 0-10m performance = MD (s): 0; 95% CI [-0.06, 0.06]; % Change 0%;  SMD: 0; 95% CI [-0.6, 0.6] Weight 1.08%; Inference - Trivial  0-20m performance = MD (s): 0; 95% CI [-0.09, 0.09]; % Change 0%;  SMD: 0; 95% CI [-0.55, 0.55] Weight 0.96%; Inference - Trivial |
| Rimmer and Sleivert (2000) A (59) | M, n=7, Elite Rugby Players; Age 25.7±3.6 years | Plyometrics training 1-2d/wk, 8wks, 15 sessions, Phase not reported | High-intensity plyometric training high volume (2-5 sets of 5-12 foot contacts) of bilateral and unilateral tuck jumps, speed jumps, hops, bounds and stair bounds on a grass surface. | N/A  Digital timer (University of Otago, Dunedin, New Zealand) | 0-10m performance ↑ MD (s): 0.05; 95% CI [0.01, 0.09]; % Change 2.62%;  SMD: 0.54; 95% CI [0.04, 1.03] Weight 1.16%; Inference - Moderate  0-20m performance = MD (s): 0.01; 95% CI [-0.01, 0.03]; % Change 0.78%;  SMD: 0.21; 95% CI [-0.23, 0.64] Weight 1.06%; Inference - Small |
| Rodriguez-Rosell et al. (2017) 2A (60) | M, n=6, Elite Rugby Players; Age 24.2±2.5 years | Power training 2d/wk, 6wks, 12 sessions, In-season | Velocity based training using light-moderate loads (45-60% 1RM) and low – moderate volume (2-3 sets and 4-8 reps/set) power training (full squat) | 4 field soccer training sessions plus 1 friendly match/wk. Each training session lasted on average 2 hrs and comprised various skill activities at different intensities, small-sided games, and finally 20 mins of continuous play or high-intensity interval training  Infrared timing system (Optojump, Microgate, Bolzano, Italy) | 0-10m performance ↑ MD (s): 0.05; 95% CI [0.02, 0.08]; % Change 2.91%;  SMD: 0.67; 95% CI [0.16, 1.19] Weight 1.15%; Inference - Moderate  0-20m performance ↑ MD (s): 0.05; 95% CI [0.01, 0.09]; % Change 1.67%;  SMD: 0.48; 95% CI [0.03, 0.94] Weight 1.04%; Inference - Moderate |
| Ronnestad et al. (2008) A (61) | M, n=10, Sub-elite Rugby + touch rugby Players; Age 24.4±4 years | Strength training 2d/wk, 7wks, 14 sessions, Phase not reported | High loads (85-90% 1RM) and low volumes (3-5 sets and 4-6 reps/set) of half squats and hip flexions. | 6-8 soccer training sessions/wk  Photocells (JBL Systems, Oslo, Norway) | 0-10m performance ↑ MD (s): 0.03; 95% CI [0.01, 0.05]; % Change 1.7%;  SMD: 1.08; 95% CI [0.32, 1.83] Weight 0.97%; Inference - Large |
| Ronnestad et al. (2008) B (61) | M, n=10, Elite Soccer Players; Age 24.5±3.4 years | Strength and plyometrics training 2d/wk, 7wks, 14 sessions, Phase not reported | High loads (85-90% 1RM) and low volumes (3-5 sets and 4-6 repetitions/set) of half squats and hip flexions + high-intensity low volume (2-4 sets and 5-10 reps /set) of Plyometrics (bounding hurdle jumps and single leg hops) | See Ronnenstad et al. (2008) A | 0-10m performance ↑ MD (s): 0.02; 95% CI [0.01, 0.03]; % Change 1.15%;  SMD: 1; 95% CI [0.36, 1.64] Weight 1.06%; Inference - Large |
| Sánchez-Sánchez et al. (2015) A (62) | M, n=6, Elite Soccer Players; Age 22±2.5 years | Strength training 2d/wk, 10wks, 20 sessions, In-season | LB resistance machine strength training programme using moderate loads (60-75% 1RM) and high volumes (3-5 sets and 6-12 reps/set) of knee extension, knee flexion, ankle extension while sitting, adductor and abductor muscles of the hip while sitting. | 4 d/wk football training + 1 official match on the weekend  Photoelectric cells “Chrono” Globus Ergo System | 0-10m performance = MD (s): 0.02; 95% CI [-0.02, 0.06]; % Change 1.09%;  SMD: 0.18; 95% CI [-0.15, 0.51] Weight 1.27%; Inference - Trivial  0-20m performance = MD (s): 0.08; 95% CI [0, 0.16]; % Change 2.52%;  SMD: 0.29; 95% CI [-0.02, 0.6] Weight 1.15%; Inference - Small |
| Scott et al. (2017) A (63) | M, n=8, Elite Soccer Players; Age 23±2 years | Strength training 2-3d/wk, 5wks, 14 sessions, Pre-season | Moderate-heavy loads (75-85% 1RM) and high volumes (3-4 sets of 6-10 repetitions/set) of UB and LB strength training + low volume (3-4 sets and 6 reps /set) of power training (Jump shrug, broad jumps) + supplementary very low loads (20-30% 1RM) high volume (4 sets of 15-30 reps/set) blood flow restriction training (squats) | Football training was typified by tactical, technical, and match simulation drills involving running, sprinting, change of direction, jumping, collisions, and kicking.  Wireless electronic timing gate system (SpeedLight; Swift Performance, Brisbane, Australia) | 0-10m performance ↓ MD (s): -0.04; 95% CI [-0.07, -0.01]; % Change -2.25%;  SMD: -0.6; 95% CI [-1.11, -0.1] Weight 1.15%; Inference - Moderate  0-20m performance = MD (s): -0.03; 95% CI [-0.07, 0.01]; % Change -0.99%;  SMD: -0.36; 95% CI [-0.8, 0.09] Weight 1.05%; Inference - Small |
| Scott et al. (2017) B (63) | M, n=20, Elite Soccer Players; Age 14.8±0.41 years | Strength training 2-3d/wk, 5wks, 14 sessions, Pre-season | Moderate-heavy loads (75-85% 1RM) and moderate volumes (3-4 sets of 6-10 repetitions/set) of UB and LB strength training + low volume (3-4 sets of 6 reps /set) of power training (Jump shrug, broad jumps) + supplementary very light loads (20-30% 1RM) high volume (4 sets of 15-30 reps/set) non blood flow restriction training (squats) | See Scott et al. (2017) A | 0-10m performance ↓ MD (s): -0.04; 95% CI [-0.08, 0]; % Change -2.26%;  SMD: -0.57; 95% CI [-1.13, -0.01] Weight 1.11%; Inference - Moderate  0-20m performance = MD (s): -0.02; 95% CI [-0.08, 0.04]; % Change -0.66%;  SMD: -0.16; 95% CI [-0.65, 0.32] Weight 1.02%; Inference - Trivial |
| Shalfawi et al. (2012) B (64) | M, n=10, Elite Ausi football Players; Age 19.8±1.5 years | Strength training 2d/wk, 8wks, 16 sessions, Phase not reported | Resistance training nordic curl, balance training (ankle strength on balance board), sit-ups, the plank, push-ups and the alternating back and arm raise 2 times/wk during soccer training (volume/ intensity not provided). | Soccer training 4 times/wk  Newtest Powertimer 300s infrared photocells | 0-20m performance ↑ MD (s): 0.11; 95% CI [0.03, 0.19]; % Change 3.54%;  SMD: 0.69; 95% CI [0.12, 1.26] Weight 0.94%; Inference - Moderate |
| Singh et al. (2014) A (65) | M, n=8, Elite Ausi football Players; Age 19.8±1.5 years | Strength, power and plyometrics training 2 strength training, 1 plyometric d/wk, 6wks, 18 sessions, Pre-season | Heavy loads (85% 1RM) and moderate volume (4 sets of 6 reps/set) of LB power + FB strength training (high pull, jump squat, half back squat, bench press, chin ups) + mod-high intensity plyometric (80-120 foot contacts/ session) training exercises (rebound jumps + drop jumps) | 6-8 soccer training sessions/wk  Stop watch | 0-10m performance ↑ MD (s): 0.08; 95% CI [0.08, 0.08]; % Change 4.44%;  SMD: 8; 95% CI [5.75, 10.25] Weight 0.29%; Inference - Large |
| So¨hnlein et al. (2014) A (66) | M, n=7, Elite Soccer Players; Age 16.3±0.5 years | Plyometrics training 2d/wk, 16wks, 32 sessions, In-season | 2-5 sets of 6-16 reps/set of plyometrics training. Day 1 vertical horizontal focused exercises (2-footed ankle hop forward, hurdle jumps, SL hop forward, and squat jump). Day 2 Lateral focused exercises (lateral bound stabilisation, lateral hurdle jumps, double leg zigzag, and SL hop lateral). 2 (of 8) slow SSC-type, 6 (of 8) fast SSC-type plyometric exercises | 4-5 soccer training session/wk + 1 game/wk  Infrared photoelectric gate (Brower Timing Systems, Draper, UT, USA) | 0-5m performance ↑ MD (s): 0.04; 95% CI [0.02, 0.06]; % Change 3.92%;  SMD: 1; 95% CI [0.45, 1.55] Weight 2.48%; Inference - Large  0-10m performance ↑ MD (s): 0.04; 95% CI [0.01, 0.07]; % Change 2.29%;  SMD: 0.67; 95% CI [0.2, 1.13] Weight 1.18%; Inference - Moderate  0-20m performance ↑ MD (s): 0.1; 95% CI [0.05, 0.15]; % Change 3.28%;  SMD: 0.76; 95% CI [0.31, 1.2] Weight 1.05%; Inference - Moderate |
| Styles et al. (2016) (67) | M, n=14, Elite Soccer Players; Age 18.3±1.2 years | Strength training 2d/wk, 6wks, 12 sessions, In-season | Heavy loads (85-90% 1RM) and low volume (3-4 sets of 3-5 reps/set) of LB strength training (back squat, RDL and nordics) | N/A  Infrared timing gates (Speed Trap 2 Wireless Timing System; Draper, UT, USA). | 0-5m performance ↑ MD (s): 0.06; 95% CI [0.05, 0.07]; % Change 5.71%;  SMD: 1.63; 95% CI [1.06, 2.2] Weight 2.45%; Inference - Large  0-10m performance ↑ MD (s): 0.05; 95% CI [0.03, 0.07]; % Change 2.81%;  SMD: 1; 95% CI [0.56, 1.44] Weight 1.2%; Inference - Large  0-20m performance ↑ MD (s): 0.04; 95% CI [0.02, 0.06]; % Change 1.31%;  SMD: 0.61; 95% CI [0.25, 0.97] Weight 1.12%; Inference - Moderate |
| Suarez-Arrones et al. (2019) A (68) | M, n=12, Elite Soccer Players; Age 13±0.9 years | Strength training 1-2 d/wk, 15wks, 24 sessions, Phase not reported | 2-3 sets of 5-10 reps of nordic curl LB strength training | 8 hrs of soccer training + 1-2 competitive games/wk  Photoelectric cells (Witty, Microgate, Bolzano, Italy) | 0-5m performance = MD (s): 0.02; 95% CI [-0.01, 0.05]; % Change 2.02%;  SMD: 0.31; 95% CI [-0.09, 0.7] Weight 2.68%; Inference - Small  0-10m performance ↑ MD (s): 0.09; 95% CI [0.06, 0.12]; % Change 5.36%;  SMD: 1.2; 95% CI [0.72, 1.68] Weight 1.17%; Inference - Large  0-20m performance ↑ MD (s): 0.09; 95% CI [0.05, 0.13]; % Change 3.09%;  SMD: 0.79; 95% CI [0.41, 1.18] Weight 1.09%; Inference - Moderate |
| Suarez-Arrones et al. (2019) B (68) | M, n=17, Elite Soccer Players; Age 18.3±1.2 years | Strength training 1-2 d/wk, 17wks, 22 sessions, Phase not reported | 2-3 sets of 5-10 reps of nordic curl LB strength training | See Suarez-Arrones et al. (2019) A | 0-5m performance ↑ MD (s): 0.06; 95% CI [0.04, 0.08]; % Change 5.88%;  SMD: 1.07; 95% CI [0.6, 1.54] Weight 2.59%; Inference – Large  0-10m performance ↑ MD (s): 0.11; 95% CI [0.09, 0.13]; % Change 6.36%;  SMD: 1.69; 95% CI [1.14, 2.25] Weight 1.12%; Inference - Large  0-20m performance ↑ MD (s): 0.21; 95% CI [0.18, 0.24]; % Change 7.12%;  SMD: 2.19; 95% CI [1.58, 2.8] Weight 0.91%; Inference - Large |
| Thomas et al. (2009) A (69) | M, n=16, Elite Soccer Players; Age 18.8±0.8 years | Plyometrics training 2d/wk, 6wks, 12 sessions, In-season | High intensity plyometric (80-120 foot contacts/ session) training exercises (drop jumps 40cm) participants were instructed to minimise ground-contact time while maximising jump height | Soccer training 2-4 sessions/wk + 1-2 games/wk  Light gates (NewTest, Kiviharjuntie, Finland) | 0-5m performance = MD (s): -0.02; 95% CI [-0.07, 0.03]; % Change -1.9%;  SMD: -0.22; 95% CI [-0.81, 0.37] Weight 2.42%; Inference - Small  0-10m performance = MD (s): -0.02; 95% CI [-0.11, 0.07]; % Change -1.12%;  SMD: -0.12; 95% CI [-0.68, 0.44] Weight 1.12%; Inference – Trivial  0-20m performance = MD (s): 0.03; 95% CI [-0.08, 0.14]; % Change 0.98%;  SMD: 0.13; 95% CI [-0.38, 0.65] Weight 0.99%; Inference - Trivial |
| Thomas et al. (2009) B (69) | M, n=17, Elite Soccer Players; Age 18.8±0.8 years | Plyometrics training 2d/wk, 6wks, 12 sessions, In-season | High intensity plyometric (80-120 foot contacts/ session) training exercises (rebound jumps initiated from a counter-movement) participants were instructed to ‘‘damp’’ their landings each time and to gain maximum height through knee flexion | See Thomas et al. (2009) A | 0-5m performance = MD (s): -0.01; 95% CI [-0.1, 0.08]; % Change -0.93%;  SMD: -0.08; 95% CI [-0.77, 0.62] Weight 2.27%; Inference - Trivial  0-10m performance = MD (s): 0; 95% CI [-0.11, 0.11]; % Change 0%;  SMD: 0; 95% CI [-0.66, 0.66] Weight 1.04%; Inference - Trivial  0-20m performance = MD (s): 0.02; 95% CI [-0.14, 0.18]; % Change 0.63%;  SMD: 0.07; 95% CI [-0.53, 0.68] Weight 0.91%; Inference - Trivial |
| Torres-Torrelo et al. (2017) A (70) | M, n=7, Elite Soccer Players; Age 17.3±0.4 years | Strength training 2d/wk, 6wks, 12 sessions, In-season | Low load (~45–58% 1RM) LB velocity-based training low volume (1-1.2 m/s) 2-3 sets of 4–6 reps/set of full squat | 4 futsal training sessions + one official match/wk  Photocell timing gates (Polifemo Radio Light, Microgate, Bolzano, Italy) | 0-10m performance ↑ MD (s): 0.04; 95% CI [0.01, 0.07]; % Change 2.38%;  SMD: 0.53; 95% CI [0.08, 0.99] Weight 1.19%; Inference - Moderate  0-20m performance ↑ MD (s): 0.04; 95% CI [0, 0.08]; % Change 1.36%;  SMD: 0.43; 95% CI [0.02, 0.83] Weight 1.08%; Inference - Moderate |
| Torres-Torrelo et al. (2017) B (70) | M, n=5, Elite Soccer Players; Age 17.3±0.4 years | Strength and COD training 2d/wk, 6wks, 12 sessions, In-season | Low load (~45–58% 1RM) LB velocity-based training low volume (1-1.2 m/s) 2-3 sets of 4–6 reps/set of full squat + weighted COD task (5-10kg) 2-5 sets of 10s | See Torres-Torrelo et al. (2017) A | 0-10m performance = MD (s): 0.02; 95% CI [-0.02, 0.06]; % Change 1.17%;  SMD: 0.23; 95% CI [-0.2, 0.66] Weight 1.21%; Inference - Small  0-20m performance = MD (s): 0.03; 95% CI [-0.02, 0.08]; % Change 1.01%;  SMD: 0.26; 95% CI [-0.14, 0.66] Weight 1.09%; Inference - Small |
| Tous-Fajardo et al. (2016) A (71) | M, n=12, Sub-elite Futsal Players; Age 23.8±2.4 years | Strength training 1d/wk, 11wks, 11 sessions, In-season | Exercises performed in a circuit fashion, of moderate volume (2 sets of 6-8 reps/sets) resistance training exercises consisted of reverse wood chops, backward lunges, unilateral hamstrings “kicks” using an isoinertial portable conical pulley, lateral squats employing the YoYo squat and unilateral squats on a custom-made vibration platform + 2 sets of 6-8 reps of nordic curls | 3-4 soccer practices (~6 h), 1 session of strength/power exercises, and 1 competitive match (weekend). Skill training - warmup, technical actions, small-sided games, and tactical activities  Photoelectric cells (Musclelab, Ergotest Technology, Langesund, Norway) | 0-10m performance = MD (s): 0.01; 95% CI [-0.12, 0.14]; % Change 0.52%;  SMD: 0.03; 95% CI [-0.39, 0.46] Weight 1.21%; Inference - Trivial |
| Vera-Assaoka et al. (2019) A (72) | M, n=12, Sub-elite Futsal Players; Age 22.9±5.1 years | Plyometrics training 2d/wk, 7wks, 14 sessions, In-season | Mod-high intensity low volume (6 sets of 10 reps/set) plyometrics training (20-60 cm drop jumps) on a grass pitch surface | 2 times/ wk soccer training sessions of 90 min, consisting of 30 min of technical-tactical exercises, 30 min of small-sided games, and 30 min of simulated competitive games + an injury prevention session  Infrared reds photoelectric cells (Globus Italia, Codogne, Italy) | 0-20m performance = MD (s): 0.03; 95% CI [-0.07, 0.13]; % Change 0.63%;  SMD: 0.1; 95% CI [-0.24, 0.44] Weight 1.13%; Inference - Trivial |
| Vera-Assaoka et al. (2019) C (72) | M, n=12, Soccer Players; Age 17±0.5 years | Plyometrics training 2d/wk, 7wks, 14 sessions, In-season | Mod-high intensity low volume (6 sets of 10 reps/set) plyometrics training (20-60 cm drop jumps) on a grass pitch surface | See Vera-Assaoka et al. (2019) A | 0-20m performance = MD (s): 0.02; 95% CI [-0.12, 0.16]; % Change 0.51%;  SMD: 0.04; 95% CI [-0.25, 0.33] Weight 1.17%; Inference - Trivial |
| Winwood et al. (2015) B (73) | M, n=16, Soccer Players; Age 11.2±0.8 years | Strength training 2d/wk, 7wks, 14 sessions, Off-season | Moderate-heavy loads (70-85% 1RM) moderate volume 2-3 sets of 5-8 reps/ set) of clean and jerk, deadlift, military press, back squat and one arm row | 3 conditioning sessions, 3 technical sessions/wk  Electronic timing gates (Brower TC-System; Brower Timing Systems, Draper, UT, USA) | 0-5m performance = MD (s): 0; 95% CI [-0.02, 0.02]; % Change 0%;  SMD: 0; 95% CI [-0.4, 0.4] Weight 2.67%; Inference - Trivial  0-20m performance = MD (s): 0.01; 95% CI [-0.03, 0.05]; % Change 0.4%;  SMD: 0.09; 95% CI [-0.26, 0.45] Weight 1.12%; Inference - Trivial |
| Wong et al. (2010) A (74) | M, n=22, Soccer Players; Age 14.4±1 years | Strength and power training 2d/wk, 8wks, 16 sessions, Pre-season | Heavy loads (85%) and moderate volume (4 sets of 6 reps/set) of LB power + FB strength training (high pull, jump squat, half back squat, bench press, chin ups) | 6-8 soccer training sessions/wk, each lasting for 90 minutes + High-intensity interval training @120% MAS 15:15 work/rest 2/wk  Infrared photoelectronic cells (Speedtrap II Wireless Timing System, Brower Timing System, Australia) | 0-10m performance ↑ MD (s): 0.11; 95% CI [0.1, 0.12]; % Change 6.18%;  SMD: 5.5; 95% CI [4.18, 6.82] Weight 0.6%; Inference - Large |

M = male, F = female, UB = upper body, LB = lower body, FB = full body, 1RM = one-repetition maximum BW = bodyweight, BB = barbell, KB = kettlebell, DB = dumbbell, AEL = accentuated eccentric loading, SL = single leg, COD = change of direction, CMJ = countermovement jump, N/A = data not available, SMD = standardised mean difference, CI = confidence interval, MD, = mean difference, % Change = percentage change, d = day, wk(s) = week(s), hr(s) = hour(s), ↑ = significant increase in sprint performance (p = < 0.05) , = = no significant change in sprint performance (p = > 0.05), ↓ = significant decrease in sprint performance (p = < 0.05), the resistance and plyometric training intensity and volume descriptors are based descriptions from the study or previous guidelines (75-78).

# Declarations

**Ethics**

Approval was obtained from the ethics committee of Leeds Beckett University. The procedures used in this study comply with the ethical standards of the Declaration of Helsinki.

**Consent for publication**

Not applicable

**Availability of data and materials**

The datasets generated during and/or analysed during the current study are available from the corresponding author on reasonable request.

**Funding**

No sources of funding were used to assist in the preparation of this article.

**Conflicts of interest**

Ben Nicholson, Alex Dinsdale, Ben Jones and Kevin Till declare no potential conflicts of interest concerning the research, content, authorship, and/or publication of this review.

**Authors' contributions**

All the authors contributed to the manuscript, including the conception and design of the study, analysis and interpretation of the data, drafting and critically revising the manuscript, and approval for publication. All authors read and approved the final manuscript.

# References

1. Asadi A, Ramirez-Campillo R, Arazi H, Sáez de Villarreal E. The effects of maturation on jumping ability and sprint adaptations to plyometric training in youth soccer players. J Sports Sci. 2018:1-7.

2. Borges JH, Conceição MS, Vechin FC, Pascoal EHF, Silva RP, Borin JP. The effects of resisted sprint vs. plyometric training on sprint performance and repeated sprint ability during the final weeks of the youth soccer season. Sci Sports. 2016;31(4):e101-e5.

3. Bouguezzi R, Chaabene H, Negra Y, Ramirez-Campillo R, Jlalia Z, Mkaouer B, et al. Effects of different plyometric training frequency on measures of athletic performance in prepuberal male soccer players. J Strength Cond Res. 2018.

4. Brito J, Vasconcellos F, Oliveira J, Krustrup P, Rebelo A. Short-term performance effects of three different low-volume strength-training programmes in college male soccer players. J Hum Kinet. 2014;40(1):121-8.

5. Chelly MS, Ghenem MA, Abid K, Hermassi S, Tabka Z, Shephard RJ. Effects of in-season short-term plyometric training program on leg power, jump-and sprint performance of soccer players. J Strength Cond Res. 2010;24(10):2670-6.

6. Christou M, Smilios I, Sotiropoulos K, Volaklis K, Pilianidis T, Tokmakidis SP. Effects of resistance training on the physical capacities of adolescent soccer players. J Strength Cond Res. 2006;20(4):783-91.

7. Comfort P, Haigh A, Matthews MJ. Are changes in maximal squat strength during preseason training reflected in changes in sprint performance in rugby league players? J Strength Cond Res. 2012;26(3):772-6.

8. Coratella G, Beato M, Milanese C, Longo S, Limonta E, Rampichini S, et al. Specific adaptations in performance and muscle architecture after weighted jump-squat vs. body mass squat jump training in recreational soccer players. J Strength Cond Res. 2019;32(4):921-9.

9. Coutts AJ, Murphy AJ, Dascombe BJ. Effect of direct supervision of a strength coach on measures of muscular strength and power in young rugby league players. J Strength Cond Res. 2004;18(2):316-23.

10. de Hoyo M, Pozzo M, Sañudo B, Carrasco L, Gonzalo-Skok O, Domínguez-Cobo S, et al. Effects of a 10-week in-season eccentric-overload training program on muscle-injury prevention and performance in junior elite soccer players. Int J Sports Physiol Perform. 2015;10(1):46-52.

11. de Hoyo M, Gonzalo-Skok O, Sañudo B, Carrascal C, Plaza-Armas JR, Camacho-Candil F, et al. Comparative effects of in-season full-back squat, resisted sprint training, and plyometric training on explosive performance in U-19 elite soccer players. J Strength Cond Res. 2016;30(2):368-77.

12. Douglas J, Pearson S, Ross A, McGuigan M. Effects of accentuated eccentric loading on muscle properties, strength, power, and speed in resistance-trained rugby players. J Strength Cond Res. 2018;32(10):2750-61.

13. Gabbett TJ, Johns J, Riemann M. Performance changes following training in junior rugby league players. J Strength Cond Res. 2008;22(3):910-7.

14. García-Pinillos F, Martínez-Amat A, Hita-Contreras F, Martínez-López EJ, Latorre-Román PA. Effects of a contrast training program without external load on vertical jump, kicking speed, sprint, and agility of young soccer players. J Strength Cond Res. 2014;28(9):2452-60.

15. González-García J, Morencos E, Balsalobre-Fernández C, Cuéllar-Rayo Á, Romero-Moraleda B. Effects of 7-week hip thrust versus back squat resistance training on performance in adolescent female soccer players. Sports. 2019;7(4).

16. Griffiths B, Grant J, Langdown L, Gentil P, Fisher J, Steele J. The effect of in-season traditional and explosive Resistance training programs on strength, jump height, and speed in recreational soccer players. Res Q Exerc Sport. 2019;90(1):95-102.

17. Hammami M, Negra Y, Aouadi R, Shephard RJ, Chelly MS. Effects of an in-season plyometric training program on repeated change of direction and sprint performance in the junior soccer player. J Strength Cond Res. 2016;30(12):3312-20.

18. Hammami M, Gaamouri N, Shephard RJ, Chelly MS. Effects of contrast strength vs. plyometric training on lower limb explosive performance, ability to change direction and neuromuscular adaptation in soccer players. J Strength Cond Res. 2018.

19. Harries SK, Lubans DR, Buxton A, MacDougall THJ, Callister R. Effects of 12-weeks resistance training on sprint and jump performance in competitive adolescent rugby union players. J Strength Cond Res. 2017.

20. Helgerud J, Rodas G, Kemi O, Hoff J. Strength and endurance in elite football players. Int J Sports Med. 2011;32(9):677.

21. Impellizzeri FM, Rampinini E, Castagna C, Martino F, Fiorini S, Wisloff U. Effect of plyometric training on sand versus grass on muscle soreness and jumping and sprinting ability in soccer players. Br J Sports Med. 2007;42(1):42-6.

22. Ishøi L, Hölmich P, Aagaard P, Thorborg K, Bandholm T, Serner A. Effects of the Nordic Hamstring exercise on sprint capacity in male football players: a randomized controlled trial. J Sports Sci. 2017:1-10.

23. Kobal R, Loturco I, Barroso R, Gil S, Cuniyochi R, Ugrinowitsch C, et al. Effects of different combinations of strength, power, and plyometric training on the physical performance of elite young soccer players. J Strength Cond Res. 2017;31(6):1468-76.

24. Kobal R, Pereira LA, Zanetti V, Ramirez-Campillo R, Loturco I. Effects of Unloaded vs. Loaded Plyometrics on Speed and Power Performance of Elite Young Soccer Players. Front Physiol. 2017;8:742-.

25. Krommes K, Petersen J, Nielsen MB, Aagaard P, Hölmich P, Thorborg K. Sprint and jump performance in elite male soccer players following a 10-week Nordic Hamstring exercise protocol: a randomised pilot study. BMC Res Notes. 2017;10(1):669-.

26. Lago-Fuentes C, Rey E, Padrón-Cabo A, de Rellán-Guerra AS, Fragueiro-Rodríguez A, García-Núñez J. Effects of core strength training using stable and unstable surfaces on physical fitness and functional performance in professional female futsal players. J Hum Kinet. 2018;65(1):213-24.

27. Lockie RG, Murphy AJ, Scott BR, Janse de Jonge XA. Quantifying session ratings of perceived exertion for field-based speed training methods in team sport athletes. J Strength Cond Res. 2012;26(10):2721-8.

28. Lockie RG, Murphy AJ, Schultz AB, Knight TJ, de Jonge XAJ. The effects of different speed training protocols on sprint acceleration kinematics and muscle strength and power in field sport athletes. J Strength Cond Res. 2012;26(6):1539-50.

29. Lockie RG, Murphy AJ, Callaghan SJ, Jeffriess MD. Effects of sprint and plyometrics training on field sport acceleration technique. J Strength Cond Res. 2014;28(7):1790-801.

30. Los Arcos A, Yanci J, Mendiguchia J, Salinero JJ, Brughelli M, Castagna C. Short-term training effects of vertically and horizontally oriented exercises on neuromuscular performance in professional soccer players. Int J Sports Physiol Perform. 2014;9(3):480-8.

31. Loturco I, Ugrinowitsch C, Tricoli V, Pivetti B, Roschel H. Different loading schemes in power training during the preseason promote similar performance improvements in brazilian elite soccer players. J Strength Cond Res. 2013;27(7):1791-7.

32. Loturco I, Pereira LA, Kobal R, Zanetti V, Gil S, Kitamura K, et al. Half-squat or jump squat training under optimum power load conditions to counteract power and speed decrements in Brazilian elite soccer players during the preseason. J Sports Sci. 2015;33(12):1283-92.

33. Loturco I, Pereira LA, Kobal R, Zanetti V, Kitamura K, Abad CCC, et al. Transference effect of vertical and horizontal plyometrics on sprint performance of high-level U-20 soccer players. J Sports Sci. 2015;33(20):2182-91.

34. Loturco I, Nakamura FY, Kobal R, Gil S, Abad CCCAL, Cuniyochi R, et al. Training for power and speed: effects of increasing or decreasing jump squat velocity in elite young soccer players. J Strength Cond Res. 2015;29(10):2771-9.

35. Loturco I, Pereira LA, Kobal R, Maldonado T, Piazzi AF, Bottino A, et al. Improving Sprint Performance in Soccer: Effectiveness of Jump Squat and Olympic Push Press Exercises. Plos One. 2016;11(4):e0153958-e.

36. Loturco I, Nakamura F, Kobal R, Gil S, Pivetti B, Pereira L, et al. Traditional periodization versus optimum training load applied to soccer players: effects on neuromuscular abilities. Int J Sports Med. 2016;37(13):1051-9.

37. Loturco I, Kobal R, Kitamura K, Cal Abad CC, Faust B, Almeida L, et al. Mixed training methods: effects of combining resisted sprints or plyometrics with optimum power loads on sprint and agility performance in professional soccer players. Front Physiol. 2017;8:1034.

38. Loturco I, Pereira LA, Reis VP, Bishop C, Zanetti V, Alcaraz PE, et al. Power training in elite young soccer players: effects of using loads above or below the optimum power zone. J Sports Sci. 2019:1-7.

39. Manolopoulos E, Papadopoulos C, Salonikidis K, Katartzi E, Poluha S. Strength training effects on physical conditioning and instep kick kinematics in young amateur soccer players during preseason. Percept Mot Skills. 2004;99(2):701-10.

40. Manouras N, Papanikolaou Z, Karatrantou K, Kouvarakis P, Gerodimos V. The efficacy of vertical vs. horizontal plyometric training on speed, jumping performance and agility in soccer players. Int J Sports Sci Coach. 2016;11(5):702-9.

41. McMaster D, Gill N, McGuigan M, Cronin J. Effects of complex strength and ballistic training on maximum strength, sprint ability and force-velocity-power profiles of semi-professional rugby union players. J Aust Strength Cond. 2014;22(1):17-30.

42. Meylan C, Malatesta D. Effects of in-season plyometric training within soccer practice on explosive actions of young players. J Strength Cond Res. 2009;23(9):2605-13.

43. Nakamura D, Suzuki T, Yasumatsu M, Akimoto T. Moderate running and plyometric training during off-season did not show a significant difference on soccer-related high-intensity performances compared with no-training controls. J Strength Cond Res. 2012;26(12):3392-7.

44. Negra Y, Chaabene H, Hammami M, Hachana Y, Granacher URS. Effects of high-velocity resistance training on athletic performance in prepuberal male soccer athletes. J Strength Cond Res. 2016;30(12):3290-7.

45. Negra Y, Chaabene H, Sammoud S, Prieske O, Moran J, Ramirez-Campillo R, et al. The increased effectiveness of loaded versus unloaded plyometric-jump training in improving muscle power, speed, change-of-direction, and kicking-distance performance in prepubertal male soccer players. Int J Sports Physiol Perform. 2019;1:1-25.

46. Pienaar C, Coetzee B. Changes in selected physical, motor performance and anthropometric components of university-level rugby players after one microcycle of a combined rugby conditioning and plyometric training program. J Strength Cond Res. 2013;27(2):398-415.

47. Ramírez-Campillo R, Andrade DC, Izquierdo MJTJoS, Research C. Effects of plyometric training volume and training surface on explosive strength. J Sports Sci Med. 2013;27(10):2714-22.

48. Ramirez-Campillo R, Andrade DC, Álvarez C, Henríquez-Olguín C, Martínez C, Báez-SanMartín E, et al. The effects of interset rest on adaptation to 7 weeks of explosive training in young soccer players. J Sports Sci Med. 2014;13(2):287.

49. Ramírez-Campillo R, Meylan C, Álvarez C, Henríquez-Olguín C, Martínez C, Cañas-Jamett R, et al. Effects of in-season low-volume high-intensity plyometric training on explosive actions and endurance of young soccer players. J Strength Cond Res. 2014;28(5):1335-42.

50. Ramírez-Campillo R, Gallardo F, Henriquez-Olguín C, Meylan CM, Martínez C, Álvarez C, et al. Effect of vertical, horizontal, and combined plyometric training on explosive, balance, and endurance performance of young soccer players. J Strength Cond Res. 2015;29(7):1784-95.

51. Ramírez-Campillo R, Burgos CH, Henríquez-Olguín C, Andrade DC, Martínez C, Álvarez C, et al. Effect of unilateral, bilateral, and combined plyometric training on explosive and endurance performance of young soccer players. J Strength Cond Res. 2015;29(5):1317-28.

52. Ramírez-Campillo R, González-Jurado JA, Martínez C, Nakamura FY, Peñailillo L, Meylan CM, et al. Effects of plyometric training and creatine supplementation on maximal-intensity exercise and endurance in female soccer players. J Sci Med Sport. 2016;19(8):682-7.

53. Ramirez-Campillo R, Alvarez C, García-Pinillos F, Sanchez-Sanchez J, Yanci J, Castillo D, et al. Optimal reactive strength index: is it an accurate variable to optimize plyometric training effects on measures of physical fitness in young soccer players? J Strength Cond Res. 2018;32(4):885-93.

54. Ramirez-Campillo R, Alvarez C, Gentil P, Loturco I, Sanchez-Sanchez J, Izquierdo M, et al. Sequencing effects of plyometric training applied before or after regular soccer training on measures of physical fitness in young players. J Strength Cond Res. 2018.

55. Ramirez-Campillo R, Alvarez C, Gentil P, Moran J, García-Pinillos F, Alonso-Martínez AM, et al. Inter-individual variability in responses to 7 weeks of plyometric jump training in male youth soccer players. Front Physiol. 2018;9:1156-.

56. Ramirez-Campillo R, García-Pinillos F, García-Ramos A, Yanci J, Gentil P, Chaabene H, et al. Effects of different plyometric training frequencies on components of physical fitness in amateur female soccer players. Front Physiol. 2018;9.

57. Ramirez-Campillo R, Alvarez C, García-Pinillos F, Gentil P, Moran J, Pereira LA, et al. Effects of plyometric training on physical performance of young male soccer players: potential effects of different drop jump heights. Pediatr Exerc Sci. 2019;31(3):306-13.

58. Randell AD, Cronin JB, Keogh JWL, Gill ND, Pedersen MC. Effect of instantaneous performance feedback during 6 weeks of velocity-based resistance training on sport-specific performance tests. J Strength Cond Res. 2011;25(1):87-93.

59. Rimmer E, Sleivert G. Effects of a plyometrics intervention program on sprint performance. J Strength Cond Res. 2000;14(3):295-301.

60. Rodríguez-Rosell D, Torres-Torrelo J, Franco-Márquez F, González-Suárez JM, González-Badillo JJ. Effects of light-load maximal lifting velocity weight training vs. combined weight training and plyometrics on sprint, vertical jump and strength performance in adult soccer players. J Sci Med Sport. 2017;20(7):695-9.

61. Ronnestad BR, Kvamme NH, Sunde A, Raastad T. Short-term effects of strength and plyometric training on sprint and jump performance in professional soccer players. J Strength Cond Res. 2008;22(3):773-80.

62. Sanchez-Sanchez J, Perez S, Yaguee J, Royo J, Martin J. Implementation of a resistance training on young football players. Int J Med Sci Phys Act Sports. 2015;15(57):45-59.

63. Scott BR, Peiffer JJ, Goods PSR. The effects of supplementary low-load blood flow restriction training on morphological and performance-based adaptations in team sport athletes. J Strength Cond Res. 2017;31(8):2147-54.

64. Shalfawi SA, Ingebrigtsen J, Dillern T, Tønnessen E, Delp TK, Enoksen E. The effect of 40 m repeated sprint training on physical performance in young elite male soccer players. Serbian J Sports Sci. 2012;6(3).

65. Singh A, Kulkarni K, Shenoy S, Sandhu J. Effect of 6 weeks of preseason concurrent muscular strength and plyometric training in professional soccer players. J Postgrad Med Educ Res. 2014;48(1):27-32.

66. SÖhnlein Q, MÜller E, StÖggl TL. The effect of 16-week plyometric training on explosive actions in early to mid-puberty elite soccer players. J Strength Cond Res. 2014;28(8):2105-14.

67. Styles WJ, Matthews MJ, Comfort P. Effects of strength training on squat and sprint performance in soccer players. J Strength Cond Res. 2016;30(6):1534-9.

68. Suarez-Arrones L, Lara-Lopez P, Rodriguez-Sanchez P, Lazaro-Ramirez JL, Di Salvo V, Guitart M, et al. Dissociation between changes in sprinting performance and Nordic hamstring strength in professional male football players. Plos One. 2019;14(3):e0213375-e.

69. Thomas K, French D, Hayes PR. The effect of two plyometric training techniques on muscular power and agility in youth soccer players. J Strength Cond Res. 2009;23(1):332-5.

70. Torres-Torrelo J, Rodríguez-Rosell D, González-Badillo JJ. Light-load maximal lifting velocity full squat training program improves important physical and skill characteristics in futsal players. J Sports Sci. 2017;35(10):967-75.

71. Tous-Fajardo J, Gonzalo-Skok O, Arjol-Serrano JL, Tesch P. Enhancing change-of-direction speed in soccer players by functional inertial eccentric overload and vibration training. Int J Sports Physiol Perform. 2016;11(1):66-73.

72. Vera-Assaoka T, Ramirez-Campillo R, Alvarez C, Garcia-Pinillos F, Moran J, Gentil P, et al. Effects of maturation on physical fitness adaptations to plyometric drop jump training in male youth soccer players. J Strength Cond Res. 2019.

73. Winwood PW, Cronin JB, Posthumus LR, Finlayson SJ, Gill ND, Keogh JW. Strongman vs. traditional resistance training effects on muscular function and performance. J Strength Cond Res. 2015;29(2):429-39.

74. Wong P-l, Chaouachi A, Chamari K, Dellal A, Wisloff U. Effect of preseason concurrent muscular strength and high-intensity interval training in professional soccer players. J Strength Cond Res. 2010;24(3):653-60.

75. Chu DA. Jumping into plyometrics: Human Kinetics; 1998.

76. Haff GG, Triplett NT. Essentials of strength training and conditioning 4th edition: Hum Kinet; 2015.

77. Brearley S, Wild J, Agar-Newman D, Cizmic H. How to monitor net plyometric training stress: Guidelines for the coach. Prof Strength Cond. 2017;47:15-24.

78. Ebben WP. Practical guidelines for plyometric intensity. 2009.
