# Supplementary material for: The Training of Short Distance Sprint Performance in Football Code Athletes: A Systematic Review and Meta-Analysis
Source: Sports Med. 2020 Nov 27;51(6):1179–207. doi: 10.1007/s40279-020-01372-y (PMC8124057; doi:10.1007/s40279-020-01372-y)
Supplement: Supplementary file 2 — Supplementary file2 (DOCX 72 kb) [file 40279_2020_1372_MOESM2_ESM.docx]

**Electronic Supplementary Material Table S2**

Article title - The Training of Short Distance Sprint Performance in Football Code Athletes: A Systematic Review and Meta-Analysis

Journal name – Sports Medicine

Author names - Ben Nicholson, Alex Dinsdale, Ben Jones, and Kevin Till.

Affiliations - Leeds Beckett University, Carnegie Applied Rugby Research (CARR) centre, Carnegie School of Sport, Leeds, United Kingdom. Yorkshire Carnegie Rugby Union club, Leeds, United Kingdom. Leeds Rhinos Rugby League club, Leeds, United Kingdom. England Performance Unit, The Rugby Football League, Leeds, United Kingdom. School of Science and Technology, University of New England, Armidale, NSW, Australia. Division of Exercise Science and Sports Medicine, Department of Human Biology, Faculty of Health Sciences, the University of Cape Town and the Sports Science Institute of South Africa, Cape Town, South Africa.

corresponding author e-mail address – b.t.nicholson@leedsbeckett.ac.uk

**Table S2**

**Characteristics of the combined methods sprint training groups (primary and or secondary methods combined with tertiary methods) included in the review**

| **Study (year)** | **Subjects** | **Training type and organisation** | **Training methods and testing equipment** | **Other training** | **Mean difference, 95% CI, percentage change, Std. Mean Difference IV, Random, 95% CI, weight, Qualitative inference** |
| --- | --- | --- | --- | --- | --- |
| Alves et al. (2010) A (1) | M, n=9, Elite Soccer Players; Age 17.4±0.6 years | Strength, plyometrics and unresisted sprint training 1d/wk, 6wks, 6 sessions, Pre-season | High load (80-90%1RM) Low volume (1 set of 6 reps/set) of strength training (squat, calf raise and leg extension) and low-high intensity and low volume 1 set of 3-8 reps/set) plyometrics Exercise (bounding, drop jumps, seated box jump and CMJs + low volume (2 sets) of short sprints (20m) completed in tri-sets | Normal soccer training  Photocells (Smart Speed, Fusion Equipment, AUS) | 0-5m performance ↑ MD (s): 0.1; 95% CI [0.06, 0.14]; % Change 10.1%;  SMD: 1.46; 95% CI [0.72, 2.2] Weight 3.55%; Inference - Large  0-20m performance ↑ MD (s): 0.18; 95% CI [0.14, 0.22]; % Change 7.56%;  SMD: 1.88; 95% CI [1.12, 2.63] Weight 1.83%; Inference - Large |
| Alves et al. (2010) B (1) | M, n=8, Elite Soccer Players; Age 17.4±0.6 years | Strength, plyometrics and unresisted sprint training 2d/wk, 6wks, 12 sessions, Pre-season | High load (80-90%1RM) Low volume (1 set of 6 reps/set) of strength training (squat, calf raise and leg extension) and low-high intensity and low volume 1 set of 3-8 reps/set) plyometrics Exercise (bounding, drop jumps, seated box jump and CMJs + low volume (2 sets) of short sprints (20m) completed in tri-sets | See Alves et al. (2010) A | 0-5m performance ↑ MD (s): 0.07; 95% CI [0.05, 0.09]; % Change 6.6%;  SMD: 1.9; 95% CI [0.98, 2.81] Weight 3.09%; Inference - Large  0-20m performance ↑ MD (s): 0.08; 95% CI [0.03, 0.13]; % Change 3.21%;  SMD: 0.85; 95% CI [0.29, 1.41] Weight 2.16%; Inference - Large |
| Barr et al. (2015) A (2) | M, n=8, Elite Rugby Sevens Players; Age 22.4±2.7 years | Strength, power, resisted and unresisted sprint training 3-4d/wk, 3wks, 9 sessions, Pre-season | Speed training (~200 m of 10-25m sprints) and lightly weighted sled-resisted sprints up to 10m. Strength training 5-6 sets of LB strength-power training (2-6 reps/set) snatch or clean and jerk variations squats or jump squats and UB strength exercises (6-8 reps/set) UB pressing and pulling exercises + training for the abdominal muscles and some individualised injury prevention exercises. Simulated hypergravity using a weighted vest (12%BW) at all times when standing apart from training. | Rugby practice 2-4 times/wk focusing on technical passing, catching and kicking drills, as well as different conditioning games that varied in numbers per team (4–7), contact (touch rugby or full tackle/ rucks), and space (full-field or half-field)  Electronic timing system (Brower, Draper, UT, USA) | 0-10m performance = MD (s): -0.02; 95% CI [-0.06, 0.02]; % Change -1.12%;  SMD: -0.26; 95% CI [-0.79, 0.26] Weight 1.96%; Inference - Small  0-20m performance = MD (s): 0.02; 95% CI [-0.26, 0.3]; % Change 0.65%;  SMD: 0.03; 95% CI [-0.4, 0.46] Weight 1.98%; Inference - Trivial |
| Barr et al. (2015) B (2) | M, n=7, Elite Rugby Sevens Players; Age 22±2.1 years | Strength, plyometrics, resisted and unresisted sprint training 3-4d/wk, 3wks, 9 sessions, Pre-season | See Barr et al. (2015) A, matched training with no hypergravity weighted vest | See Barr et al. (2015) A | 0-10m performance = MD (s): -0.02; 95% CI [-0.06, 0.02]; % Change -1.12%;  SMD: -0.26; 95% CI [-0.83, 0.3] Weight 1.89%; Inference - Small |
| Beato et al. (2018) A (3) | M, n=11, Elite Soccer Players; Age 17±0.8 years | Plyometrics and unresisted sprint training 2d/wk, 6wks, 12 sessions, In-season | 3-4 sets of 3 short shuttle runs (sprints - distance not specified) with 4 changes of direction + moderate-high intensity moderate volume (5 sets of 4 reps/set) plyometrics (drop jumps from 60 cm followed by a jump over an obstacle 15 cm height + jumps over obstacles of 15 cm height). | 4 training sessions/wk as team practices and an official match every Saturday  Infrared timing gates (Microgate, Bolzano, Italy) | 0-10m performance ↑ MD (s): 0.05; 95% CI [0.01, 0.09]; % Change 2.82%;  SMD: 0.58; 95% CI [0.1, 1.06] Weight 2.04%; Inference - Moderate |
| Bianchi et al. (2019) A (4) | M, n=10, Elite Soccer Players; Age 17±0.8 years | Strength, plyometrics and unresisted sprint training 1d/wk, 8wks, 8 sessions, In-season | 4 Sets of 5-6 foot contacts/set of plyometric training using drop jumps (60cm) and horizontal jumps both followed by jumps over 2 obstacles (15cm) + strength and speed training (long and short) not specified | 4 training sessions/wk as team practices and an official match every Saturday + 3 sets of 3 short shuttle runs with 4 COD each, for an amount of 36 COD  Infrared timing gates (Microgate, Bolzano, Italy) | 0-10m performance ↑ MD (s): 0.05; 95% CI [0.01, 0.09]; % Change 2.79%;  SMD: 0.63; 95% CI [0.12, 1.13] Weight 1.99%; Inference - Moderate |
| Bianchi et al. (2019) B (4) | M, n=11, Elite Soccer Players; Age 17±0.8 years | Strength, plyometrics and unresisted sprint training 2d/wk, 8wks, 16 sessions, In-season | 4 Sets of 5-6 foot contacts/set of plyometric training using drop jumps (60cm) and horizontal jumps both followed by jumps over 2 obstacles (15cm) + strength and speed training (long and short) not specified | See Bianchi et al. (2019) A | 0-10m performance ↑ MD (s): 0.08; 95% CI [0.05, 0.11]; % Change 4.52%;  SMD: 1.05; 95% CI [0.5, 1.6] Weight 1.91%; Inference - Large |
| Brito et al. (2014) B (5) | M, n=12, Sub-elite Soccer Players; Age 20±0.6 years | Plyometrics and unresisted sprint training 2d/wk, 9wks, 18 sessions, In-season | Moderate-high intensity plyometric training low volume (1 set of 3-8 foot contacts/set) of high skipping, cyclically, with thighs parallel to the ground, keeping a frequency of movement as high as possible; 1 straight-line 5-m sprint, vertical jumps, trying to minimise ground contact time; ball headers jumping as high as possible; vertical jumps from a seated position; drop jumps (60 cm), executing a soccer heading, and trying to minimise ground contact time and maximise jump height. | Routine soccer training, based on technical and tactical drills, and small-sided games.  Photoelectric cells (Speed Trap II, Brower Timing Systems, USA) | 0-5m performance ↑ MD (s): 0.06; 95% CI [0.05, 0.07]; % Change 5.81%;  SMD: 3; 95% CI [1.96, 4.04] Weight 2.77%; Inference - Large  0-20m performance ↑ MD (s): 0.15; 95% CI [0.14, 0.16]; % Change 4.99%;  SMD: 5; 95% CI [3.56, 6.44] Weight 0.97%; Inference - Large |
| Brito et al. (2014) C (5) | M, n=12, Sub-elite Soccer Players; Age 19.9±0.5 years | Strength, plyometrics and unresisted sprint training 2d/wk, 9wks, 18 sessions, In-season | High intensity (80-90%) high-load weight training low volume (1 set of 6 reps/set) squat at 90°, calf extensions and leg extensions followed by plyometric exercises, set by set. Strength exercises increased by 5% from 1-RM every 3 weeks. Moderate-high intensity plyometric training low volume (1 set of 3-8 foot contacts/set) of high skipping, cyclically, with thighs parallel to the ground, keeping a frequency of movement as high as possible; 1 straight-line 5-m sprint, vertical jumps trying to minimise ground contact time; ball headers jumping as high as possible; vertical jumps from a seated position, trying to reach the highest point; drop jumps (60 cm) executing a soccer heading, and trying to minimise ground contact time and maximise jump height. | Routine soccer training, based on technical and tactical drills, and small-sided games.  Photoelectric cells (Speed Trap II, Brower Timing Systems, USA) | 0-5m performance ↑ MD (s): 0.1; 95% CI [0.09, 0.11]; % Change 10.71%;  SMD: 5; 95% CI [3.36, 6.64] Weight 1.68%; Inference - Large  0-20m performance ↑ MD (s): 0.2; 95% CI [0.19, 0.21]; % Change 6.49%;  SMD: 7.03; 95% CI [5.05, 9.02] Weight 0.61%; Inference - Large |
| Cavaco et al. (2014) A (6) | M, n=5, Soccer Players; Age 13.8±0.45 years | Strength training and unresisted sprint training 1d/wk, 6wks, 12 sessions, Phase not reported | High load (85% of 1-RM) moderate volume (6 sets of 6 reps/set) strength training (squat) performed a complex training. Sets 1-3 squats were performed as a complex with a 15 m sprint followed by a cross and sets 4-6 were performed with agility with the ball and shot at the goal. The load in the squat exercise was increased by 5% from the initial 1RM every 2 weeks. | Soccer training 5x/wk and 1 game/wk. Training sessions lasting ~90 mins included skill activities at various intensities, offensive and defensive tactics, and 30 mins of continuous play  Photocells (Speed Trap II - Browser Timing Systems) | 0-20m performance = MD (s): 0.13; 95% CI [-0.01, 0.27]; % Change 5.02%;  SMD: 0.55; 95% CI [-0.1, 1.21] Weight 2%; Inference - Moderate |
| Cavaco et al. (2014) B (6) | M, n=5, Soccer Players; Age 14.2±0.45 years | Strength training and unresisted sprint training 2d/wk, 6wks, 12 sessions, Phase not reported | High load (85% of 1-RM) moderate volume (6 sets of 6 reps/set) strength training (squat) performed a complex training. Sets 1-3 squats were performed as a complex with a 15 m sprint followed by a cross and sets 4-6 were performed with agility with the ball and shot at the goal. The load in the squat exercise was increased by 5% from the initial 1RM every 2 wks. | See Cavaco et al. (2014) B | 0-20m performance ↑ MD (s): 0.14; 95% CI [0.05, 0.23]; % Change 5.69%;  SMD: 0.96; 95% CI [0.23, 1.7] Weight 1.86%; Inference - Large |
| Corrêa et al. (2016) A (7) | M, n=10, Elite Soccer Players; Age 19.2±1.3 years | Strength, power and unresisted sprint training 3 strength training, 3 plyometrics training, 1 speed training d/wk, 9wks, 33 sessions, Pre-season | High loads (90% 1RM) and low volume (3 sets of 3 reps/set) UB and LB resistance training for 4 wks followed by moderate volume high-intensity plyometrics for 4 wks (4 sets of 8 foot contacts/set) and low volume sprint training (4 sets of 8 15m sprints/ set). | N/A  Photocells (CEFISE® Standard photocells, Brazil) | 0-20m performance ↓ MD (s): -0.07; 95% CI [-0.11, -0.03]; % Change -2.99%;  SMD: -0.82; 95% CI [-1.32, -0.32] Weight 2.27%; Inference - Large |
| Corrêa et al. (2016) B (7) | M, n=10, Elite Soccer Players; Age 19.2±1.3 years | Strength, power and unresisted sprint training 1 strength + 1 power d/wk, 19wks, 40 sessions, In-season | High loads (90% 1RM) and low volume (3 sets of 3 reps/set) UB and LB resistance training for 4 weeks followed by moderate volume high-intensity plyometrics for 4 wks (4 sets of 8 foot contacts/set). | See Corrêa et al. (2016) A | 0-20m performance ↓ MD (s): -0.04; 95% CI [-0.07, -0.01]; % Change -1.68%;  SMD: -0.53; 95% CI [-0.99, -0.07] Weight 1.04%; Inference - Moderate |
| Coutts et al. (2007) A (8) | M, n=7, Elite Rugby league Players; Age 25.7±2.6 years | Strength, power and unresisted sprint training 2-3d/wk, 6wks, 18 sessions, Pre-season | High load (78.5-86%1RM) strength and power training (3-4 sets of 5-8 reps/set). LB: prone hamstring flicks, box jumps (40 cm), hang clean, back squat, deadlift, BB step-ups (40 cm) and hamiglut raise. UB: internal/external shoulder, bench throw, push press, underhand weighted chin-ups, DB incline bench press, front military press, abdominal circuit + speed/agility training (not specified) | 5 – 7 sessions per week of field-based specific rugby league training, aerobic endurance development, skill and speed/agility training  Electronic timing gates (Swift, Lismore, Australia) | 0-10m performance = MD (s): 0.01; 95% CI [-0.04, 0.06]; % Change 0.53%;  SMD: 0.1; 95% CI [-0.45, 0.66] Weight 1.91%; Inference - Trivial |
| De Hoyo et al. (2016) C (9) | M, n=9, Elite Soccer Players; Age 18±1 years | Plyometrics and unresisted sprint training 2d/wk, 8wks, 16 sessions, In-season | Low-high intensity plyometrics 1-3 sets of 8-10 reps/set combined with short sprints (10-15m sprints). The exercises consisted of unilateral crossing jumps + 15-m sprint, lunges + 3m zigzag + 10-m sprint, unilateral alternative jumps + 15-m sprint, unilateral lateral jumps (40 cm hurdle) + 5-m zigzag + 10-m sprint, (e) speed ladder exercises, headers + 5-m sprint + deceleration + 2-m back running + 10-m sprint, double lateral jumps (20 cm hurdle) + zigzag + 10-m sprint, and unilateral lateral jumps + shooting without controlling the ball on an artificial grass surface. | 10 hrs of combined soccer (4-5 sessions) and conditioning (1 session) training, and 1 competitive match/wk.  Dual-beam electronic timing gate OptoJump System (Polifemo Radio Light, Microgate, Bolzano, Italy) | 0-10m performance = MD (s): 0; 95% CI [-0.04, 0.04]; % Change 0%;  SMD: 0; 95% CI [-0.49, 0.49] Weight 2.03%; Inference - Trivial  0-20m performance = MD (s): 0.01; 95% CI [-0.04, 0.06]; % Change 0.34%;  SMD: 0.09; 95% CI [-0.37, 0.54] Weight 2.34%; Inference - Trivial |
| De Villarreal et al. (2015) A (10) | M, n=13, Elite Soccer Players; Age 15.33±0.34 years | Strength, plyometrics and unresisted sprint training 2d/wk, 9wks, 18 sessions, In-season | Moderate load high volume half squats (2-4 sets of 6-10 reps/set) + low-moderate intensity high volume plyometrics (2-4 sets of 6-10 reps/set) (skipping 10-20m, vertical jumps, stride jumps, side long jumps and 2 step triple jumps) + 12-24 short sprints (10m) followed by technical dribbling and shooting | Soccer training 4 d/Wk (Monday, Wednesday, Thursday, and Friday)  Photoelectric cell (Muscle Lab. V7.18; Ergotest Technology) | 0-5m performance ↑ MD (s): 0.07; 95% CI [0.03, 0.11]; % Change 9.46%;  SMD: 0.7; 95% CI [0.22, 1.18] Weight 4.25%; Inference - Moderate  0-10m performance ↑ MD (s): 0.09; 95% CI [0.05, 0.13]; % Change 5.06%;  SMD: 0.9; 95% CI [0.42, 1.38] Weight 2.04%; Inference - Large |
| Enoksen et al. (2013) A (11) | M, n=9, Elite Soccer Players; Age 19±3.5 years | Strength, power and unresisted sprint training 2d/wk, 10wks, 20 sessions, Pre-season | Supervised hypertrophy training (6wk) moderate-high load (70-85%1RM) high volume (3 sets of 8-12 reps/set) followed by strength/ power training (4wk) and 2 sets of 40m sprints | 1.5 hrs soccer training during the intervention  Start mat and photocells | 0-10m performance = MD (s): 0.03; 95% CI [-0.01, 0.07]; % Change 1.94%;  SMD: 0.4; 95% CI [-0.1, 0.91] Weight 1.99%; Inference - Moderate |
| Enoksen et al. (2013) B (11) | M, n=8, Elite Soccer Players; Age 19±3.5 years | Strength, power and unresisted sprint training 2d/wk, 10wks, 20 sessions, Pre-season | Unsupervised hypertrophy training (6wk) moderate-high load (70-85%1RM) high volume (3 sets of 8-12 reps/set) followed by strength/ power training (4wk) and 2 sets of 40m sprints | See Enoksen et al. (2013) A | 0-10m performance = MD (s): -0.01; 95% CI [-0.04, 0.02]; % Change -0.65%;  SMD: -0.17; 95% CI [-0.69, 0.36] Weight 1.97%; Inference - Trivial |
| Faude et al. (2013) A (12) | M, n=8, Sub-elite Soccer Players; Age 23.1±2.7 years | Strength, power and unresisted sprint training 2d/wk, 7wks, 14 sessions, In-season | Day1: High load (90%1RM) unilateral half squats (4 sets of 4 reps/set) combined with SL hurdle jumps (4 sets of 5 reps/set). Day 2: exercises performed as a tri-set. 2-3 sets of 4-5 reps/set moderate load (50–60% 1RM) LB power exercises (half squats, calf raises, lateral half squats, step-ups) combined with plyometric exercises (drop jumps, high straight jumps, lateral jumps, bounding) and 2-3 sets of headers (3 reps/set) or short sprints 5m sprints or zigzag sprints (1 rep/set) | 4 soccer training sessions/wk and 1 competitive match at the weekend.  Single beam electronic timing gates (Racetime 2, Microgate, Bolzano, Italy) | 0-10m performance = MD (s): 0; 95% CI [-0.03, 0.03]; % Change 0%;  SMD: 0; 95% CI [-0.52, 0.52] Weight 1.97%; Inference - Trivial |
| Franco-Márquez et al. (2015) A (13) | M, n=20, Soccer Players; Age 14.7±0.5 years | Strength, plyometrics and unresisted sprint training 2d/wk, 6wks, 12 sessions, In-season | Velocity-based strength training (0.8-1.2 m/s full squat) 2-3 sets of 4-8 reps/set + moderate intensity plyometrics 3 sets of 5 foot contacts/set CMJs, 6 sets of 6-10 foot contacts/set step phase triple jumps + 1 set of 3-5 reps/set of 10s CODs and 1 set of 3-4 reps/set of short sprints (20m) | 4 sessions of soccer training/wk plus a 90-min match  Photocells (Polifemo Radio Light, Microgate, Bolzano, Italy) | 0-10m performance ↑ MD (s): 0.02; 95% CI [0, 0.04]; % Change 1.15%;  SMD: 0.33; 95% CI [0, 0.67] Weight 2.28%; Inference - Small  0-20m performance ↑ MD (s): 0.03; 95% CI [0, 0.06]; % Change 0.98%;  SMD: 0.31; 95% CI [0, 0.62] Weight 2.55%; Inference - Small |
| Gil et al. (2018) A (14) | M, n=9, Elite Soccer Players; Age 22.8±4.3 years | Power and unresisted sprint training 2d/wk, 6wks, 12 sessions, Pre-season | Moderate load (60%1RM) low volume 4-6 sets of 6 reps/set of squat jumps + 2-4 sets of 4 reps/set of unresisted short sprints (7m) and 2-4 sets of 4 reps/set of change of direction speed exercises consisting of short sprint shuttles and short sprints followed by a 45 degrees COD. | 3-4d/wk technical/tactical  Photocells (Smartspeed System, Fusion Sport, Australia | 0-5m performance ↑ MD (s): 0.08; 95% CI [0.03, 0.13]; % Change 8.16%;  SMD: 0.87; 95% CI [0.27, 1.47] Weight 3.92%; Inference - Large  0-10m performance ↑ MD (s): 0.09; 95% CI [0.04, 0.14]; % Change 5.36%;  SMD: 0.96; 95% CI [0.37, 1.56] Weight 1.84%; Inference - Large  0-20m performance ↑ MD (s): 0.08; 95% CI [0.02, 0.14]; % Change 2.72%;  SMD: 0.61; 95% CI [0.11, 1.1] Weight 2.27%; Inference - Moderate |
| Gil et al. (2018) B (14) | M, n=9, Elite Soccer Players; Age 22±2.2 years | Power and resisted sprint training 2d/wk, 6wks, 12 sessions, Pre-season | Moderate load (60%1RM) low volume 4-6 sets of 6 reps/set of squat jumps + 2-4 sets of 4 reps/set of resisted (load @ -10% Vmax) short sprints (7m) and 2-4 sets of 4 reps/set of change of direction speed exercises consisting of short sprint shuttles and short sprints followed by a 45 degrees COD. | See Gil et al. (2018) A | 0-5m performance ↑ MD (s): 0.06; 95% CI [0.01, 0.11]; % Change 6%;  SMD: 0.57; 95% CI [0.02, 1.13] Weight 4.05%; Inference - Moderate  0-10m performance ↑ MD (s): 0.1; 95% CI [0.05, 0.15]; % Change 5.92%;  SMD: 1.03; 95% CI [0.42, 1.63] Weight 1.82%; Inference - Large  0-20m performance ↑ MD (s): 0.11; 95% CI [0.07, 0.15]; % Change 3.69%;  SMD: 1.14; 95% CI [0.56, 1.72] Weight 2.12%; Inference - Large |
| González-Badillo et al. (2015) A (15) | M, n=17, Elite Soccer Players; Age 14.9±0.3 years | Strength, power, resisted and unresisted sprint training 2d/wk, 26wks, 52 sessions, In-season | Moderate load (50-65% 1RM) velocity-based strength training (80-105% of 1m/s load) 2-3 sets of 4-8 reps/set (full squat) + power and plyometric training 3-4 sets of 4-15 reps/set (loaded CMJ corresponding to a 20cm jump (40-70% load), box jump, hurdle jumps + 4-5 sets of 1 rep/set of resisted 20-25m sled towing (10-15kg) and 3-5 sets of 1 rep/set of short sprints (20m) | Soccer training 4 sessions/wk and one official game  Photocell timing gates (Polifemo Radio Light, Microgate, Bolzano, Italy) | 0-20m performance = MD (s): 0.03; 95% CI [0, 0.06]; % Change 1.01%;  SMD: 0.3; 95% CI [-0.04, 0.64] Weight 2.51%; Inference - Small |
| González-Badillo et al. (2015) B (15) | M, n=11, Elite Soccer Players; Age 19.2±1.2 years | Strength, power, resisted and unresisted sprint training 2d/wk, 26wks, 52 sessions, In-season | Moderate load (50-65% 1RM) velocity-based strength training (80-105% of 1m/s load) 2-3 sets of 4-8 reps/set (full squat) + power and plyometric training 3-4 sets of 4-15 reps/set (loaded CMJ corresponding to a 20cm jump (40-70% load), box jump, hurdle jumps + 4-5 sets of 1 rep/set of resisted 20-25m sled towing (10-15kg) and 3-5 sets of 1 rep/set of short sprints (20m) | See González-Badillo et al. (2015) A | 0-20m performance = MD (s): 0; 95% CI [-0.03, 0.03]; % Change 0%;  SMD: 0; 95% CI [-0.34, 0.34] Weight 2.51%; Inference - Trivial |
| González-Badillo et al. (2015) C (15) | M, n=16, Elite Soccer Players; Age 17.8±0.4 years | Strength, power, resisted and unresisted sprint training 2d/wk, 26wks, 52 sessions, In-season | Moderate load (50-65% 1RM) velocity-based strength training (80-105% of 1m/s load) 2-3 sets of 4-8 reps/set (full squat) + power and plyometric training 3-4 sets of 4-15 reps/set (loaded CMJ corresponding to a 20cm jump (40-70% load), box jump, hurdle jumps + 4-5 sets of 1 rep/set of resisted 20-25m sled towing (10-15kg) and 3-5 sets of 1 rep/set of short sprints (20m) | See González-Badillo et al. (2015) A | 0-20m performance = MD (s): -0.04; 95% CI [-0.08, 0]; % Change -1.35%;  SMD: -0.4; 95% CI [-0.83, 0.03] Weight 2.38%; Inference - Small |
| Gorostiaga et al. (2004) A (16) | M, n=8, Elite Soccer Players; Age 17.3±0.5 years | Strength, power, plyometrics and unresisted sprint training 2d/wk, 11wks, 19 sessions, In-season | Low load (40–60% 1RM) FB strength and power training (15-60s/set including Olympic lifting combined with plyometric training, bilateral plyometrics and sprints 1-5 sets of 2-8 reps/set. squat, power clean, maximal CMJ to box, unresisted sprint (short-medium distance sprints 15-40m) and vertical hurdle jumps.) | Soccer training 4 sessions/wk and one official game.  Photocell gates (Newtest, Oulu, Finland) | 0-5m performance = MD (s): 0.01; 95% CI [-0.01, 0.03]; % Change 1.05%;  SMD: 0.33; 95% CI [-0.23, 0.89] Weight 4.04%; Inference - Small  0-20m performance = MD (s): -0.01; 95% CI [-0.05, 0.03]; % Change -0.44%;  SMD: -0.12; 95% CI [-0.6, 0.37] Weight 2.29%; Inference - Trivial |
| Hammami et al. (2016) 2A (17) | M, n=12, Elite Soccer Players; Age 12.5±0.3 years | Strength and plyometrics training 2d/wk, 8wks, 16 sessions, In-season | 4wks plyometrics training followed by a 4wks balance training. Plyometric drills included CMJs, drop jumps +1 step, horizontal line jump, lateral hops, ankle jumps, SL cone hops, hurdle jumps, SL maximal rebound hops +5m acceleration 1-3 sets of 8-15 foot contacts/set. Balance training consisted of 1-3 sets of 30-45s/set kneeling swiss ball balancing, 8-15 reps/set of squats, SL straight leg bridges and lunges performed on an unstable surface e.g., foam surfaces, bosu balls or inflated disks. | None were involved in any after-school activities or any formalised strength and conditioning training programs.  Photocell gates (Brower Timing Systems, Salt Lake City, Utah, USA) | 0-10m performance ↑ MD (s): 0.2; 95% CI [0.12, 0.28]; % Change 10.53%;  SMD: 1.03; 95% CI [0.5, 1.55] Weight 1.96%; Inference - Large |
| Hammami et al. (2016) 2B (17) | M, n=12, Elite Soccer Players; Age 12.7±0.3 years | Strength and plyometrics training 2d/wk, 8wks, 16 sessions, In-season | 4wks balance training followed by a 4wks plyometrics training. Balance training consisted of 1-3 sets of 30-45s/set kneeling swiss ball balancing, 8-15 reps/set of squats, SSL straight leg bridges and lunges performed on an unstable surface e.g., foam surfaces, bosu balls or inflated disks. Plyometric drills included CMJs, drop jumps +1 step, horizontal line jump, lateral hops, ankle jumps, SL cone hops, hurdle jumps, SL maximal rebound hops +5m acceleration 1-3 sets of 8-15 foot contacts/set | See Hammami et al. (2016) 2A | 0-10m performance ↑ MD (s): 0.1; 95% CI [0.06, 0.14]; % Change 5%;  SMD: 1; 95% CI [0.48, 1.52] Weight 1.97%; Inference - Large |
| Hammami et al. (2018) A (18) | M, n=14, Elite Soccer Players; Age 16.1±0.5 years | Strength, plyometrics and unresisted sprint training 2d/wk, 8wks, 16 sessions, In-season | Moderate-high load (70-90% 1RM) moderate volume (3-5 sets of 3-8 reps/set) strength training (half squat) + low volume (3-5 sets of 1-3 foot contacts) low intensity plyometrics training (CMJ) + short sprints (15m) 3-5 sets of 1 rep/set (wk5-8 only) | Soccer training 4-5x/wk and 1 official game/wk  Paired photocell timers (Microgate, Bolzano, Italy) | 0-5m performance ↑ MD (s): 0.12; 95% CI [0.04, 0.2]; % Change 11.88%;  SMD: 0.65; 95% CI [0.2, 1.1] Weight 4.31%; Inference - Moderate |
| Harris et al. (2008) A (19) | M, n=7, Elite Rugby league Players; Age 21.8±4 years | Strength and unresisted sprint training 1-2d/wk, 7wks, 13 sessions, Pre-season | High loads hack squat machine squat jumps at 80% 1RM for 5 sets of 5 reps/set + other lower-body exercises at various loads, and UB training and sprint drills (not specified) | NA  Kinematic Measurement System (KMS, Optimal Kinetics, Ind) | 0-10m performance ↑ MD (s): 0.05; 95% CI [0.02, 0.08]; % Change 2.81%;  SMD: 1; 95% CI [0.32, 1.68] Weight 1.69%; Inference - Large |
| Harris et al. (2008) B (19) | M, n=8, Elite Rugby league Players; Age 21.8±4 years | Power and unresisted sprint training 1-2d/wk, 7wks, 13 sessions, Pre-season | Individually determined Pmax loads (20.0-43.5% 1RM) machine squat jumps for 6 sets of 10-12 reps/set + other lower-body exercises at various loads, and upper-body training and sprint drills (not specified) | See Harris et al. (2008) A | 0-10m performance = MD (s): 0.03; 95% CI [0, 0.06]; % Change 1.64%;  SMD: 0.45; 95% CI [-0.09, 1] Weight 1.93%; Inference - Moderate |
| Harrison and Bourke (2009) A (20) | M, n=8, Elite Rugby Players; Age 20.5±2.8 years | Strength, power and resisted sprint training 2d/wk, 6wks, 12 sessions, In-season | 1 set of 6 reps/set of resisted sled sprinting for short distances (20m) @13% BM + strength and power training (not specified) | Typical rugby training  Laser measurement device (LAVEG, Jenoptik, Sport) | 0-5m performance ↑ MD (s): 0.15; 95% CI [0.07, 0.23]; % Change 9.28%;  SMD: 1.02; 95% CI [0.35, 1.69] Weight 3.73%; Inference - Large  0-10m performance ↑ MD (s): 0.15; 95% CI [0.05, 0.25]; % Change 6.09%;  SMD: 0.8; 95% CI [0.21, 1.4] Weight 1.83%; Inference - Large |
| Koundourakis et al. (2014) A (21) | M, n=23, Elite Soccer Players; Age 25.5±1.1 years | Strength, plyometrics and unresisted sprint training 3d/wk, 21wks, 21 sessions, In-season | Moderate loads (70-80% 1RM) and high volume (4 sets of 10 repetitions/set) strength training combined with plyometric training and speed and agility training (not specified). Strength exercises consisted of lunge, squats, steps ups, pullovers, bicep curls, tricep extensions and bench press. Plyometric exercises consisted of skipping over cones, jumping on 1 or 2 legs, jumping over hurdles or obstacles (45 foot contacts/session). | 4 d/wk typical soccer training (simulated games, small-sided games, technical tactical drill and technical tactical training) + 1 game/wk  Infrared photoelectric cells (Powertimer, Newtest Ltd., Oulu, Finland), | 0-10m performance ↑ MD (s): 0.02; 95% CI [0, 0.04]; % Change 1.16%;  SMD: 0.33; 95% CI [0.02, 0.65] Weight 2.32%; Inference - Small  0-20m performance = MD (s): 0.01; 95% CI [-0.01, 0.03]; % Change 0.33%;  SMD: 0.18; 95% CI [-0.11, 0.46] Weight 2.58%; Inference - Trivial |
| Koundourakis et al. (2014) B (21) | M, n=22, Elite Soccer Players; Age 24.7±1 years | Strength, plyometrics and unresisted sprint training 2d/wk, 21wks, 21 sessions, In-season | High loads (90% 1RM) and moderate volume (4 sets of 5-6 repetitions/set) strength training combined with plyometric training and speed and agility training (not specified). Strength exercises consisted of Leg extension, hamstring curl, chest press, calf raise, pullovers, bicep curls and tricep extensions. Plyometric exercises consisted of skipping over cones, jumping on 1 or 2 legs, jumping over hurdles or obstacles (45 foot contacts/session). | See Koundourakis et al. (2014) A | 0-10m performance = MD (s): 0; 95% CI [-0.22, 0.22]; % Change 0%;  SMD: 0; 95% CI [-0.31, 0.31] Weight 2.32%; Inference - Trivial  0-20m performance = MD (s): 0; 95% CI [-0.02, 0.02]; % Change 0%;  SMD: 0; 95% CI [-0.29, 0.29] Weight 2.57%; Inference - Trivial |
| Koundourakis et al. (2014) C (21) | M, n=22, Elite Soccer Players; Age 23.8±0.9 years | Strength, plyometrics and unresisted sprint training 1 d/wk, 21wks, 21 sessions, In-season | High loads (90% 1RM) and moderate volume (4 sets of 5-6 repetitions/set) strength training combined with plyometric training and speed and agility training (not specified). Strength exercises consisted of Leg extension, hamstring curl, chest press, calf raise, pullovers, bicep curls and tricep extensions. Plyometric exercises consisted of skipping over cones, jumping on 1 or 2 legs, jumping over hurdles or obstacles (45 foot contacts/session). | 4 d/wk typical soccer training (simulated games, small sided games, technical tactical drill and Speed agility training and technical tactical training) + 1 game/wk  Infrared photoelectric cells (Powertimer, Newtest Ltd., Oulu, Finland), | 0-10m performance = MD (s): 0; 95% CI [-0.03, 0.03]; % Change 0%;  SMD: 0; 95% CI [-0.31, 0.31] Weight 2.32%; Inference - Trivial  0-20m performance = MD (s): 0.01; 95% CI [-0.01, 0.03]; % Change 0.33%;  SMD: 0.15; 95% CI [-0.14, 0.44] Weight 2.57%; Inference - Trivial |
| Lockie et al. (2012) 1A (22) | M, n=9, Sub-elite Team sport (i.e., rugby union, rugby league Australian rules football and soccer Players; Age 23.7±4.7 years | Resistance and unresisted sprint training 2d/wk, 6wks, 12 sessions, In-season | 1-3 sets of 3-5 reps/set of short distance sprints (5m, 10m, 15m and 20m sprints) and 2 gym-based training sessions/wk (not specified) | Normal physical activity, this generally consisted of 2 field based and 1 game/wk.  Velocimeter (Onspot, Wollongong, Australia) | 0-5m performance ↑ MD (m·s^-1^): 0.26; 95% CI [0.16, 0.36]; % Change 6.93%;  SMD: 1.33; 95% CI [0.62, 2.04] Weight 3.64%; Inference - Large  0-10m performance ↑ MD (m·s^-1^): 0.22; 95% CI [0.09, 0.35]; % Change 4.57%;  SMD: 0.85; 95% CI [0.28, 1.42] Weight 1.88%; Inference - Large |
| Lockie et al. (2012) 1B (22) | M, n=9, Sub-elite Team sport (i.e., rugby union, rugby league, Australian rules football and soccer Players; Age 23.7±4.7 years | Resistance and resisted sprint training 2d/wk, 6wks, 12 sessions, In-season | 1-3 sets of 3-5 reps/set of short distance resisted sled sprints (5m, 10m, 15m and 20m sprints) with a load corresponding to a 10% reduction in velocity and 2 gym-based training sessions/wk (not specified) | See Lockie et al. (2012) 1A | 0-5m performance ↑ MD (m·s^-1^): 0.27; 95% CI [0.12, 0.42]; % Change 7.09%;  SMD: 0.95; 95% CI [0.33, 1.57] Weight 3.88%; Inference - Large  0-10m performance ↑ MD (m·s^-1^): 0.27; 95% CI [0.12, 0.42]; % Change 5.64%;  SMD: 0.9; 95% CI [0.32, 1.48] Weight 1.87%; Inference - Large |
| Lockie et al. (2012) 2A (23) | M, n=9, Sub-elite Soccer, Rugby and Aussie Rules Players; Age 23.1±4.2 years | Resistance and unresisted sprint training 2d/wk, 6wks, 12 sessions, In-season | 1-3 sets of 3-5 reps/set of short distance sprints (5m, 10m, 15m and 20m sprints) and 2 gym-based training sessions/wk (not specified) | Normal physical activity, this generally consisted of 2 field based and 1 game/wk.  Velocimeter (Onspot, Wollongong, Australia) | 0-5m performance ↑ MD (m·s^-1^): 0.26; 95% CI [0.16, 0.36]; % Change 6.93%;  SMD: 1.33; 95% CI [0.62, 2.04] Weight 3.64%; Inference - Large  0-10m performance ↑ MD (m·s^-1^): 0.22; 95% CI [0.09, 0.35]; % Change 4.57%;  SMD: 0.85; 95% CI [0.28, 1.42] Weight 1.88%; Inference - Large |
| Lockie et al. (2012) 2D (23) | M, n=9, Sub-elite Soccer, Rugby and Aussie Rules Players; Age 23.1±4.2 years | Resistance and resisted sprint training 2d/wk, 6wks, 12 sessions, In-season | 1-3 sets of 3-5 reps/set of short distance resisted sled sprints (5m, 10m, 15m and 20m sprints) loaded @12.6% BM and 2 gym-based training sessions/wk (not specified) | See Lockie et al. (2012) 2A | 0-5m performance ↑ MD (m·s^-1^): 0.27; 95% CI [0.12, 0.42]; % Change 7.09%;  SMD: 0.95; 95% CI [0.33, 1.57] Weight 3.88%; Inference - Large  0-10m performance ↑ MD (m·s^-1^): 0.27; 95% CI [0.12, 0.42]; % Change 5.64%;  SMD: 0.9; 95% CI [0.32, 1.48] Weight 1.87%; Inference - Large |
| Lockie et al. (2014) A (24) | M, n=8, Sub-elite Soccer, Rugby and Aussie Rules Players; Age 21.81±2.59 years | Resistance and unresisted sprint training 2d/wk, 6wks, 12 sessions, In-season | 1-3 sets of 3-5 reps/set of short distance sprints (5m, 10m, 15m and 20m sprints) and 2 gym-based training sessions/wk (not specified) | Normal physical activity, this generally consisted of 2 field based and 1 game/wk.  Velocimeter (Onspot, Wollongong, Australia) | 0-5m performance ↑ MD (s): 0.08; 95% CI [0.04, 0.12]; % Change 6.45%;  SMD: 1.05; 95% CI [0.37, 1.73] Weight 3.71%; Inference - Large  0-10m performance ↑ MD (s): 0.11; 95% CI [0.06, 0.16]; % Change 5.61%;  SMD: 1.05; 95% CI [0.4, 1.69] Weight 1.75%; Inference - Large |
| Lopez-Segovia et al. (2010) A (25) | M, n=19, Elite Soccer Players; Age 18.43±0.6 years | Power, plyometrics and resisted sprint training 1-2d/wk, 15wks, 22 sessions, In-season | Velocity-based strength training (0.8-1.2 m/s full squat, +10-20% of full squat load for the half squat @ 1 m/s) 2-8 sets of 4 reps/set (full squat and half squat) + power and plyometric training 1-4 sets of 4-5 reps/set (loaded CMJ corresponding to a 20cm jump (40-70% load), CMJ, step phase triple jumps, displacements with loads + 4-5 sets of 1 rep/set of resisted 20-25m sled towing (10kg) and 3-6 sets of 1 rep/set of short sprints (20m) | 4 soccer field sessions/wk  Photoelectric cell barriers | 0-10m performance = MD (s): -0.03; 95% CI [-0.06, 0]; % Change -1.62%;  SMD: -0.32; 95% CI [-0.66, 0.03] Weight 2.27%; Inference - Small  0-20m performance ↓ MD (s): -0.07; 95% CI [-0.11, -0.03]; % Change -2.22%;  SMD: -0.57; 95% CI [-0.9, -0.23] Weight 2.51%; Inference - Moderate |
| Los Arcos et al. (2014) B (26) | M, n=8, Elite Soccer Players; Age 19.6±1.6 years | Power, plyometrics, resisted and unresisted sprint training 1-2d/wk, 8wks, 11 sessions, Pre-season and in-season | Light load (30-76% Peak power) low volume (2 sets of 5 reps/set) power training (Half squat and SL half squat, calf raise) + Low-high intensity and low volume (2-3 sets of 4-5 reps/set) horizontal orientated plyometric training (hip extension wall drills, triple jumps, loaded horizontal jumps @+ 5%BW, horizontal drop jumps) Resisted sled walks and sprints (5 sets of 1 rep/sets) over 10m @50-55%Bw and a load corresponding to 92.5% Vmax | Generic endurance training, as well as soccer specific training. Players played 6 friendly and 3 competitive matches during the 5 preseason and 3 in-season weeks, respectively.  Photocell beams (Newtest, Oulu, Finland) | 0-5m performance = MD (s): 0.01; 95% CI [-0.01, 0.03]; % Change 1.04%;  SMD: 0.33; 95% CI [-0.23, 0.89] Weight 4.04%; Inference - Small  0-20m performance = MD (s): 0.02; 95% CI [-0.02, 0.06]; % Change 0.87%;  SMD: 0.25; 95% CI [-0.24, 0.74] Weight 2.28%; Inference - Small |
| Loturco et al. (2017) A (27) | M, n=7, Elite Soccer Players; Age 21.7±2.4 years | Power and resisted sprint training 2-3d/wk, 5wks, 12 sessions, Pre-season | 6 sets of 4-8 reps/set of jump squats performed @ a load corresponding to the mass at which optimal power is produced (1-1.1* optimal power load) + 1 set of 8-6 reps/set of short-medium distance resisted sprints (20-30m @ 20-5%BM) | Pre-season soccer training 6/week + 2 pre-season friendly games  Photocells (Smart Speed, Fusion Equipment, AUS) | 0-5m performance ↑ MD (s): 0.11; 95% CI [0.09, 0.13]; % Change 11.68%;  SMD: 3.99; 95% CI [2.24, 5.73] Weight 1.54%; Inference - Large  0-10m performance ↑ MD (s): 0.1; 95% CI [0.08, 0.12]; % Change 5.99%;  SMD: 2.69; 95% CI [1.5, 3.89] Weight 0.98%; Inference - Large  0-20m performance ↑ MD (s): 0.07; 95% CI [0.05, 0.09]; % Change 2.47%;  SMD: 1.49; 95% CI [0.74, 2.24] Weight 1.85%; Inference - Large |
| Marques et al. (2019) A (28) | M, n=11, Futsal Players; Age 18.1±0.8 years | Strength, power, plyometrics and unresisted sprint training 2d/wk, 6wks, 12 sessions, In-season | 2-4 sets of of short distance sprints (10-20m), 2-4 sets 10s of a COD exercise, 2-3 sets of 5 foot contacts of depth jumps from one box to another (50-75cm), 2-3 sets of 4-6 reps/set of vertical jumps with handheld weights (4-8kg), and 2-3 sets of 6 reps/set horizontal leg-press (45-65% 1RM) | Futsal training 3d/wk + 1 match/wk  Photoelectric cells (Polifemo Radio Light, Microgate, Bolzano, Italy) | 0-10m performance ↑ MD (s): 0.04; 95% CI [0.01, 0.07]; % Change 2.2%;  SMD: 0.54; 95% CI [0.07, 1.01] Weight 2.05%; Inference - Moderate |
| McMorrow et al. (2019) A (29) | M, n=7, Elite Soccer Players; Age 24±3.6 years | Strength, power and unresisted sprint training 1-2d/wk, 6wks, 10 sessions, In-season | High load (85-95% 1RM) FB strength training 2-6 sets of 2-6 reps/set. The program consisted of front squat, split squat, stiff leg deadlift, Nordic hamstring curl, weighted step up, and supine bridge, landmine press, bench press, weighted pull up, and bent-over row; and stability exercises being Pallof press, abdominal rollout, side bridge, and prone bridge. All resistance training sessions concluded with a 2-4-min circuit of ballistic exercises consisting of squat jump, high pull, kettlebell swing, clap push up, pull up, and medicine ball slam. + 1 set of 5-9 reps of short distance sprints (20m) | Soccer training 3-4 times/wk (70-90 min)  Photoelectric timing gates (Smartspeed Lite; Fusion Sport, Coopers Plains, Australia) | 0-5m performance ↑ MD (s): 0.07; 95% CI [0.02, 0.11]; % Change 5.89%;  SMD: 0.89; 95% CI [0.2, 1.58] Weight 3.69%; Inference - Large  0-10m performance ↑ MD (s): 0.08; 95% CI [0.04, 0.12]; % Change 4.22%;  SMD: 1.16; 95% CI [0.44, 1.88] Weight 1.63%; Inference - Large  0-20m performance ↑ MD (s): 0.06; 95% CI [0.03, 0.1]; % Change 2.05%;  SMD: 0.9; 95% CI [0.29, 1.5] Weight 2.08%; Inference - Large |
| McMorrow et al. (2019) B (29) | M, n=6, Elite Soccer Players; Age 25.5±3.2 years | Strength, power and unresisted sprint training 1-2d/wk, 6wks, 10 sessions, In-season | High load (85-95% 1RM) FB strength training 2-6 sets of 2-6 reps/set. The program consisted of front squat, split squat, stiff leg deadlift, Nordic hamstring curl, weighted step up, and supine bridge, landmine press, bench press, weighted pull up, and bent-over row; and stability exercises being Pallof press, abdominal rollout, side bridge, and prone bridge. All resistance training sessions concluded with a 2-4-min circuit of ballistic exercises consisting of squat jump, high pull, kettlebell swing, clap push up, pull up, and medicine ball slam. + 1 set of 5-9 reps of short distance resisted sprints (20m) at 30% BM | See McMorrow et al. (2019) A | 0-5m performance ↑ MD (s): 0.06; 95% CI [0.03, 0.1]; % Change 5.83%;  SMD: 1.36; 95% CI [0.49, 2.24] Weight 3.18%; Inference - Large  0-10m performance ↑ MD (s): 0.1; 95% CI [0.06, 0.14]; % Change 5.26%;  SMD: 1.45; 95% CI [0.59, 2.31] Weight 1.4%; Inference - Large  0-20m performance ↑ MD (s): 0.1; 95% CI [0.05, 0.14]; % Change 3.11%;  SMD: 1.14; 95% CI [0.43, 1.86] Weight 1.9%; Inference - Large |
| Mendiguchia et al. (2015) A (30) | M, n=27, Sub-elite Soccer Players; Age 22.7±4.8 years | Strength, plyometrics, resisted sprint training, unresisted sprint training and sprint technique 2d/wk, 7wks, 14 sessions, In-season | Eccentric resistance training (e.g., Nordics, deadlift, hip thrust and lunge patterns) 2 sessions/wk + plyometrics (ECC box drops, bilateral and unilateral vertical and horizontal jumps + bounding) and acceleration training (wall drives, unresisted + resisted sprints (5-20m)) 1 session/wk. | Soccer training 3 sessions/wk and one official match at the weekend  Radar Stalker ATS SystemTM (Radar Sales, Minneapolis, Minnesota, USA) | 0-5m performance = MD (m·s^-1^): 0.06; 95% CI [-0.02, 0.14]; % Change 1.05%;  SMD: 0.22; 95% CI [-0.08, 0.53] Weight 4.65%; Inference - Small  0-20m performance = MD (m·s^-1^): -0.03; 95% CI [-0.12, 0.06]; % Change -0.38%;  SMD: -0.09; 95% CI [-0.35, 0.18] Weight 2.61%; Inference - Trivial |
| Michailidis et al. (2019) A (31) | M, n=17, Sub-elite Soccer Players; Age 11.8±0.8 years | 4 sets of 5-10 foot contacts/set of unilateral and bilateral vertical, lateral and horizontal jumps + speed and sprint workload (not specified) | 4 sets of 5-10 foot contacts/set of unilateral and bilateral vertical, lateral and horizontal jumps + speed and sprint workload (not specified) | Soccer training 3d/wk + 1 match/wk training session included technical skills, tactics and small-sided games  Infrared photoelectric gates (Microgate, Bolzano, Italy) | 0-10m performance ↑ MD (s): 0.1; 95% CI [0.06, 0.14]; % Change 4.53%;  SMD: 1; 95% CI [0.56, 1.44] Weight 2.12%; Inference - Large |
| Mujika et al. (2009) A (32) | M, n=10, Elite Soccer Players; Age 18.1±0.5 years | Strength, plyometrics, resisted and unresisted sprint training 1d/wk, 6wks, 6 sessions, In-season | Low load resistance training 3 sets of 2-4 reps/set (calf raises, half squats, weighted hip flexion/extension (15–50% body mass)) performed as contrast sets with plyometric training 3 sets of 3-6 foot contacts/set (ankleing, hurdle jumps, bounding and drop jumps [50cm]) and light load (13kg) and incline medium distance (25m) resisted sprints ( 2 sets of 5 reps/set) and short distance (10m) unresisted sprints (1 set of 6 reps/set) of with soccer-specific drills (small-sided games or technical skills). In addition, 1 session of stair climbing: 18 x (18 steps [22.5 cm] alternating single leg, double leg, single step, double step,  frontal step, lateral step. | N/A  Photocell gates (Timer S4, Alge-Timing, Lustenau, Austria) | 0-20m performance ↑ MD (m·s^-1^): 0.13; 95% CI [0.05, 0.21]; % Change 1.84%;  SMD: 0.71; 95% CI [0.23, 1.2] Weight 2.29%; Inference - Moderate |
| Nonnato et al. (2020) (33) | F, n=8, Elite Soccer Players; Age 23.4±4 years | Plyometrics, resistance training and unresisted sprint training 1d/wk, 12wks, 12 sessions, In-season | Low-high intensity plyometrics training 3-5 sets of 5-6 foot contacts/set (10-30cm hurdle jumps, 30-cm drop jumps, box jumps, lateral and horizontal jumps). Players also completed injury prevention exercises and UB strength training (not specified) and Speed training (long and short, volumes not specified) | Technical and tactical training (4d/wk) including moderate-intensity aerobic training or small sided games and 1 match/wk  Infrared timing gates (Microgate) | 0-10m performance ↑ MD (s): 0.18; 95% CI [0.15, 0.21]; % Change 9.52%;  SMD: 2.00; 95% CI [1.36, 2.64] Weight 1.75%; Inference - Large |
| Ozbar (2015) A (34) | F, n=10 Elite Soccer Players; Age 19.4±1.6 years | Plyometrics and unresisted sprint training 2d/wk, 10wks, 20 sessions, Phase not reported | Low-high intensity plyometrics training 3-5 sets of 5-8 foot contacts/set (horizontal, lateral, diagonal hurdle jumps, cone hops, lateral cone hops/jumps, standing long jumps, skipping, SL jumps, split squat jumps, vertical, lateral and horizontal jumps, jumps with 180 degree turns  combined with short distance sprints 1 set of 3-5 reps/set (5m) from various start positions/ actions | Technical and tactical training (4d/wk), moderate-intensity aerobic training or small sided games and preparation matches 1 times/wk  Infrared photoelectric cells (Newtest 2000 Sprint Timing System, NewtestOy, Oulu, Finland) | 0-10m performance = MD (s): 0.3; 95% CI [-0.09, 0.69]; % Change 15%;  SMD: 0.36; 95% CI [-0.12, 0.83] Weight 2.04%; Inference - Small  0-20m performance ↑ MD (s): 0.4; 95% CI [0.28, 0.52]; % Change 11.76%;  SMD: 1.41; 95% CI [0.8, 2.01] Weight 2.08%; Inference - Large |
| Ozbar et al. (2014) A (35) | F, n=9 Sub-elite Soccer Players; Age 18.3±2.6 years | Plyometrics and unresisted sprint training 1d/wk, 8wks, 8 sessions, In-season | Low-high intensity plyometrics training 3-5 sets of 5-12 foot contacts/set (horizontal, lateral, diagonal hurdle jumps, cone hops, lateral cone hops/jumps, standing long jumps, skipping, SL jumps, split squat jumps, vertical, lateral and horizontal jumps, jumps with 180 degree turns  combined with short distance sprints 1 set of 3-5 reps/set (5m) from various start positions/ actions | Soccer training 4d/wk + 1 game/wk.  Infrared photoelectric cells (Newtest 2000 Sprint Timing System, NewtestOy, Oulu, Finland) | 0-20m performance ↑ MD (s): 0.3; 95% CI [0.17, 0.43]; % Change 8.82%;  SMD: 1.06; 95% CI [0.49, 1.62] Weight 2.15%; Inference - Large |
| Rey et al. (2017) A (36) | M, n=10, Sub-elite Soccer Players; Age 23.6±2.7 years | Resisted sprint training 2d/wk, 6wks, 12 sessions, In-season | Sprinting wearing a weighted vest @18.9% ± 2.1% of BM (1-4 sets of 3-7 sprints/set) of short sprints (20m). | 4 sessions/wk soccer sessions containing low-intensity aerobic training, agility, and soccer-specific drills + 1 game/wk  Dual infrared reflex photoelectric cell system (DSD Laser System; Leo´ n, Spain) | 0-10m performance ↑ MD (s): 0.17; 95% CI [0.14, 0.2]; % Change 10.56%;  SMD: 2.57; 95% CI [1.61, 3.53] Weight 1.26%; Inference - Large |
| Rey et al. (2017) B (36) | M, n=9, Sub-elite Soccer Players; Age 23.7±2.1 years | Unresisted sprint training 2d/wk, 6wks, 12 sessions, In-season | Unresisted sprinting (1-4 sets of 3-7 sprints/set) of short sprints (20m). | See Rey et al. (2017) A | 0-10m performance ↑ MD (s): 0.2; 95% CI [0.17, 0.23]; % Change 12.58%;  SMD: 3.08; 95% CI [1.91, 4.25] Weight 1.01%; Inference - Large |
| Rodriguez-Rosell et al. (2016) 1A (37) | M, n=15, Elite Soccer Players; Age 12.7±0.5 years | Power and unresisted sprint training 2d/wk, 6wks, 12 sessions, Pre-season | Low loads (45-60% 1RM) and low volume (2 or 3 sets and 4-8 reps/set) resistance training combined with jumps (3*5) and short sprints (20m) | 4 field soccer training sessions/wk and 1 friendly match/wk. Each training session lasted on average 2 hrs and comprised various skill activities at different intensities, small-sided games, and finally 20 mins of continuous play or high-intensity interval training  Photocell timing gates (Polifemo Radio Light, Microgate, Bolzano, Italy) | 0-10m performance ↑ MD (s): 0.05; 95% CI [0.02, 0.08]; % Change 2.72%;  SMD: 0.76; 95% CI [0.33, 1.18] Weight 2.13%; Inference - Moderate  0-20m performance ↑ MD (s): 0.09; 95% CI [0.05, 0.13]; % Change 2.74%;  SMD: 0.82; 95% CI [0.41, 1.22] Weight 2.41%; Inference - Large |
| Rodriguez-Rosell et al. (2017) 1A (38) | M, n=15, Elite Soccer Players; Age 12.6±0.5 years | Power and unresisted sprint training 2d/wk, 6wks, 12 sessions, Pre-season | Low loads (45-60% 1RM) and low volume (2 or 3 sets and 4-8 reps/set) resistance training combined with jumps (3*5) and short sprints (20m) | 4 field soccer training sessions/wk and 1 friendly match/wk. Each training session lasted on average 2 hrs and comprised various skill activities at different intensities, small-sided games, and finally 20 mins of continuous play or high-intensity interval training  Photocell timing gates (Polifemo Radio Light, Microgate, Bolzano, Italy) | 0-10m performance ↑ MD (s): 0.06; 95% CI [0.03, 0.09]; % Change 3.26%;  SMD: 0.91; 95% CI [0.46, 1.36] Weight 2.09%; Inference - Large  0-20m performance ↑ MD (s): 0.09; 95% CI [0.05, 0.13]; % Change 2.74%;  SMD: 0.75; 95% CI [0.35, 1.15] Weight 2.42%; Inference - Moderate |
| Rodriguez-Rosell et al. (2017) 1C (38) | M, n=14, Elite Soccer Players; Age 14.6±0.5 years | Power and unresisted sprint training 2d/wk, 6wks, 12 sessions, Pre-season | Low loads (45-60% 1RM) and low volume (2 or 3 sets and 4-8 reps/set) resistance training combined with jumps (3*5) and short sprints (20m) | See Rodriguez-Rosell et al. (2017) 2A | 0-10m performance ↑ MD (s): 0.03; 95% CI [0.01, 0.05]; % Change 1.71%;  SMD: 0.5; 95% CI [0.08, 0.92] Weight 2.15%; Inference - Moderate  0-20m performance = MD (s): 0.04; 95% CI [0, 0.08]; % Change 1.29%;  SMD: 0.36; 95% CI [-0.01, 0.74] Weight 2.46%; Inference - Small |
| Rodriguez-Rosell et al. (2017) 1E (38) | M, n=14, Elite Soccer Players; Age 16.4±0.5 years | Power and unresisted sprint training 2d/wk, 6wks, 12 sessions, Pre-season | Low loads (45-60% 1RM) and low volume (2 or 3 sets and 4-8 reps/set) resistance training combined with jumps (3*5) and short sprints (20m) | See Rodriguez-Rosell et al. (2017) 2A | 0-10m performance ↑ MD (s): 0.04; 95% CI [0.02, 0.06]; % Change 2.38%;  SMD: 0.67; 95% CI [0.23, 1.1] Weight 2.12%; Inference - Moderate  0-20m performance ↑ MD (s): 0.04; 95% CI [0, 0.08]; % Change 1.36%;  SMD: 0.39; 95% CI [0.01, 0.76] Weight 2.46%; Inference - Small |
| Rodriguez-Rosell et al. (2017) 2B (39) | M, n=10, Elite Soccer Players; Age 24.5±3.4 years | Power, plyometrics and unresisted sprint training 2d/wk, 6wks, 12 sessions, In-season | Light loads (45-60% 1RM) and low volume (2-3 sets and 4-8 reps/set) power training (full squat) combined with (3 sets of 5) and short sprints (20m) | 4 field soccer training sessions/wk and 1 friendly match/wk. Each training session lasted on average 2 hrs and comprised various skill activities at different intensities, small-sided games, and finally 20 mins of continuous play or high-intensity interval training  Infrared timing system (Optojump, Microgate, Bolzano, Italy) | 0-10m performance ↑ MD (s): 0.07; 95% CI [0.03, 0.11]; % Change 4.09%;  SMD: 0.81; 95% CI [0.28, 1.35] Weight 1.94%; Inference - Large  0-20m performance ↑ MD (s): 0.09; 95% CI [0.03, 0.15]; % Change 3.03%;  SMD: 0.66; 95% CI [0.19, 1.14] Weight 2.3%; Inference - Moderate |
| Ross et al. (2015) A (40) | M, n=5, Elite Rugby Sevens Players; Age 20±1 years | Strength, power and resisted sprint training 2d/wk, 4wks, 8 sessions, In-season | High-force group session 1: Short resisted sprints (2-3 sets of 4-5 reps/set) 10 m sled sprint (30 kg) and heavy sled push (10 m)(3 sets of 3-4 reps/set) + ballistic med ball throws (3 sets of 3-4 reps/set) reverse overhead medicine ball throw @12kg). Session 2: Moderate-heavy load low volume strength speed/strength exercises (4 sets of 3/4 reps/set Countermovement squat jump @55-65%1RM, band resisted horizontal jump, ½ Squat@75-90%1RM) | In addition to their respective training program, all players also performed two UB strength sessions (65-85% 1RM, 3-5 exercises, 4 sets of 4-6 reps/set), two 60-min rugby skills sessions, and one 45-minute, field-based aerobic conditioning session.  Timing lights (Smart Speed, Swift Performance Equipment, AUS) | 0-10m performance = MD (s): -0.02; 95% CI [-0.05, 0.01]; % Change -1.13%;  SMD: -0.4; 95% CI [-1.08, 0.28] Weight 1.69%; Inference - Small |
| Ross et al. (2015) B [(40) | M, n=5, Elite Rugby Sevens Players; Age 21±2 years | Power, plyometrics and resisted sprint training 2d/wk, 4wks, 8 sessions, In-season | High-velocity group session 1: Medium length 10m flying sprint (20 m build up) (2-3 sets of 4-5 reps/set) 3-hurdle bound (60-75 cm) (3 sets of 3-4 reps/set) + balistic medball throws (3 sets of 3-4 reps/set) reverse overhead medicine ball throw @3kg). Session 2: Moderate-light to moderate load low volume strength/speed exercises (4 sets of 3/4 reps/set Countermovement squat jump @20-30%1RM, horizontal drop jump, speed squat @50-60%1RM) | See Ross et al. (2015) A | 0-10m performance = MD (s): 0.04; 95% CI [-0.51, 0.59]; % Change 2.37%;  SMD: 0.05; 95% CI [-0.61, 0.7] Weight 1.73%; Inference - Trivial |
| Seitz (2015) A (41) | M, n=10, Elite Rugby league Players; Age 18.4±0.9 years | Weightlifting and unresisted sprint training 2d/wk, 8wks, 16 sessions, Pre-season | Unresisted maximal sprinting (2 sets of 2-8 sprints/set) of short-medium sprints (10-40m). Players were required to catch the ball from a pass or a kick before sprinting and carry the ball under one arm while sprinting + 3 hours/wk of weightlifting (not specified). | A typical training week included 2 hrs of skills and tactical training, 3 hrs of conditioning, 3 hrs of weightlifting and 1 hr focused on injury prevention and recovery (11 sessions in total)  Electronic timing gates (Microgate Photocell, Bolzano, Italy | 0-20m performance ↑ MD (s): 0.09; 95% CI [0.04, 0.14]; % Change 3%;  SMD: 0.85; 95% CI [0.35, 1.35] Weight 2.26%; Inference - Large |
| Seitz (2015) B (41) | M, n=10, Elite Rugby League Players; Age 18.4±0.9 years | Weightlifting and unresisted sprint training 2d/wk, 8wks, 16 sessions, Pre-season | Unresisted maximal sprinting (2 sets of 2-8 sprints/set) of short-medium sprints (10-40m) + 3 hrs/wk of weightlifting (not specified). | See Seitz (2015) A | 0-20m performance ↑ MD (s): 0.08; 95% CI [0.03, 0.13]; % Change 2.62%;  SMD: 0.71; 95% CI [0.23, 1.19] Weight 2.29%; Inference - Moderate |
| Shalfawi et al. (2012) A (42) | M, n=8, Elite Soccer Players; Age 16.3±0.5 years | Strength and unresisted sprint training 2d/wk, 8wks, 16 sessions, Phase not reported | Unresisted maximal sprinting (4 sets of 5 sprints/set) of medium sprints (40m). Sprint training additional to typical soccer training. + Resistance training Nordic curls, balance training (ankle strength on balance board), sit-ups, the plank, push-ups and the alternating back and arm raise 2 x/wk during soccer training (volume/ intensity not provided). | Soccer training 4x/wk  Newtest Powertimer 300s infrared photocells | 0-20m performance ↑ MD (s): 0.19; 95% CI [0.12, 0.26]; % Change 6.13%;  SMD: 1.39; 95% CI [0.72, 2.06] Weight 1.97%; Inference - Large |
| Spinks et al. (2007) A (43) | M, n=10, Elite Soccer, Rugby union, Aussie Rules Players; Age 21.8±4.2 years | Strength training and resisted sprint training 2d/wk, 8wks, 16 sessions, In-season | Resisted maximal sprinting (5-8 sets of 3-6 sprints/set) of short sprints (5-20m). Resistance load @10% dec in 15 Vmax + 2 strength and fitness training sessions/wk (not specified). | Normal training, which consisted of at least 2 soccer training and 2 strength and fitness training sessions/wk and at least 1 game/wk  Velocimeter (Onspot, Wollongong, Australia) | 0-5m performance ↑ MD (m·s^-1^): 0.33; 95% CI [0.19, 0.47]; % Change 9.12%;  SMD: 1.17; 95% CI [0.54, 1.81] Weight 3.84%; Inference - Large  0-10m performance ↑ MD (m·s^-1^): 0.39; 95% CI [0.26, 0.52]; % Change 6.08%;  SMD: 1.44; 95% CI [0.78, 2.1] Weight 1.72%; Inference - Large  0-20m performance ↑ MD (m·s^-1^): 0.53; 95% CI [0.37, 0.69]; % Change 7.37%;  SMD: 1.46; 95% CI [0.84, 2.07] Weight 2.06%; Inference - Large |
| Spinks et al. (2007) B (43) | M, n=10, Elite Soccer, Rugby union, Aussie Rules Players; Age 21.8±4.2 years | Strength training and unresisted sprint training 2d/wk, 8wks, 16 sessions, In-season | Maximal sprinting (5-8 sets of 3-6 sprints/set) of short sprints (5-20m). Sprint training additional to typical soccer training + 2 strength and fitness training sessions/wk (not specified). | See Spinks et al. (2007) A | 0-5m performance ↑ MD (m·s^-1^): 0.28; 95% CI [0.13, 0.43]; % Change 7.98%;  SMD: 0.91; 95% CI [0.33, 1.49] Weight 3.98%; Inference - Large  0-10m performance ↑ MD (m·s^-1^): 0.28; 95% CI [0.13, 0.43]; % Change 4.38%;  SMD: 0.88; 95% CI [0.33, 1.42] Weight 1.92%; Inference - Large  0-20m performance ↑ MD (m·s^-1^): 0.39; 95% CI [0.26, 0.52]; % Change 5.58%;  SMD: 1.25; 95% CI [0.68, 1.83] Weight 2.13%; Inference - Large |
| Tønnessen et al. (2011) A (44) | M, n=10, Elite Soccer Players; Age 16.4±0.9 years | Strength and unresisted sprint training 1d/wk, 10wks, 10 sessions, Pre-season | Unresisted sprint training 2-4 sets of 4-5 reps of moderate distance sprints (40m) @ 95-100% max effort + Nordics curls 2-4 sets of 10 reps/set | Soccer training 3-4 times/wk. Consisting of tactical attack and defence drills and small and large sided games.  Photo cells | 0-20m performance = MD (s): 0; 95% CI [-0.04, 0.04]; % Change 0%;  SMD: 0; 95% CI [-0.43, 0.43] Weight 2.37%; Inference - Trivial |
| Tous-Fajardo et al. (2016) B (45) | M, n=12, Soccer Players; Age 17±0.5 years | Strength, plyometrics and unresisted sprint training 1d/wk, 11wks, 11 sessions, In-season | Exercises performed in a contrast tri-set format (strength exercises, plyometrics, sprinting/header simulations). Low-moderate load (50-100% body mass) moderate volume (2 sets of 6-10 reps/set) resistance training exercises (lunges, half squats, calf raises). Low-moderate intensity moderate volume plyometrics training (skipping, CMJs, reactive calf jumps, jumps to header the ball) 2 sets of 6-10 reps/set and short-distance sprints (10m) 2 sets of 2 reps/set. | 3-4 soccer practices (~6 h), 1x session of strength/power exercises, and 1x competitive match (weekend). Skill training - warmup, technical actions, small-sided games, and tactical activities  Photoelectric cells (Musclelab, Ergotest Technology, Langesund, Norway) | 0-10m performance ↓ MD (s): -0.11; 95% CI [-0.15, -0.07]; % Change -5.82%;  SMD: -1.15; 95% CI [-1.69, -0.6] Weight 1.93%; Inference - Large |
| Weakley et al. (2019) A (46) | M, n=16, Elite Rugby union Players; Age 21±1 years | Strength, power and unresisted sprint training 3d/wk, 4wks, 12 sessions, Pre-season | High load FB strength training (85-93% 1RM) 3 sets of 2-5 reps/set (session 1 - back squat, bench press; session 2 - hex bar deadlift and bench press). + bodyweight strength training session 1 - nordic drops 3 sets of 4 reps/set and side plank 3 sets of 1 min; session 2 single leg glute bridges 3 sets of 10 reps/set and front plank 3 sets of 1min + low load (20%) or bodyweight power training 3 sets of 2-3 reps of trap bar squat jump and standing long jump + 2 sets of 2 reps of short-distance sprints (15m). Augmented feedback was provided on training (sprints - time for distance, broad jump - distance, strength and power exercises - linear position transducers) | 3 rugby union training sessions/wk  Timing gates (Brower Timing Systems; IR EMIT, USA) | 0-10m performance ↑ MD (s): 0.08; 95% CI [0.03, 0.13]; % Change 4.55%;  SMD: 0.64; 95% CI [0.23, 1.04] Weight 2.18%; Inference - Moderate  0-20m performance ↑ MD (s): 0.11; 95% CI [0.05, 0.17]; % Change 3.62%;  SMD: 0.64; 95% CI [0.27, 1.01] Weight 2.46%; Inference - Moderate |
| Weakley et al. (2019) B (46) | M, n=12, Elite Rugby union Players; Age 21±2 years | Strength, power and unresisted sprint training 3d/wk, 4wks, 12 sessions, Pre-season | High load FB strength training (85-93% 1RM) 3 sets of 2-5 reps/set (session 1 - back squat, bench press; session 2 - hex bar deadlift and bench press). + bodyweight strength training session 1 - nordic drops 3 sets of 4 reps/set and side plank 3 sets of 1 min; session 2 single leg glute bridges 3 sets of 10 reps/set and front plank 3 sets of 1min + low load (20%) or bodyweight power training 3 sets of 2-3 reps of trap bar squat jump and standing long jump + 2 sets of 2 reps of short-distance sprints (15m). No feedback was provided. | See Weakley et al. (2019) B | 0-10m performance = MD (s): 0.04; 95% CI [0, 0.08]; % Change 2.26%;  SMD: 0.42; 95% CI [-0.02, 0.86] Weight 2.11%; Inference - Moderate  0-20m performance ↑ MD (s): 0.05; 95% CI [0.01, 0.09]; % Change 1.63%;  SMD: 0.5; 95% CI [0.08, 0.92] Weight 2.4%; Inference - Moderate |
| West et al. (2013) A (47) | M, n=10, Elite Rugby union Players; Age 26.8±3 years | Strength, resisted and unresisted sprint training 2d/wk, 6wks, 12 sessions, Pre-season | Maximal sprinting (2 sets of 6 sprints/set) + sled towing sprints (2 sets of 6 sprints/set) of short sprints (20m). Resistance load (12.6%Bw) on a rubber crumb surface + 3 resistance training sessions 1 UB, 1 LB, and 1 FB (not specified) | 3 conditioning sessions, 3 technical sessions/wk  Electronic timing gates (Brower TC-System; Brower Timing Systems, Draper, UT, USA) | 0-10m performance = MD (s): 0.04; 95% CI [-0.01, 0.09]; % Change 2.35%;  SMD: 0.4; 95% CI [-0.08, 0.88] Weight 2.04%; Inference - Moderate |
| West et al. (2013) B (47) | M, n=10, Elite Rugby union Players; Age 25.1±3.2 years | Strength and unresisted sprint training 2d/wk, 6wks, 12 sessions, Pre-season | Maximal sprinting (4 sets of 6 sprints/set) of short sprints (20m) on a rubber crumb surface + 3 resistance training sessions 1 UB, 1 LB, and 1 FB (not specified). | See West et al. (2013) A | 0-10m performance = MD (s): 0.02; 95% CI [-0.01, 0.05]; % Change 1.16%;  SMD: 0.3; 95% CI [-0.17, 0.78] Weight 2.05%; Inference - Small |
| Winwood et al. (2015) A (48) | M, n=15, Mixed (elite and sub-elite) Rugby Players; Age 23.4±5.6 years | Strength training 2d/wk, 7wks, 14 sessions, Off-season | Moderate-heavy loads (70-85% 1RM) moderate volume 2-3 sets of 5-8 reps/ set) of log lift, 28m farmers carry, axle press heavy sled pull 25m and arm over arm prowler pull | 2 sessions of prehabilition exercises/wk and 2 cardiovascular training sessions focused on improving aerobic capacity  Wireless dual beam timing lights (Swift Performance Equipment). | 0-5m performance = MD (s): 0.02; 95% CI [0, 0.04]; % Change 1.83%;  SMD: 0.36; 95% CI [-0.05, 0.77] Weight 4.41%; Inference - Small  0-20m performance = MD (s): 0.01; 95% CI [-0.03, 0.05]; % Change 0.4%;  SMD: 0.1; 95% CI [-0.25, 0.45] Weight 2.49%; Inference - Trivial |
| Yanci et al. (2016) A (49) | M, n=8, Elite Soccer Players; Age 22.5±5.04 years | Plyometrics and unresisted sprint training 2d/wk, 6wks, 12 sessions, In-season | Low-high intensity and low-moderate volume horizontal orientated plyometrics training (180 foot contacts/session). Day 1 CMJ jumps (bilateral and unilateral) Day 2 drop jump exercises (bilateral and unilateral). WK4-6 jumps @+10%BW. Additional sprint training in the week (not specified). | Endurance and soccer-specific training 3–4 d/wk and 1 official match/wk during the competitive period  Photocell gates (Microgate™ Polifemo, Bolzano, Italy) | 0-5m performance = MD (s): -0.01; 95% CI [-0.04, 0.02]; % Change -0.98%;  SMD: -0.22; 95% CI [-0.77, 0.34] Weight 4.06%; Inference - Small  0-20m performance = MD (s): 0; 95% CI [-0.05, 0.05]; % Change 0%;  SMD: 0; 95% CI [-0.48, 0.48] Weight 2.29%; Inference - Trivial |
| Yanci et al. (2016) B (49) | M, n=8, Elite Soccer Players; Age 24.63±2.72 years | Plyometrics and unresisted sprint training 2d/wk, 6wks, 12 sessions, In-season | See Yanci et al. (2016) A, high volume group (360 foot contacts/session) | See Yanci et al. (2016) A | 0-5m performance = MD (s): -0.01; 95% CI [-0.04, 0.02]; % Change -0.98%;  SMD: -0.18; 95% CI [-0.73, 0.37] Weight 4.06%; Inference - Trivial  0-20m performance = MD (s): 0.01; 95% CI [-0.03, 0.05]; % Change 0.42%;  SMD: 0.12; 95% CI [-0.36, 0.6] Weight 2.29%; Inference - Trivial |
| Zghal et al. (2019) A (50) | M, n=14, Unclear Soccer Players; Age 14.5±0.5 years | Power, plyometrics and unresisted sprint training 1d/wk, 7wks, 7 sessions, In-season | 2-4 sets of 6-8 reps/set LB power training (30-60%1RM) knee extensions, leg press and the back half squat + 2-4 sets of 6-8 foot contacts of plyometric training vertical, lateral and stride hurdle-jumps (30-60cm) + 1 set of 6-12 Short distance sprints (5-20m) | Soccer training 4 times/wk + competition Electronic photo cells (Brower Timing System, Salt Lake City, 174 UT, United States) | 0-5m performance ↑ MD (s): 0.05; 95% CI [0.02, 0.08]; % Change 4.95%;  SMD: 0.76; 95% CI [0.29, 1.22] Weight 4.28%; Inference - Moderate  0-10m performance = MD (s): 0.01; 95% CI [-0.02, 0.04]; % Change 0.55%;  SMD: 0.12; 95% CI [-0.28, 0.51] Weight 2.19%; Inference - Trivial  0-20m performance = MD (s): 0.03; 95% CI [-0.01, 0.07]; % Change 0.96%;  SMD: 0.26; 95% CI [-0.11, 0.63] Weight 2.47%; Inference - Small |
| Zghal et al. (2019) B (50) | M, n=9, Unclear Soccer Players; Age 14.5±0.5 years | Plyometrics and unresisted sprint training 1d/wk, 7wks, 7 sessions, In-season | 2-4 sets of 6-8 foot contacts of plyometric training vertical, lateral and stride hurdle-jumps (30-60cm) + 1 set of 6-12 Short distance sprints (5-20m) | See Zghal et al. (2019) A | 0-5m performance = MD (s): -0.01; 95% CI [-0.04, 0.02]; % Change -0.92%;  SMD: -0.18; 95% CI [-0.7, 0.34] Weight 4.15%; Inference - Trivial  0-10m performance = MD (s): 0.01; 95% CI [-0.03, 0.05]; % Change 0.54%;  SMD: 0.12; 95% CI [-0.37, 0.61] Weight 2.02%; Inference - Trivial  0-20m performance = MD (s): 0.01; 95% CI [-0.07, 0.09]; % Change 0.31%;  SMD: 0.05; 95% CI [-0.4, 0.51] Weight 2.34%; Inference - Trivial |

M = male, F = female, UB = upper body, LB = lower body, FB = full body, 1RM = one-repetition maximum BW = bodyweight, BB = barbell, KB = kettlebell, DB = dumbbell, AEL = accentuated eccentric loading, SL = single leg, PHV = peak height velocity, COD = change of direction, CMJ = countermovement jump, SMD = standardised mean difference, CI = confidence interval, MD = mean difference, % Change = percentage change, d = day, wk(s) = week(s), hr(s) = hour(s), ↑ = significant increase in sprint performance (p = < 0.05) , = = no significant change in sprint performance (p = > 0.05), ↓ = significant decrease in sprint performance (p = < 0.05), short sprints = 0-≤20m, medium sprints = 0-≤40m, long sprints 0->40m, the resistance and plyometric training intensity and volume descriptors are based descriptions from the study or previous guidelines (51-54).

# Declarations

**Ethics**

Approval was obtained from the ethics committee of Leeds Beckett University. The procedures used in this study comply with the ethical standards of the Declaration of Helsinki.

**Consent for publication**

Not applicable

**Availability of data and materials**

The datasets generated during and/or analysed during the current study are available from the corresponding author on reasonable request.

**Funding**

No sources of funding were used to assist in the preparation of this article.

**Conflicts of interest**

Ben Nicholson, Alex Dinsdale, Ben Jones and Kevin Till declare no potential conflicts of interest concerning the research, content, authorship, and/or publication of this review.

**Authors' contributions**

All the authors contributed to the manuscript, including the conception and design of the study, analysis and interpretation of the data, drafting and critically revising the manuscript, and approval for publication. All authors read and approved the final manuscript.

# References

1. Alves JMVM, Rebelo AN, Abrantes C, Sampaio J. Short-term effects of complex and contrast training in soccer players' vertical jump, sprint, and agility abilities. J Strength Cond Res. 2010;24(4):936-41.

2. Barr MJ, Gabbett TJ, Newton RU, Sheppard JM. Effect of 8 days of a hypergravity condition on the sprinting speed and lower-body power of elite rugby players. J Strength Cond Res. 2015;29(3):722-9.

3. Beato M, Bianchi M, Coratella G, Merlini M, Drust B. Effects of plyometric and directional training on speed and jump performance in elite youth soccer players. J Strength Cond Res. 2018;32(2):289-96.

4. Bianchi M, Coratella G, Dello IA, Beato M. Comparative effects of single vs. double weekly plyometric training sessions on jump, sprint and change of directions abilities of elite youth football players. J Sports Med Phys Fit. 2019;59(6):910.

5. Brito J, Vasconcellos F, Oliveira J, Krustrup P, Rebelo A. Short-term performance effects of three different low-volume strength-training programmes in college male soccer players. J Hum Kinet. 2014;40(1):121-8.

6. Cavaco B, Sousa N, dos Reis VM, Garrido N, Saavedra F, Mendes R, et al. Short-term effects of complex training on agility with the ball, speed, efficiency of crossing and shooting in youth soccer players. J Hum Kinet. 2014;43(1):105-12.

7. Corrêa DA, Soares DS, Gonelli PRG, Cesar MdC, Germano MD, Sindorf MAG, et al. Effect of 29 weeks of periodized soccer training on the neuromuscular performance of soccer players under 20 years of age. J Exerc Physiol Online. 2016;19(4):32-41.

8. Coutts A, Reaburn P, Piva TJ, Murphy A. Changes in selected biochemical, muscular strength, power, and endurance measures during deliberate overreaching and tapering in rugby league players. Int J Sports Med. 2007;28(2):116-24.

9. de Hoyo M, Gonzalo-Skok O, Sañudo B, Carrascal C, Plaza-Armas JR, Camacho-Candil F, et al. Comparative effects of in-season full-back squat, resisted sprint training, and plyometric training on explosive performance in U-19 elite soccer players. J Strength Cond Res. 2016;30(2):368-77.

10. de Villarreal ES, Suarez-Arrones L, Requena B, Haff GG, Ferrete C. Effects of plyometric and sprint training on physical and technical skill performance in adolescent soccer players. J Strength Cond Res. 2015;29(7):1894-903.

11. Enoksen E, Staxrud M, Tønnessen E, Shalfawi S. The effect of supervised strength training on young elite male soccer players’ physical performance. Serbian J Sports Sci. 2013;7(4):173-9.

12. Faude O, Roth R, Di Giovine D, Zahner L, Donath L. Combined strength and power training in high-level amateur football during the competitive season: a randomised-controlled trial. J Sports Sci. 2013;31(13):1460-7.

13. Franco-Marquez F, Rodriguez-Rosell D, Gonzalez-Suarez J, Pareja-Blanco F, Mora-Custodio R, Yanez-Garcia J, et al. Effects of combined resistance training and plyometrics on physical performance in young soccer players. Int J Sports Med. 2015;94(11):906-14.

14. Gil S, Barroso R, Crivoi do Carmo E, Loturco I, Kobal R, Tricoli V, et al. Effects of resisted sprint training on sprinting ability and change of direction speed in professional soccer players. J Sports Sci. 2018;36(17):1923-9.

15. González-Badillo JJ, Pareja-Blanco F, Rodríguez-Rosell D, Abad-Herencia JL, del Ojo-López JJ, Sánchez-Medina L. Effects of velocity-based resistance training on young soccer players of different ages. J Strength Cond Res. 2015;29(5):1329-38.

16. Gorostiaga E, Izquierdo M, Ruesta M, Iribarren J, Gonzalez-Badillo J, Ibanez J. Strength training effects on physical performance and serum hormones in young soccer players. Eur J Appl Physiol. 2004;91(5-6):698-707.

17. Hammami R, Granacher URS, Makhlouf I, Behm DG, Chaouachi A. Sequencing effects of balance and plyometric training on physical performance in youth soccer athletes. J Strength Cond Res. 2016;30(12):3278-89.

18. Hammami M, Gaamouri N, Shephard RJ, Chelly MS. Effects of contrast strength vs. plyometric training on lower limb explosive performance, ability to change direction and neuromuscular adaptation in soccer players. J Strength Cond Res. 2018.

19. Harris NK, Cronin JB, Hopkins WG, Hansen KT. Squat jump training at maximal power loads vs. heavy loads: effect on sprint ability. J Strength Cond Res. 2008;22(6):1742-9.

20. Harrison AJ, Bourke G. The effect of resisted sprint training on speed and strength performance in male rugby players. J Strength Cond Res. 2009;23(1):275-83.

21. Koundourakis NE, Androulakis N, Spyridaki EC, Castanas E, Malliaraki N, Tsatsanis C, et al. Effect of different seasonal strength training protocols on circulating androgen levels and performance parameters in professional soccer players. Horm. 2014;13(1):104-18.

22. Lockie RG, Murphy AJ, Scott BR, Janse de Jonge XA. Quantifying session ratings of perceived exertion for field-based speed training methods in team sport athletes. J Strength Cond Res. 2012;26(10):2721-8.

23. Lockie RG, Murphy AJ, Schultz AB, Knight TJ, de Jonge XAJ. The effects of different speed training protocols on sprint acceleration kinematics and muscle strength and power in field sport athletes. J Strength Cond Res. 2012;26(6):1539-50.

24. Lockie RG, Murphy AJ, Callaghan SJ, Jeffriess MD. Effects of sprint and plyometrics training on field sport acceleration technique. J Strength Cond Res. 2014;28(7):1790-801.

25. López-Segovia M, Andrés JMP, González-Badillo JJ. Effect of 4 months of training on aerobic power, strength, and acceleration in two under-19 soccer teams. J Strength Cond Res. 2010;24(10):2705-14.

26. Los Arcos A, Yanci J, Mendiguchia J, Salinero JJ, Brughelli M, Castagna C. Short-term training effects of vertically and horizontally oriented exercises on neuromuscular performance in professional soccer players. Int J Sports Physiol Perform. 2014;9(3):480-8.

27. Loturco I, Kobal R, Kitamura K, Cal Abad CC, Faust B, Almeida L, et al. Mixed training methods: effects of combining resisted sprints or plyometrics with optimum power loads on sprint and agility performance in professional soccer players. Front Physiol. 2017;8:1034.

28. Marques DL, Travassos B, Sousa AC, Gil MH, Ribeiro JN, Marques MC. Effects of low-moderate load high-velocity resistance training on physical performance of under-20 futsal players. Sports. 2019;7(3).

29. McMorrow BJ, Ditroilo M, Egan B. Effect of heavy resisted sled sprint training during the competitive season on sprint and change-of-direction performance in professional soccer players. Int J Sports Physiol Perform. 2019;14(8):1066-73.

30. Mendiguchia J, Martinez-Ruiz E, Morin JB, Samozino P, Edouard P, Alcaraz PE, et al. Effects of hamstring-emphasized neuromuscular training on strength and sprinting mechanics in football players. Scand J Med Sci Sports. 2015;25(6):e621-e9.

31. Michailidis Y, Tabouris A, Metaxas T. Effects of plyometric and directional training on physical fitness parameters in youth soccer players. Int J Sports Physiol Perform. 2019;14(3):392-8.

32. Mujika I, Santisteban J, Castagna C. In-season effect of short-term sprint and power training programs on elite junior soccer players. J Strength Cond Res. 2009;23(9):2581-7.

33. Nonnato A, Hulton AT, Brownlee TE, Beato M. The Effect of a Single Session of Plyometric Training Per Week on Fitness Parameters in Professional Female Soccer Players: A Randomized Controlled Trial. J Strength Cond Res. 2020;Publish Ahead of Print.

34. Ozbar N. Effects of plyometric training on explosive strength, speed and kicking speed in female soccer players. Anthropol. 2015;19(2):333-9.

35. Ozbar N, Ates S, Agopyan A. The effect of 8-week plyometric training on leg power, jump and sprint performance in female soccer players. J Strength Cond Res. 2014;28(10):2888-94.

36. Rey E, PadrÓN-Cabo A, FernÁNdez-Penedo D. Effects of sprint training with and without weighted vest on speed and repeated sprint ability in male soccer players. J Strength Cond Res. 2017;31(10):2659-66.

37. Rodríguez-Rosell D, Franco-Márquez F, Pareja-Blanco F, Mora-Custodio R, Yáñez-García JM, González-Suárez JM, et al. Effects of 6 weeks resistance training combined with plyometric and speed exercises on physical performance of pre-peak-height-velocity soccer players. Int J Physiol Perform. 2016;11(2):240-6.

38. Rodríguez-Rosell D, Franco-MÁrquez F, Mora-Custodio R, GonzÁlez-Badillo JJ. Effect of high-speed strength training on physical performance in young soccer players of different ages. J Strength Cond Res. 2017;31(9):2498-508.

39. Rodríguez-Rosell D, Torres-Torrelo J, Franco-Márquez F, González-Suárez JM, González-Badillo JJ. Effects of light-load maximal lifting velocity weight training vs. combined weight training and plyometrics on sprint, vertical jump and strength performance in adult soccer players. J Sci Med Sport. 2017;20(7):695-9.

40. Ross A, Gill N, Cronin J, Cross M. The effects of two power training programmes on the sprint speed, mechanical sprint characteristics, and lower body power of rugby sevens players [Doctor of Philosophy (PhD)]. New Zealand: Auckland University of Technology; 2015.

41. Seitz LB, Barr M, Haff GG. Effects of sprint training with or without ball carry in elite rugby players. Int J Sports Physiol Perform. 2015;10(6):761-6.

42. Shalfawi SA, Ingebrigtsen J, Dillern T, Tønnessen E, Delp TK, Enoksen E. The effect of 40 m repeated sprint training on physical performance in young elite male soccer players. Serbian J Sports Sci. 2012;6(3).

43. Spinks CD, Murphy AJ, Spinks WL, Lockie RG. The effects of resisted sprint training on acceleration performance and kinematics in soccer, rugby union, and Australian football players. J Strength Cond Res. 2007;21(1):77-85.

44. Tønnessen E, Shalfawi SA, Haugen T, Enoksen E. The effect of 40-m repeated sprint training on maximum sprinting speed, repeated sprint speed endurance, vertical jump, and aerobic capacity in young elite male soccer players. J Strength Cond Res. 2011;25(9):2364-70.

45. Tous-Fajardo J, Gonzalo-Skok O, Arjol-Serrano JL, Tesch P. Enhancing change-of-direction speed in soccer players by functional inertial eccentric overload and vibration training. Int J Sports Physiol Perform. 2016;11(1):66-73.

46. Weakley J, Till K, Sampson J, Banyard H, Leduc C, Wilson K, et al. The effects of augmented feedback on sprint, jump, and strength adaptations in rugby union players following a four week training programme. Int J Sports Physiol Perform. 2019:1-21.

47. West DJ, Cunningham DJ, Bracken RM, Bevan HR, Crewther BT, Cook CJ, et al. Effects of resisted sprint training on acceleration in professional rugby union players. J Strength Cond Res. 2013;27(4):1014-8.

48. Winwood PW, Cronin JB, Posthumus LR, Finlayson SJ, Gill ND, Keogh JW. Strongman vs. traditional resistance training effects on muscular function and performance. J Strength Cond Res. 2015;29(2):429-39.

49. Yanci J, Los Arcos A, Camara J, Castillo D, García A, Castagna C. Effects of horizontal plyometric training volume on soccer players' performance. Res Sports Med. 2016;24(4):308-19.

50. Zghal F, Colson SS, Blain G, Behm DG, Granacher U, Chaouachi A. Combined resistance and plyometric training is more effective than plyometric training alone for improving physical fitness of pubertal soccer players. Front Physiol. 2019;10:1026-.

51. Chu DA. Jumping into plyometrics: Hum Kinet; 1998.

52. Haff GG, Triplett NT. Essentials of strength training and conditioning 4th edition: Hum Kinet; 2015.

53. Brearley S, Wild J, Agar-Newman D, Cizmic H. How to monitor net plyometric training stress: Guidelines for the coach. Prof Strength Cond. 2017;47:15-24.

54. Ebben WP. Practical guidelines for plyometric intensity. 2009.
