# Supplementary material for: The Training of Short Distance Sprint Performance in Football Code Athletes: A Systematic Review and Meta-Analysis
Source: Sports Med. 2020 Nov 27;51(6):1179–207. doi: 10.1007/s40279-020-01372-y (PMC8124057; doi:10.1007/s40279-020-01372-y)
Supplement: Supplementary file 3 — Supplementary file3 (DOCX 28 kb) [file 40279_2020_1372_MOESM3_ESM.docx]

**Electronic Supplementary Material Table 3**

Article title - The Training of Short Distance Sprint Performance in Football Code Athletes: A Systematic Review and Meta-Analysis

Journal name – Sports Medicine

Author names - Ben Nicholson, Alex Dinsdale, Ben Jones, and Kevin Till.

Affiliations - Leeds Beckett University, Carnegie Applied Rugby Research (CARR) centre, Carnegie School of Sport, Leeds, United Kingdom. Yorkshire Carnegie Rugby Union club, Leeds, United Kingdom. Leeds Rhinos Rugby League club, Leeds, United Kingdom. England Performance Unit, The Rugby Football League, Leeds, United Kingdom. School of Science and Technology, University of New England, Armidale, NSW, Australia. Division of Exercise Science and Sports Medicine, Department of Human Biology, Faculty of Health Sciences, the University of Cape Town and the Sports Science Institute of South Africa, Cape Town, South Africa.

corresponding author e-mail address – b.t.nicholson@leedsbeckett.ac.uk

**Table S3**

**Characteristics of the specific sprint training groups (primary, secondary and combined primary and secondary methods) included in the review**

| **Study (year)** | **Subjects** | **Training type and organisation** | **Training methods** | **Other training and testing equipment** | **Mean difference, 95% CI, percentage change, Std. Mean Difference IV, Random, 95% CI, weight, Qualitative inference** |
| --- | --- | --- | --- | --- | --- |
| Borges et al. (2016) A (1) | M, n=9, Elite Soccer Players; Age 16±0.6 years | Resisted sprint training 1-2d/wk, 7wks, 12 sessions, In-season | 2-7 sets of short-medium distance resisted sled sprints (1 rep of at each distance/set 5m; 10m; 20m and 30 m @ 10-13% BW) | 90—150 minutes soccer training session every morning, 5 times/wk, and one official match every Saturday  Photocell system | 0-5m performance = MD (s): 0.01; 95% CI [-0.03, 0.05]; % Change 0.98%;  SMD: 0.15; 95% CI [-0.37, 0.66] Weight 22.53%; Inference - Trivial |
| Bremec (2018) A (2) | M, n=8, Sub-elite Soccer Players; Age 15.6±0.4 years | Resisted sprint training 2d/wk, 8wks, 16 sessions, Pre-season and In-season | 1 set of 3-5 reps/set of short distance resisted sprints (20m) at loads corresponding to peak power output, resisted using a 1080Sprint | 4 soccer training sessions/wk, 2 longer (75-90 mins) and 2 shorter (45-60 mins) duration and a 1 match/wk  1080Sprint (1080 Motion AB, Lidingö, Sweden) | 0-5m performance ↑ MD (s): 0.13; 95% CI [0.03, 0.23]; % Change 8.44%;  SMD: 0.7; 95% CI [0.1, 1.31] Weight 21.45%; Inference - Moderate  0-10m performance ↑ MD (s): 0.14; 95% CI [0.04, 0.24]; % Change 5.93%;  SMD: 0.75; 95% CI [0.17, 1.34] Weight 29.4%; Inference - Moderate  0-20m performance ↑ MD (s): 0.17; 95% CI [0.07, 0.27]; % Change 4.49%;  SMD: 0.82; 95% CI [0.27, 1.38] Weight 19.98%; Inference - Large |
| Bremec (2018) B (2) | M, n=10, Sub-elite Soccer Players; Age 15.6±0.5 years | Unresisted sprint training 2d/wk, 8wks, 16 sessions, Pre-season and In-season | 1 set of 8 reps/set of short distance (20m) sprints | See Bremec (2018) A | 0-5m performance = MD (s): -0.06; 95% CI [-0.18, 0.06]; % Change -3.39%;  SMD: -0.25; 95% CI [-0.75, 0.24] Weight 27.48%; Inference - Small  0-10m performance = MD (s): -0.06; 95% CI [-0.19, 0.07]; % Change -2.3%;  SMD: -0.22; 95% CI [-0.68, 0.25] Weight 34.26%; Inference - Small  0-20m performance = MD (s): -0.06; 95% CI [-0.21, 0.09]; % Change -1.48%;  SMD: -0.18; 95% CI [-0.61, 0.26] Weight 9.03%; Inference - Trivial |
| De Hoyo et al. (2016) B (3) | M, n=12, Elite Soccer Players; Age 17±1 years | Resisted sprint training 2d/wk, 8wks, 16 sessions, In-season | 3 sets of 6–10 reps/set of short loaded (12.6%) sprints (20m) on artificial grass. | 10 hrs of combined soccer (4– 5 sessions) and conditioning (1 session) training, and 1 competitive match/wk.  Dual-beam electronic timing gate OptoJump System (Polifemo Radio Light, Microgate, Bolzano, Italy) | 0-10m performance = MD (s): 0.01; 95% CI [-0.01, 0.03]; % Change 1.76%;  SMD: 0.18; 95% CI [-0.25, 0.6] Weight 41.2%; Inference - Trivial  0-20m performance = MD (s): 0.01; 95% CI [-0.02, 0.04]; % Change 2.18%;  SMD: 0.13; 95% CI [-0.26, 0.52] Weight 21.95%; Inference - Trivial |
| Derakhti et al. (2018) A (4) | M, n=8, Soccer Players; Age 15.6±0.4 years | Resisted sprint training 2d/wk, 4wks, 8 sessions, In-season | 1 set of 5 reps/set of short distance (20m) resisted sprints @ a load corresponding to a 50% reduction in Vmax (Pmax) on artificial “astro” turf. | On average, four soccer-specific sessions/Wk. Two longer sessions (75-90 min) and two were shorter (45-60 min). After the fifth session of the intervention, the participants’ regular game-season began and one competitive match (40+40min) /wk (usually Saturday or Sunday) was added to the total training volume.  1080Sprint (1080 Motion AB, Lidingö, Sweden) | 0-5m performance ↑ MD (s): 0.13; 95% CI [0.03, 0.23]; % Change 1.36%;  SMD: 0.7; 95% CI [0.1, 1.31] Weight 21.45%; Inference - Moderate  0-10m performance ↑ MD (s): 0.14; 95% CI [0.04, 0.24]; % Change 2.2%;  SMD: 0.75; 95% CI [0.17, 1.34] Weight 29.4%; Inference - Moderate  0-20m performance ↑ MD (s): 0.17; 95% CI [0.07, 0.27]; % Change 1.89%;  SMD: 0.82; 95% CI [0.27, 1.38] Weight 19.98%; Inference - Large |
| Derakhti et al. (2018) B (4) | M, n=10, Soccer Players; Age 15.6±0.5 years | Unresisted sprint training 2d/wk, 4wks, 8 sessions, In-season | 1 set of 8 reps/set of short distance sprints (20m) on artificial “astro” turf. | See Derakhti et al. (2018) A | 0-5m performance = MD (s): -0.06; 95% CI [-0.18, 0.06]; % Change -3.39%;  SMD: -0.25; 95% CI [-0.75, 0.24] Weight 27.48%; Inference - Small  0-10m performance = MD (s): -0.06; 95% CI [-0.19, 0.07]; % Change -2.3%;  SMD: -0.22; 95% CI [-0.68, 0.25] Weight 34.26%; Inference - Small  0-20m performance = MD (s): -0.06; 95% CI [-0.21, 0.09]; % Change -1.48%;  SMD: -0.18; 95% CI [-0.61, 0.26] Weight 9.03%; Inference - Trivial |
| Haugen et al. (2014) A (5) | M, n=6, F, n=7 Elite Soccer Players; Age 17±1 years | Unresisted sprint training 1d/wk, 9wks, 9 sessions, In-season | 1 set of 20-25 reps of short sprints (20m). Sprints performed @ 90% effort. | 7-11 Soccer training sessions and 1-5 soccer matches/wk. The main practices were mostly small, medium or large sided games (3 vs 3 to 11 vs 11). Technical elements were focused on during small-sided practices, while tactical drills/formations were emphasised during medium and large sided practices  Timings gates | 0-20m performance = MD (s): 0.03; 95% CI [-0.03, 0.09]; % Change 0.97%;  SMD: 0.19; 95% CI [-0.19, 0.57] Weight 11.72%; Inference - Trivial |
| Haugen et al. (2015) A (6) | M, n=13, Elite Soccer Players; Age 17±1 years | Unresisted sprint training 2d/wk, 7wks, 7 sessions, Pre-season | 1 set of 15 reps/set of short sprints (20m). Sprints performed @ 100% effort with no supervision | Participants were requested to refrain from performing any other off-field physical training regimes in terms of speed, strength and/or endurance.  Timing Gates | 0-20m performance = MD (s): 0.01; 95% CI [-0.05, 0.07]; % Change 0.34%;  SMD: 0.07; 95% CI [-0.31, 0.44] Weight 11.9%; Inference - Trivial |
| Haugen et al. (2015) B (6) | M, n=13, Elite Soccer Players; Age 17±1 years | Unresisted sprint training 1d/wk, 7wks, 7 sessions, Pre-season | 1 set of 30 reps of short sprints (20m). Sprints performed @ 90% effort with no supervision | See Haugen et al. (2015) A | 0-20m performance = MD (s): 0.01; 95% CI [-0.03, 0.05]; % Change 0.26%;  SMD: 0.09; 95% CI [-0.29, 0.46] Weight 11.88%; Inference - Trivial |
| Haugen et al. (2015) C (6) | M, n=10, Elite Soccer Players; Age 17±1 years | Unresisted sprint training 1d/wk, 7wks, 7 sessions, Pre-season | 1 set of 30 reps of short sprints (20m). Sprints performed @ 90% effort with direct supervision | See Haugen et al. (2015) A | 0-20m performance = MD (s): -0.01; 95% CI [-0.05, 0.03]; % Change -0.25%;  SMD: -0.11; 95% CI [-0.54, 0.32] Weight 9.12%; Inference - Trivial |
| Mathisen and Danielsen (2014) A (7) | F, n=13, Sub-elite Soccer Players; Age 13.6±0.2 years | Resisted and unresisted sprint training 1d/wk, 8wks, 8 sessions, Pre-season | 1 set of 8 reps/set of short distance (20m) sprinting + 1 set of 8 reps/set of short distance partner resisted sprinting (15m) + 1 set of 8 reps/set of short distance sprints with COD (15m) | 2x/wk 1hr soccer training sessions, consisting of technical drills and small-sided games  Electronic photocells timing gates (Brower Timing System, USA) | 0-10m performance ↑ MD (s): 0.11; 95% CI [0.06, 0.16]; % Change 5.45%;  SMD: 0.99; 95% CI [0.49, 1.48] Weight 55.64%; Inference - Large  0-20m performance = MD (s): 0; 95% CI [-0.04, 0.04]; % Change 0%;  SMD: 0; 95% CI [-0.38, 0.38] Weight 36.7%; Inference - Trivial |
| Mathisen and Pettersen (2015) A (8) | F, n=10, Elite Soccer Players; Age 15.5±0.7 years | Resisted and unresisted sprint training 1d/wk, 8wks, 8 sessions, Phase not reported | 1 set of 8 reps/set of short distance (20m) sprinting + 1 set of 8 reps/set of short distance partner resisted sprinting (15m) + 1 set of 8 reps/set of short distance sprints with COD (20m) | 2x/wk 1hr soccer training sessions, consisting of technical drills and small-sided games  Electronic photocells timing gates (Brower Timing System, USA) | 0-10m performance ↑ MD (s): 0.08; 95% CI [0.04, 0.12]; % Change 4.19%;  SMD: 0.93; 95% CI [0.38, 1.49] Weight 44.36%; Inference - Large  0-20m performance ↑ MD (s): 0.11; 95% CI [0.04, 0.18]; % Change 3.22%;  SMD: 0.71; 95% CI [0.23, 1.19] Weight 30.53%; Inference - Moderate |
| Morin et al. (2017) A (9) | M, n=6, Elite Soccer Players; Age 25.5±3.2 years | Resisted and unresisted sprint training 2d/wk, 8wks, 16 sessions, In-season | 2 sets of 5 reps of combined un-resisted (5-1 reps) and resisted (5-9 reps at 80% BW) short sprints (20m) | Two 2hr training sessions/wk plus one game /wk.  (Stalker ATS Pro II, Applied Concepts, TX, USA) | 0-5m performance ↑ MD (s): 0.03; 95% CI [0, 0.06]; % Change 2.14%;  SMD: 0.54; 95% CI [0.02, 1.07] Weight 100%; Inference - Moderate  0-20m performance = MD (s): 0.04; 95% CI [-0.01, 0.09]; % Change 1.14%;  SMD: 0.33; 95% CI [-0.11, 0.77] Weight 32.78%; Inference - Small |
| Morin et al. (2017) B (9) | M, n=10, Sub-elite Soccer Players; Age 26.3±4 years | Unresisted sprint training 2d/wk, 8wks, 16 sessions, In-season | 2 sets of 5 reps of un-resisted short sprints of 20m | See Morin et al. (2017) A | 0-5m performance = MD (s): 0.01; 95% CI [-0.03, 0.05]; % Change 0.71%;  SMD: 0.18; 95% CI [-0.46, 0.81] Weight 16.75%; Inference - Trivial  0-20m performance = MD (s): 0.02; 95% CI [-0.06, 0.1]; % Change 0.57%;  SMD: 0.15; 95% CI [-0.41, 0.7] Weight 5.45%; Inference - Trivial |
| Mujika et al. (2009) B (10) | M, n=6, Sub-elite Soccer Players; Age 26.8±4.2 years | Unresisted sprint training 1d/wk, 6wks, 6 sessions, In-season | 2-4 sets of 4 reps/set of medium length sprints (30m) | N/A  Photocell gates (Timer S4, Alge-Timing, Lustenau, Austria) | 0-20m performance = MD (m·s m·s^-1^): -0.03; 95% CI [-0.11, 0.05]; % Change -0.32%;  SMD: -0.15; 95% CI [-0.59, 0.28] Weight 9.07%; Inference - Trivial |
| Rimmer and Sleivert (2000) B (11) | M, n=10, Elite Soccer Players; Age 18.1±0.5 years | Unresisted sprint training 1-2d/wk, 8wks, 15 sessions, Phase not reported | Unresisted sprinting for medium length sprints 2-5 sets of 2-8 reps/set (25-55 m) on a grass surface. | N/A  Digital timer (University of Otago, Dunedin, New Zealand) | 0-10m performance = MD (s): 0.02; 95% CI [-0.01, 0.05]; % Change 1.04%;  SMD: 0.35; 95% CI [-0.15, 0.86] Weight 31.48%; Inference - Small  0-20m performance = MD (s): 0.01; 95% CI [0, 0.02]; % Change 0.78%;  SMD: 0.35; 95% CI [-0.11, 0.82] Weight 7.78%; Inference - Small |
| Upton (2011) A (12) | M, n=9, Sub-elite Rugby and touch rugby Players; Age 24.4±4 years | Assisted sprint training 3d/wk, 4wks, 12 sessions, Pre-season | Assisted maximal sprinting (1 set of 10 sprints/set) of short sprints (18.3m). Assistive load @14.7%BW (45-95 assistive force). Sprints were assisted by a bungy cord shoulder harness | 6 d/wk (~1.5hrs) ball-handling skills, defensive drills, offensive drills, and small-sided games.  Infrared beam timing system (Brower Timing Systems Speed Trap II, Salt Lake City, UT, USA) | 0-5m performance ↑ MD (m·s^-1^): 0.38; 95% CI [0.32, 0.44]; % Change 2.74%;  SMD: 3.45; 95% CI [2.01, 4.89] Weight 12.03%; Inference - Large  0-20m performance ↑ MD (m·s^-1^): 0.21; 95% CI [0.16, 0.26]; % Change 0.33%;  SMD: 1.85; 95% CI [1.06, 2.65] Weight 16.86%; Inference - Large |
| Upton (2011) B (12) | F, n=8 Elite Soccer Players; Age 19.6±0.9 years | Resisted sprint training 3d/wk, 4wks, 12 sessions, Pre-season | Resisted maximal sprinting (1 set of 10 sprints/set) of short sprints (18.3m). Resistance load @10% dec in Vmax (12.6%Bw). Sprints were resisted by a "The trainer (Perform better) by a waist harness" | See Upton (2011) A | 0-5m performance = MD (m·s^-1^): 0; 95% CI [-0.08, 0.08]; % Change 8.44%;  SMD: 0; 95% CI [-0.51, 0.51] Weight 22.56%; Inference - Trivial  0-20m performance = MD (m·s^-1^): -0.03; 95% CI [-0.12, 0.06]; % Change 4.49%;  SMD: -0.15; 95% CI [-0.61, 0.3] Weight 21.24%; Inference - Trivial |
| Upton (2011) C (12) | F, n=9 Elite Soccer Players; Age 19.6±0.9 years | Unresisted sprint training 3d/wk, 4wks, 12 sessions, Pre-season | Maximal sprinting (1 set of 10 sprints/set) of short sprints (18.3m). | See Upton (2011) A | 0-5m performance = MD (m·s^-1^): -0.02; 95% CI [-0.16, 0.12]; % Change -0.54%;  SMD: -0.07; 95% CI [-0.56, 0.42] Weight 28.29%; Inference - Trivial  0-20m performance = MD (m·s^-1^): 0.02; 95% CI [-0.11, 0.15]; % Change 0.39%;  SMD: 0.07; 95% CI [-0.36, 0.5] Weight 9.15%; Inference - Trivial |
| Venturelli et al. (2008) (13) | F, n=10 Elite Soccer Players; Age 19.6±0.9 years | Unresisted sprint training 2d/wk, 12wks, 24 sessions, Phase not reported | Maximal sprinting (1 set of 20 sprints/set) of short sprints (10-20m). | Not available | 0-20m performance = MD (s): 0.09; 95% CI [-0.02, 0.2]; % Change 2.46%;  SMD: 0.43; 95% CI [-0.1, 0.97] Weight 5.87%; Inference - Moderate |

M = male, F = female, N/A = data not available, short sprints = 0-≤20m, medium sprints = 0-≤40m, long sprints 0->40m, SMD = standardised mean difference, CI = confidence interval, MD = mean difference, % Change = percentage change, d = day, wk(s) = week(s), hr(s) = hour(s), ↑ = significant increase in sprint performance (p = < 0.05) , = = no significant change in sprint performance (p = > 0.05), ↓ = significant decrease in sprint performance (p = < 0.05).

# Declarations

**Ethics**

Approval was obtained from the ethics committee of Leeds Beckett University. The procedures used in this study comply with the ethical standards of the Declaration of Helsinki.

**Consent for publication**

Not applicable

**Availability of data and materials**

The datasets generated during and/or analysed during the current study are available from the corresponding author on reasonable request.

**Funding**

No sources of funding were used to assist in the preparation of this article.

**Conflicts of interest**

Ben Nicholson, Alex Dinsdale, Ben Jones and Kevin Till declare no potential conflicts of interest concerning the research, content, authorship, and/or publication of this review.

**Authors' contributions**

All the authors contributed to the manuscript, including the conception and design of the study, analysis and interpretation of the data, drafting and critically revising the manuscript, and approval for publication. All authors read and approved the final manuscript.

# References

1. Borges JH, Conceição MS, Vechin FC, Pascoal EHF, Silva RP, Borin JP. The effects of resisted sprint vs. plyometric training on sprint performance and repeated sprint ability during the final weeks of the youth soccer season. Sci Sports. 2016;31(4):e101-e5.

2. Bremec D. Very heavy resisted sprinting: a better way to improve acceleration?: effects of a 4-week very heavy resisted sprinting intervention on acceleration, sprint and jump performance in youth soccer players [Masters Thesis]: Swedish School of Sport and Health Sciences; 2018.

3. de Hoyo M, Gonzalo-Skok O, Sañudo B, Carrascal C, Plaza-Armas JR, Camacho-Candil F, et al. Comparative effects of in-season full-back squat, resisted sprint training, and plyometric training on explosive performance in U-19 elite soccer players. J Strength Cond Res. 2016;30(2):368-77.

4. Derakhti M. Very heavy resisted sprint training for adolescent football players: a training intervention on acceleration, sprint and jump performance in late pubertal adolescent athletes [Masters of sports science]: Swedish School of sport and health science; 2018.

5. Haugen T, Tonnessen E, Leirstein S, Hem E, Seiler S. Not quite so fast: effect of training at 90% sprint speed on maximal and repeated-sprint ability in soccer players. J Sports Sci. 2014;32(20):1979-86.

6. Haugen T, Tønnessen E, Øksenholt Ø, Haugen FL, Paulsen G, Enoksen E, et al. Sprint conditioning of junior soccer players: effects of training intensity and technique supervision. Plos One. 2015;10(3):e0121827-e.

7. Mathisen GE, Danielsen KH. Effects of speed exercises on acceleration and agility performance in 13-year-old female soccer players. J Phys Educ Sport. 2014;14(4):471-4.

8. Mathisen GE, Svein AP. The effect of speed training on sprint and agility performance in female youth soccer players. J Phys Educ Sport. 2015;15(3):395-9.

9. Morin J-B, Petrakos G, Jiménez-Reyes P, Brown SR, Samozino P, Cross MR. Very-heavy sled training for improving horizontal-force output in soccer players. Int J Sports Physiol Perform. 2017;12(6):840-4.

10. Mujika I, Santisteban J, Castagna C. In-season effect of short-term sprint and power training programs on elite junior soccer players. J Strength Cond Res. 2009;23(9):2581-7.

11. Rimmer E, Sleivert G. Effects of a plyometrics intervention program on sprint performance. J Strength Cond Res. 2000;14(3):295-301.

12. Upton DE. The effect of assisted and resisted sprint training on acceleration and velocity in Division IA female soccer athletes. J Strength Cond Res. 2011;25(10):2645-52.

13. Venturelli M, Bishop D, Pettene L. Sprint training in preadolescent soccer players. Int J Sports Physiol Perform. 2008;3(4):558-62.
